# Traditional Chinese medicine for diabetic peripheral neuropathy: a network meta-analysis

**DOI:** 10.3389/fendo.2025.1596924

**Published:** 2025-08-27

**Authors:** Jinglin Hu, Haili Wang, Xiaogang Hao, Ting Pan, Xuefeng Li, Xue Zhou, Siyi Wang, Yubo Gong, Lingfeng Wu, Shuo Dong, Xinhua Chen, Xichen Wang

**Affiliations:** ^1^ College of Acupuncture and Tuina, Changchun University of Chinese Medicine, Changchun, Jilin, China; ^2^ Department of Acupuncture and Tuina, Affiliated Hospital of Changchun University of Traditional Chinese Medicine, Changchun, Jilin, China; ^3^ School of Medical Information, Changchun University of Chinese Medicine, Changchun, Jilin, China

**Keywords:** traditional Chinese medicine, TCM, diabetic peripheral neuropathy, network meta-analysis, meta-analysis

## Abstract

**Background:**

Diabetic peripheral neuropathy (DPN) is a common complication of diabetes mellitus, characterized by high morbidity and significant disability. Traditional Chinese medicine (TCM) has shown potential in relieving symptoms and improving neurological function through multi-targeted mechanisms; however, the efficacy and safety of different TCM therapies have yet to be systematically evaluated.

**Objective:**

This study aims to provide evidence-based medicine for treating DPN with TCM therapy by network meta-analysis (NMA).

**Methods:**

This study comprehensively searched nine databases constructed up to November 2024. The quality and evidence of the included RCTs were assessed using the risk of bias assessment tool and GRADE pro, and pairwise meta-analysis and NMA were performed using RevMan, Stata, and R Studio. The results showed that 95 RCTs involving 8194 patients were included, containing 9 TCM therapies.

**Results:**

TCM Decoration + Acupuncture ranked highest in improving the motor conduction velocity of the common peroneal nerve (SUCRA = 0.81), followed by TCM Decoction + Chinese Herbal Footbath (SUCRA = 0.80), electroacupuncture (SUCRA = 0.75). Regarding the sensory conduction velocity of the common peroneal nerve, TCM Decoration + Chinese Herbal Foot (SUCRA=0.87) ranked first, followed by TCM Decoction + Acupuncture (SUCRA = 0.83), and TCM Decoction (SUCRA = 0.51). Electroacupuncture (SUCRA = 0.83) ranks first in improving median nerve motor conduction velocity, followed by TCM Decoction + Acupuncture (SCURA = 0.98), TCM Decoction (SUCRA = 0.55). TCM Decoration + Acupuncture (SUCRA=0.98) ranks first in improving the sensory conduction velocity of the median nerve, followed by electroacupuncture (SUCRA = 0.51), and Chinese Patent Medicine (SUCRA = 0.51). TCM Decoration + Chinese Herbal Footbath (SUCRA = 0.85) ranked first in improving overall clinical symptoms of DPN.

**Conclusion:**

The effectiveness and safety of traditional Chinese medicine therapy in treating DPN have been preliminarily verified. In clinical practice, conservative clinical stratification selection can be made based on the results of this study and the actual situation. In addition, due to the limited quality of the included studies, larger sample sizes and high-quality research are still needed.

**Systematic review registration:**

https://www.crd.york.ac.uk/PROSPERO/, identifier (CRD42024589159).

## Introduction

About 5.37 million people worldwide have diabetes, and in 2045, it is expected to reach 783 million (1). Diabetic peripheral neuropathy (DPN) is one of the common complications of type 1 and type 2 diabetes mellitus. DPN is characterized by peripheral nerve involvement in the lower limbs, with symmetrical numbness, discomfort, and pain in the lower limbs, which often starts from the feet, spreads upward to the calves, and later spreads to the upper limbs. Clinical signs include profound sensory deficits, such as decreased or absent Achilles tendon reflex, knee tendon reflex, and positional and vibration senses, which often lead to gait and balance dysfunction (2). A meta-analysis including 29 randomized controlled trials with a total of 50,112 patients showed that the prevalence of DPN was strongly associated with age, duration of disease, and body weight of diabetic patients, with an overall prevalence of approximately 30%, and that the prevalence of DPN was higher in type 2 diabetes mellitus (31.5%) than in type 1 diabetes mellitus (17.5%) (3). DPN has become one of the key factors triggering diabetic foot ulcers, which in severe cases will lead to lower limb amputation or death. According to statistics, the total cost of lower extremity foot ulcers and amputations due to diabetes mellitus in the United States is as high as 460 million - 1.37 billion dollars, and surveys in some European countries have found that the personal cost of amputations due to DPN amounts to 83728 dollars (4, 5). Research indicates that patients with diabetes who undergo amputation face a 68% mortality rate within five years (6). This high mortality rate highlights how DPN not only diminishes patients’ quality of life but also imposes significant financial burdens on families and society. As a result, considerable focus has been on finding effective treatments for DPN. The pathogenesis of DPN is complex. It is believed that metabolic disorders due to blood glucose and dyslipidemia can lead to the development of DPN by increasing oxidative stress, inflammation and insulin resistance, and through specific signaling pathways that can lead to demyelination or neuronal damage (7). There is currently no effective treatment for DPN, and the primary goal is to improve clinical symptoms. While controlling blood glucose levels can significantly enhance DPN that arises from type 1 diabetes, it appears to have minimal impact on DPN resulting from type 2 diabetes (8, 9). Pregabalin is considered a level-A treatment for DPN and is commonly prescribed for managing the neuropathic pain associated with this condition (8). However, several adverse effects should not be overlooked. A systematic review involving 38 randomized controlled trials found that the side effects of pregabalin include dizziness, blurred vision, drowsiness, cognitive impairment, and decreased coordination. These adverse effects are related to the dosage selected. Additionally, the economic cost of the medication is a significant factor limiting its use (10). Gabapentin, another recommended medication, is limited in its use in elderly patients due to the higher doses used to achieve therapeutic effects (11). Moreover, adverse reactions still occur (12). Tricyclic antidepressants have demonstrated some efficacy for neuropathic pain in a few small randomized controlled trials. However, the evidence remains insufficient, and side effects significantly limit their use (13, 14). The evidence regarding the long-term effectiveness of opioids, despite their strong short-term pain-relieving effects, is limited. Similar to tricyclic antidepressants, the potential for addiction and the side effects of long-term opioid use are concerns for many patients. Overall, these medications have minimal difference, with only one-third of patients experiencing symptomatic improvement. Additionally, there are numerous contraindications associated with their use (15). It is crucial to intervene early in DPN to slow the progression of the disease and prevent further nerve cell damage. As a result, there is an urgent need to find a safe and effective treatment to address the challenges faced by DPN patients.

TCM therapies have made some progress in treating DPN. TCM provides a range of internal and external treatments and focuses on assessing their effectiveness through various factors, including neurophysiology, blood rheology, levels of inflammatory markers, and glycolipid metabolism (16, 17). The advantage of TCM therapies is that they can target multiple targets and play a positive role in developing and treating DPN through multiple pathways. These therapies may work through various mechanisms, including reducing inflammation, oxidative stress, and apoptosis and alleviating endoplasmic reticulum stress; additionally, TCM can improve mitochondrial function and help regulate gut microbiota (18–20). In recent years, interventions in TCM for treating DPN have been classified into three main categories: single-drug extracts, herbal decoctions, and Chinese patent medicines. External therapies include acupuncture, Chinese herbal footbath, acupoint injections, and combinations of these methods. Although these therapies show promise in treating DPN, it remains unclear whether there are significant differences among the various therapeutic approaches and which intervention may be the most effective (19, 21). To evaluate the efficacy and safety of TCM therapies for DPN and identify the optimal treatment regimen, we will conduct direct and indirect comparisons of TCM protocols utilized for DPN thus far. We aim to provide evidence-based medical information to support the use of TCM therapies in treating DPN.

## Methods

This systematic review and meta-analysis will adhere to the latest 2020 guidelines for systematic reviews and the PRISMA checklist (**Supplementary Table S1**). Before commencing this study, only a preliminary search was conducted to evaluate the feasibility and scope of potential studies. This initial search did not involve the formal screening process, data extraction, or analysis procedures. The protocol for this systematic review was registered on PROSPERO (registration number CRD42024589159) on September 10, 2024.

### Eligibility criteria

We only analyzed randomized controlled trials of TCM therapies for treating DPN. RCTs were required to have the following inclusion criteria: (1) the diagnosis of patients with DPN should be precise (no restriction on age, gender, and duration of the disease); (2) the interventions in the treatment group were TCM therapies, including internal treatments of TCM (TCM Decoction, Chinese Patent Medicine, single herbs, etc.), external treatments of TCM (Chinese Herbal Footbath, acupuncture, electroacupuncture, etc.), or their combination; (3) The control group’s interventions were Western medicines only.

We will exclude the following studies: (1) duplicate studies, (2) reviews, animal experiments, protocols, conference papers, dissertations, and case reports; (3) studies that were not formally published; and (4) studies with incomplete data.

### Information sources

We searched the following databases for RCTs of TCM therapies for the treatment of DPN (searches were performed until November 2024): PubMed, Cochrane Library, Embase, Web of Science, Medline, China National Knowledge Infrastructure (CNKI), Wanfang Data Knowledge Service Platform (Wanfang), VIP Database (VIP), and China Biology Medicine disc, (CBM disc). A detailed search strategy was completed on November 20, 2024, and some of the search strategies are documented in **Supplementary Table S2**.

### Selection of studies and data collection

Three reviewers completed this process. Two reviewers (Yubo Gong and Xiaogang Hao) first excluded the duplicate studies used and then skimmed the titles and abstracts of the remaining studies. After excluding some studies that did not fit the topic, the full text of the remaining studies was carefully read to ensure data availability. Disagreements during the process will be addressed through discussion to reach a consensus. If these disagreements cannot be resolved, the third reviewer (Xuefeng Li) will make a decision after conducting an independent review. For the studies that met the inclusion criteria, we will extract the first author, year of publication, disease duration, age, sample size, intervention, and outcome indicators. We will use electromyography (EMG) results as the primary outcome indicator, and secondary outcome indicators may also include the Toronto Clinical Scoring System (TCSS), blood glucose, glycosylated hemoglobin (HbA1c), and total effective rate.

### Risk of bias assessment

We used the risk of bias assessment tool recommended by the Cochrane Handbook to evaluate the risk of bias in the final included studies. The tool evaluated seven aspects of random generation: Random sequence generation, allocation concealment, blinding of patients and personnel, blinding of outcome assessment, incomplete outcome data, selective reporting, and other biases in the studies. The assessment of risk of bias will be reported as low risk, high risk, and unclear (**Supplementary Table S3**). Two reviewers (Ting Pan and Xue Zhou) will complete the assessment and discuss disagreements. If it is not resolved, a third reviewer (Siyi Wang) will assess and finalize it.

### Synthesis methods

First, pairwise meta-analysis was performed using Review Manager 5.4 software. The OR (odds ratio) and MD (mean difference) values were used to analyze dichotomous and continuous variables. Cochran’s I-square (I^2^) was used to determine the heterogeneity. If I^2^ < 50%, it indicates no significant statistical heterogeneity among the studies, and a fixed-effect model should be adopted. Otherwise, a random-effects model will be applied.

For network meta-analysis (NMA), we used Stata 15.0 and RStudio software. Stata 15.0 was used to generate network evidence plots and funnel plots and conduct a sensitivity analysis. The “mvmeta” package in Stata was utilized to create network evidence plots, visually illustrating the relationships between different interventions. In these plots, each point represents an intervention, and the size of the node indicates the sample size. A line connecting two nodes signifies a direct comparison between the two interventions. In contrast, the thickness of the line reflects the sample size for those interventions that have a direct comparison. When there is a closed loop, the node-splitting method is required to assess whether the results are inconsistent. Funnel plots were used for publication bias, while Egger’s test was used for validation to prove the absence of publication bias when *P* > 0.05. The data were analyzed using the “gemtc” and “JAGs-4.3.1” packages in RStudio, and the consistency model was fitted based on the Markov chain Monte Carlo method (MCMC) framework to fit the consistency model. The consistency model was used for testing when *P* > 0.05, and vice versa, using the inconsistency model. The consistency model was used for testing when *P* > 0.05, and vice versa, using the inconsistency model. Binary variables were analyzed using OR values as effect sizes, continuous variables were analyzed using MD, and the 95% CI (Confidence interval) value of the effect sizes was calculated, with a 95% CI that did not contain one considered statistically significant. The potential scale reduction factor (PSRF) was used to assess the convergence effect of the results of the reticulated meta-analysis. When the PSRF value is closer to 1, it indicates a better convergence result and more reliable results. A table of two-by-two comparisons will be generated for the final results, and the surface under the cumulative ranking curve (SUCRA) values will be used to rank the interventions. SUCRA values reflect the relative rank order between interventions and do not directly indicate the magnitude of effect sizes or the clinical importance of differences. The fact that an intervention has a higher SUCRA value means that it has a higher likelihood of being relatively superior among the interventions compared, but whether that superiority reaches a clinically meaningful threshold needs to be judged in conjunction with the effect size estimates (e.g., risk ratios, mean differences, etc.) and their 95% confidence intervals.

### Certainty of the evidence assessment

We utilized the GRADE system, as recommended by the BMJ (22), to evaluate the quality of evidence. The quality of evidence may be compromised in five ways: study limitations, inconsistency, indirectness, imprecision, and risk of bias. We used GRADE Pro version 3.6.1 to create a table outlining the levels of evidence.

## Results

### Study selection and characteristics of included trials

**Figure 1:**
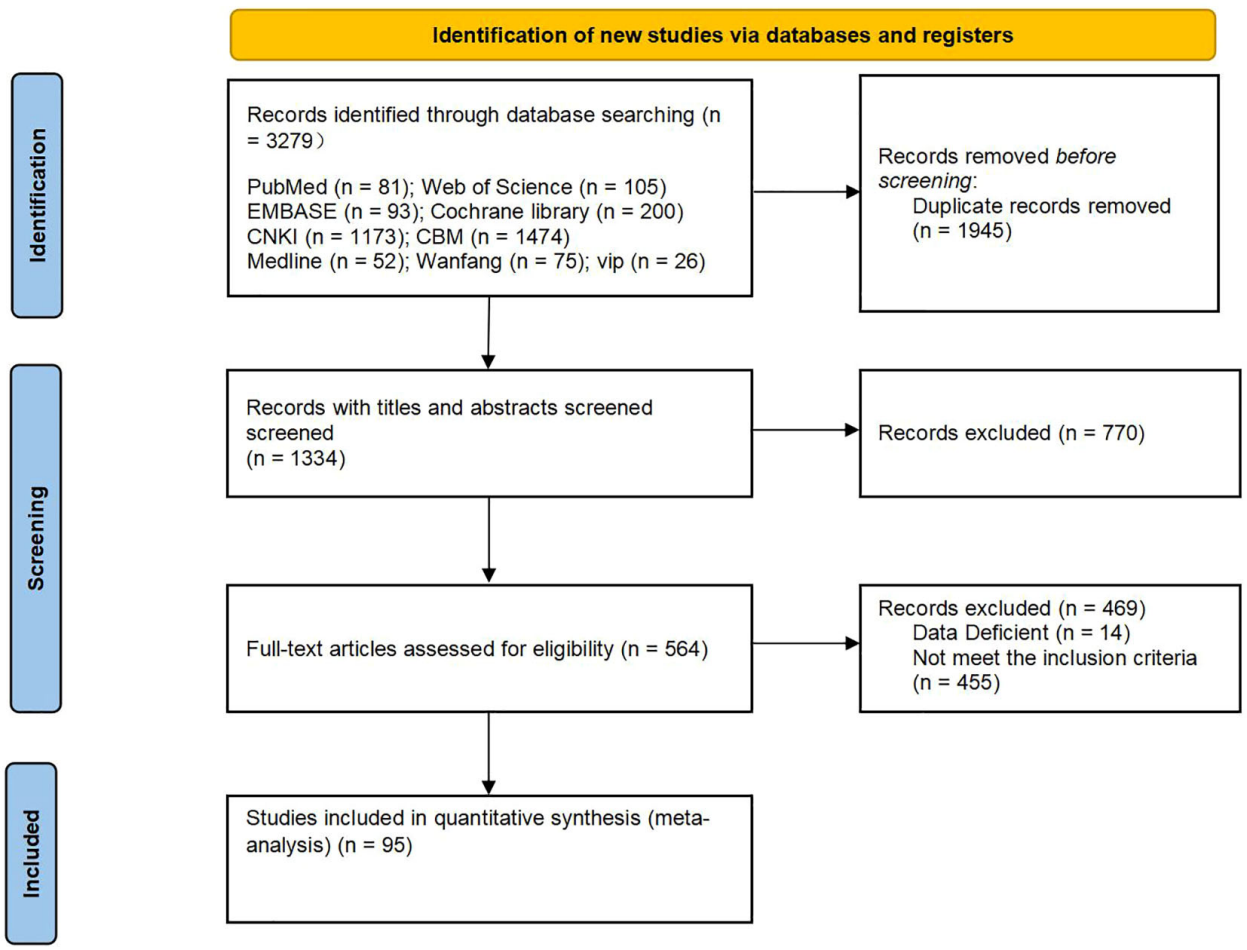
Flowchart of literature screening.

A total of 3279 studies were retrieved through the predefined search strategies, of which 1945 were duplicates. After the duplicates were eliminated, the remaining 1334 studies were browsed for titles and abstracts, and 770 were excluded in this step. Finally, the remaining 564 studies were scrutinized in full text, and 469 studies that did not meet the inclusion criteria were excluded. After the screening process, 95 RCTs were finally included; the complete screening process is shown in **Figure 1**.

The 95 RCTs incorporated within this study were all characterized by a two-arm trial design, encompassing 8,194 participants. The interventions encompassed TCM Decoction, Chinese Patent Medicine, acupuncture, electroacupuncture, TCM Decoction combined with acupuncture, TCM Decoction combined with Chinese Herbal Footbath, TCM Decoction combined with Chinese Medicine Fumigation, acupoint injections combined with Chinese Medicine Fumigation, and Western medicines, either oral or injectable. Of these RCTs, 87 (7833 participants) evaluated the total effective rate; 8 (550 participants) reported the adverse events; 87 (2461 participants) measured the motor conduction velocity of the common peroneal nerve, 23 (1893 participants) determined the sensory conduction velocity of the common peroneal nerve, 23 (2038 participants) evaluated the motor conduction velocity of the median nerve, 24 (2116 participants) assessed the sensory conduction velocity of the median nerve, 9 (817 participants) gauged the TCSS, 12 (1056 participants) scrutinized fasting blood glucose (FBG) levels, and 11 (992 participants) evaluated two-hour post-load plasma glucose (2hPG). Detailed information on the included studies is recorded in **Table 1**.

**Table 1:** Characteristics of included studies.

Author (year)	Country	Course of T2DM (T/C)	Course of DPN (T/C)	Age (T/C)	N (T/C)	Therapy (T)	Therapy (C)	Treatment duration	Outcomes
Wu (2024) (23)	China	NR	7.87 ± 1.65/7.21 ± 1.54(years)	62.21 ± 6.85/64.32 ± 5.67	70 (35/35)	TCM Decoction + Acupuncture Buyang Huanwu Decoction: *Astragalus Radix*, *Angelica*, *Paeoniae Radix Rubra*, *Earthworms*, *Ligusticum chuanxiong*, *Safflower*, *Walnut kernels*. Acupoints: GB34 and ST36.	Epalrestat	2 weeks	①②⑩
Li (2023) (24)	China	7.36 ± 2.15/7.34 ± 2.14(years)	NR	60.20 ± 8.31/60.27 ± 8.34	80 (40/40)	TCM Decoction + Acupuncture Yiqi Tongluo Decoction: *Astragalus Radix*, *Atractylodes lancea*, *Trichosanthes kirilowii Maxim*, *Rehmannia glutinosa*, *Salvia miltiorrhiza*, *Angelica*, *Dioscoreae Rhizoma*, *Bombyx batryticatus*, Leech, *centipedes*, *Panax quinquefolius*. Acupoints: LI11, ST36, LI4, LR3, SJ5.	Mecobalamin	3 weeks	④⑤⑥⑦⑧
Liu (2022) (25)	China	NR	5.49 ± 2.92/4.13 ± 1.91 (years)	56.00 ± 5.75/55.67 ± 5.50	60 (30/30)	TCM Decoction+ Acupuncture Wengyang Jianbu Decoction: *Ephedrae herba*, *Aconiti Lateralis Radix Praeparata*, *Asarum, Radix Paeoniae Albab*, *Salvia miltiorrhiza*, *Dendrobium*, *Achyranthes bidentata*.	Pregabalin	22 weeks	④⑤⑥⑦⑧
Ding (2022) (26)	China	11.20 ± .84/11.43 ± 9.37 (years)	NR	61.54 ± 8.35/61.94 ± 11.36	65 (32/33)	Chinese Patent Medicine Compound Qiying Granule: *Astragalus Radix*, *Salvia milti orrhiza*, *Cicadae Periostracum*, *Polygonatum*, *Chickpea*.	Epalrestat	12 weeks	①③④⑤⑥⑦⑧
Cui (2022) (27)	China	11.20 ± 3.93/11.47 ± 5.51 (years)	2.21 ± 0.29/2.34 ± 0.35 (years)	58.94 ± 11.08/60.14 ± 12.51	120 (61/59)	TCM Decoction Huangqi Guizhi Wuwu Decoction: *Astragalus Radix*, *Radix Paeoniae Albab*, *Rimulus Cinnamomi*, Ginger, *Chinese jujube*.	Mecobalamin	12 weeks	①②③④⑤⑥⑦⑧
Ma (2022) (28)	China	NR	7.0 ± 1.7/7.0 ± 2.0 (years)	54.5 ± 6.2/53.5 ± 6.2	96 (48/48)	TCM Decoction Yiqi Qingre Tongmai Decoction: *Astragalus Radix*, *Angelica*, *Spatholobus suberectus*, *Taxillus chinensis*, *Flos Lonicerae Japonicae*, *Paeoniae Radix Rubra*, *Ligusticum Chuanxiong*, *Safflower*, *Prunus davidiana*, *Earthworms*.	Epalrestat	4 weeks	①②③④⑤⑥⑦⑨⑩
Zhao (2022) (29)	China	NR	8.24 ± 1.24/8.63 ± 1.35 (years)	59.34 ± 2.63/60.17 ± 2.23	93 (47/46)	TCM Decoction Shenqi pills combined with Danggui Sini Decoction: *Rehmannia glutinosa*, *Dioscoreae Rhizoma*, *Cornus officinali, Alismatis Rhizoma*, *Poria cocos*, *Moutan Cortex*, *Rimulus Cinnamomi*, *Angelica*, *Aconiti Lateralis Radix Praeparata*, *Radix Paeoniae Albab, Asarum*, *Licorice*, *Tetrapanax papyrifer*, *Ziziphus jujuba*.	Mecobalamin	12 weeks	⑧
Han (2021) (30)	China	NR	NR	54.5 ± 3.8/54.8 ± 3.5	82 (41/41)	TCM Decoction+ Chinese Medicine Fumigation Danggui Sini Decoction: *Tetrapanax papyrifer*, Asarum, *Licorice*, *Radix Paeoniae Albab*, *Astragalus Radix*, *Rimulus Cinnamomi*, *Spatholobus suberectus*, *Angelica*, *Ziziphus jujuba*, *Achyranthes bidentata*.	Mecobalamin	4 weeks	⑧
Wang (2021) (31)	China	11.37 ± 4.91/10.88 ± 3.56 (years)	3.44 ± 0.65/3.52 ± 0.77 (years)	61.72 ± 7.69/60.18 ± 5.41	120 (60/60)	TCM Decoction+ Acupuncture Chinese medicinal formulae: *Astragalus Radix*, *Rimulus Cinnamomi*, *Radix Paeoniae Albab*, *Spatholobus suberectus*, *Angelica*, Ginger, *Corydalis yanhusuo*. Acupoints: SP10, ST36, SP6, LI10, LI11, BL17.	Mecobalamin	8 weeks	①②③④⑤⑥⑦⑧⑩
Jin (2021) (32)	China	NR	145.67 ± 70.68/173.48 ± 84.97 (months)	62.23 ± 7.32/64.36 ± 7.08	104 (53/51)	Chinese Patent Medicine Shenxie Zhitong Capsule: *Panax notoginseng*, Scorpions, *Eupolyphaga steleophaga*, *Eupolyphaga steleophaga*.	Lipoic acid Tablets	12 weeks	①②③⑩
Hu (2021) (33)	China	NR	2.48 ± 1.01/2.42 ± 0.95 (years)	62.52 ± 4.35/62.45 ± 4.30	66 (33/33)	TCM Decoction Huangqi Guizhi Wuwu Decoction: *Ziziphus jujuba*, *Licorice*, Ginger, *Radix Paeoniae Albab*, *Safflower*, *Salvia miltiorrhiza*, *Astragalus Radix*, *Spatholobus suberectus*, *Rimulus Cinnamomi*.	Mecobalamin	8 weeks	④⑤⑥⑦⑧⑨
Yang (2021) (34)	China	NR	103.6 ± 46.9/108.4 ± 47.5 (months)	60.5 ± 10.3/60.2 ± 10.5	49 (25/24)	TCM Decoction Yiqi Huoxue Tongbi Decoction: *Astragalus Radix*, *Dictamnus dasycarpus*, *Rimulus Cinnamomi*, *Spatholobus suberectus*, *Ligustrum lucidum Ait*, *Eclipta prostrata (L.)*, *Paeoniae Radix Rubra*, *Angelica*, mulberry, *Safflower*, *Prunus davidiana*, Scorpions, *Earthworms*	Mecobalamin	8 weeks	③④⑤⑥⑦⑧⑨
Bai (2021) (35)	China	NR	4.31 ± 1.42/3.21 ± 1.03 (years)	65.54 ± 2.93/65.52 ± 2.81	61 (31/30)	TCM Decoction. Shengmai San formula: ginseng, *Ophiopogon japonicus*, *Schisandra chinensis*, *Woodwardia japonica*, *Dipsacus*, *Euonymus alatus*, *Cornus officinali*, *Astragalus Radix*, *Salvia miltiorrhiza*.	Mecobalamin	8 weeks	⑧
Wu (2021) (36)	China	14.5 ± 2.3/14.8 ± 2.4 (years)	1.3 ± 0.5/1.3 ± 0.5 (years)	51.8 ± 4.3/52.5 ± 4.2	120 (60/60)	TCM Decoction Tangshen’an Decoction: *Astragalus Radix*, *Mulberry twig*, *Pueraria montana*, *Salvia miltiorrhiza*, *Achyranthes bidentata*, Trogopterus Feces, Trogopterus Feces, Trogopterus Feces, *Rimulus Cinnamomi*, *Ptyas dhumnades*, *Panax notoginseng*, Scorpions.	Mecobalamin	4 weeks	④⑥⑦
Li (2021) (37)	China	NR	6.08 ± 1.35/6.13 ± 1.63 (years)	61.67 ± 5.82/61.59 ± 5.25	100 (50/50)	TCM Decoction Erxian Dihuang Decoction: *Astragalus Radix*, *Clematis chinensis* Osbeck, *Clematis chinensis* Osbeck, *Dioscoreae Rhizoma*, *Ligusticum Chuanxiong*, *Angelica*, *Cornus officinali*, *Spatholobus suberectus*, *Rehmannia glutinosa*, *Moutan Cortex*, *Poria cocos*, *Panax quinquefolius*.	Mecobalamin	4 weeks	⑥⑦⑧
Jiang (2021) (38)	China	NR	NR	59.0 ± 2.1/58.6 ± 2.8	92 (46/46)	TCM Decoction Chinese medicinal formulae: *Angelica*, *Rimulus Cinnamomi*, *Tetrapanax papyrifer*, *Radix Paeoniae Albab*, *Licorice*, *Asarum*, *Ziziphus jujuba*.	Mecobalamin Alprostadil Injection	12 weeks	①②③⑧
Liu (2020) (39)	China	NR	8.22 ± 2.76/8.37 ± 2.81	56.33 ± 4.28/55.28 ± 4.19	70 (35/35)	TCM Decoction+ Chinese Medicine Fumigation Chinese medicinal formulae: *Spatholobus suberectus*, *Radix Paeoniae Albab*, *Angelica*, *Astragalus Radix*, *Earthworms*, *Rimulus Cinnamomi*, *Licorice*, *Ziziphus jujuba*, *Tetrapanax papyrifer*.	Mecobalamin	4 weeks	⑧
Dai ^(2)^ (2020) (40)	China	NR	6.08 ± 3.01/6.12 ± 2.56 (years)	54.92 ± 7.51/54.73 ± 8.46	170 (85/85)	TCM Decoction+ Chinese Herbal Footbath Chinese medicinal formulae: *Salvia miltiorrhiza*, *Astragalus Radix*, *Radix Paeoniae Albab*, *Dendrobium*, *Rimulus Cinnamomi*, *Angelica*, *Spatholobus suberectus*, *Paeoniae Radix Rubra*c, *Ligusticum Chuanxiong*, *Earthworms*, *Ziziphus jujuba*, Ginger.	Mecobalamin	4 weeks	④⑤⑥⑦⑧
Chen (2020) (41)	China	NR	3.4 ± 1.4/3.6 ± 1.3 (years)	60.4 ± 4.3/60.3 ± 4.4	104 (52/52)	TCM Decoction+ Chinese Herbal Footbath Chinese medicinal formulae: *Salvia miltiorrhiza*, *Astragalus Radix*, *Radix Paeoniae Albab*, *Dendrobium*, *Rimulus Cinnamomi*, *Angelica*, *Spatholobus suberectus*, *Paeoniae Radix Rubra*c, *Ligusticum Chuanxiong*, *Earthworms*, *Ziziphus jujuba*, Ginger, *Cyathula officinalis* Kuan.	Mecobalamin	2 weeks	⑧
Cheng ^(2)^ (2020) (42)	China	NR	7.75 ± 1.63/7.91 ± 1.48 (years)	63.28 ± 4.47/62.74 ± 4.12	62 (31/31)	TCM Decoction+ Acupuncture Jiawe Huangqi Guizhi Wuwu Decoction: *Astragalus Radix*, *Radix Paeoniae Albab*, *Rimulus Cinnamomi*, *Angelica*, *Spatholobus suberectus*, *Taxillus chinensis*, *Ziziphus jujuba*, *Ligustrum lucidum Ait*, *Coptis chinensis*, *Pseudocydonia sinensis*. Acupoints: SP10, ST36, SP6, LI11, BL23, BL20, BL18, PC6, BL40, RN6, GB34, EX_UE9, EX.	Vitamin B1 Mecobalamin	12 weeks	⑧
Guo (2020) (43)	China	NR	5.38 ± 1.73/5.46 ± 1.67	47.32 ± 2.79/46.27 ± 2.19	100 (50/50)	Electroacupuncture Acupoints: SP10, SP6, LI11, LI4, RN6, RN4, ST40.	α-lipoic acid	4 weeks	④⑤⑥⑦⑨
Lin (2020) (44)	China	NR	6.3 ± 3.5/7.2 ± 3.0 (years)	47.2 ± 8.0/47.8 ± 9.3	120 (60/60)	Chinese Patent Medicine Compound Xueshuantong Capsules: *Salvia miltiorrhiza*, *Astragalus Radix*, *Panax notoginseng*, *Scrophularia striata*.	Mecobalamin	12 weeks	④⑤⑥⑦⑧⑩
Cheng (2020) (45)	China	NR	11.40 ± 1.05/11.32 ± 1.02 (weeks)	56.46 ± 2.56/56.32 ± 2.55	80 (40/40)	TCM Decoction Jiawei Huangqi Guizhi Wuwu Decoction: *Salvia miltiorrhiza*, *Astragalus Radix*, *Rimulus Cinnamomi*, *Angelica*, *Paeoniae Radix Rubra*, *Ligusticum Chuanxiong*, *Earthworms*, *Ziziphus jujuba*, Ginger.	Vitamin B1 Vitamin B6	4 weeks	⑧
Song (2020) (46)	China	11.7 ± 4.2/12.3 ± 5.1 (years)	NR	54.3 ± 12.6/55.1 ± 14.8	60 (30/30)	TCM Decoction Danggui Niantong Decoction: *Rhizoma et Radix Notopterygii*, *Rhizoma et Radix Notopterygii*, *Saposhnikovia divaricata*, *Cimicifuga Rhizoma*, Polyporus, *Angelica*, *Artemisia capillaris*, *Alismatis Rhizoma*, *Pueraria montana*, *Scutellaria baicalensis*, *Radix Paeoniae Albab*, *Atractylodes macrocephala*, *Sophora flavescens*, *Atractylodes lancea*, *Asarum*, *Licorice*, *Corydalis yanhusuo*.	Mecobalamin	4 weeks	①②③④⑤⑥⑦⑧
Zhi (2020) (47)	China	7.45 ± 2.15/8.35 ± 3.24 (years)	NR	61.25 ± 4.34/62.46 ± 4.53	80 (40/40)	TCM Decoction Huoluo Zhixiao formula: *Salvia miltiorrhiza*, *Pseudocydonia sinensis*, *Achyranthes bidentata*, *Ligusticum Chuanxiong*, *Licorice*, *Eupolyphaga steleophaga*, *Woodwardia japonica*, *Dipsacus*, *Euonymus alatus*, Leech.	Mecobalamin	20 weeks	①②③④⑤⑥⑦⑧
Dai (2020) (48)	China	6.31 ± 3.27/6.28 ± 3.18 (years)	4.67 ± 2.53/4.58 ± 2.72 (years)	52.22 ± 3.63/52.13 ± 3.52	106 (53/53)	TCM Decoction Yiqi Huayu Tongbi formula: *Pueraria montana*, *Astragalus Radix*, *Pseudocydonia sinensis*, *Radix Paeoniae Albab*, *Achyranthes bidentata*, *Polygonatum*, *Rimulus Cinnamomi*, *Angelica*, *Spatholobus suberectus*, Leech, *Angelicae Pubescentis Radix*, *Lycopodium jaPonicum Thunb*, *Paeoniae Radix Rubra*, *Pseudostellaria heterophylla*, *Ligusticum Chuanxiong*, *Safflower*, *Prunus davidiana*, *Earthworms*, *Corydalis yanhusuo*, *Clematis Radix Tt Rhizoma*, *Dandelion.*	Mecobalamin	12 weeks	④⑥⑦
Shi (2020) (49)	China	7.5 ± 3.7/73 ± 3.6 (years)	NR	56.21/55.1	80 (40/40)	TCM Decoction Yiqi Huoxue Tongbi Decoction: *Astragalus Radix*, *Rimulus Cinnamomi*, *Spatholobus suberectus*, *Paeoniae Radix Rubra*, *Safflower*, *Uncaria rhynchophylla*, *Prunus davidiana*Scorpions, Mulberry, *Eclipta Prostrata L.*	Mecobalamin VitaminE1	4 weeks	⑥⑦⑧
Pang (2020) (50)	China	NR	3.72 ± 1.18/3.69 ± 1.13 (years)	57.16 ± 8.30/56.75 ± 8.24	90 (45/45)	TCM Decoction Chuanwu Tongluo formula: *Astragalus Radix*, *Radix Paeoniae Albab*, *Rimulus Cinnamomi*, *Angelica*, Leech, *Radix Aconiti* Preparata, *Rehmannia glutinosa*, *Licorice*.	α-lipoic acid	3 weeks	⑧
Jian (2020) (51)	China	10.12 ± 1.32/10.66 ± 1.32 (years)	NR	61.51 ± 1.02/62.22 ± 1.65	100 (50/50)	TCM Decoction Shuanghe Decoction: *Radix Paeoniae Albab*, *Angelica*, *Ligusticum Chuanxiong*, *Safflower*, *Prunus davidiana*, *Radix Rehmanniae*, *Pinallia*, *Poria cocos*, *Licorice*, *Critri Reticulatae Pericarpium*.	Mecobalamin	12 weeks	⑧⑩
Zhang (2019) (52)	China	NR	NR	53.22 ± 8.32/53.13 ± 8.24	70 (35/35)	TCM Decoction Huangqi Guizhi Wuwu Decoction: *Astragalus Radix*, *Radix Paeoniae Albab*, *Rimulus Cinnamomi*, *Ziziphus jujuba*, Ginger.	Mecobalamin	4 weeks	⑧
Zhang ^(2)^ (2019) (53)	China	NR	11.69 ± 4.93/11.88 ± 5.00 (years)	52.91 ± 8.47/52.84 ± 8.04	64 (32/32)	TCM Decoction+ Chinese Herbal Footbath Chinese medicinal formulae: *Pseudocydonia sinensis*, *Radix Paeoniae Albab*, *Achyranthes bidentata*, *Angelica*, *Paeoniae Radix Rubra*, *Ligusticum Chuanxiong*, *Safflower*, *Prunus davidiana*, *Radix Rehmanniae*, *Earthworms*, *Licorice*.	Mecobalamin	8 weeks	⑧⑩
Deng (2019) (54)	China	NR	4.6 ± 1.1/5.1 ± 0.8 (years)	48.6 ± 2.7/50.2 ± 2.5	90 (45/45)	TCM Decoction+ Chinese Herbal Footbath Chinese medicinal formulae: *Salvia miltiorrhiza*, *Astragalus Radix*, *Radix Paeoniae Albab*, *Dendrobium*, *Cyathula*, *Rimulus Cinnamomi*, *Angelica*, *Spatholobus suberectus*, Leech, *Paeoniae Radix Rubra*, *Ligusticum Chuanxiong*, *Earthworms*, *Ziziphus jujuba*, Ginger.	Mecobalamin	4 weeks	⑧
Meng (2019) (55)	China	NR	3.3 ± 2.7/3.67 ± 2.95 (years)	NR	99 (50/49)	TCM Decoction+ Chinese Herbal Footbath Huangqi Guizhi Wuwu Decoction combined Danshen Yin: *Salvia miltiorrhiza*, *Astragalus Radix*, *Radix Paeoniae Albab*, *Rimulus Cinnamomi*, *Angelica*, *Anemarrhena asphodeloides*, *Paeoniae Radix Rubra*, *Rehmannia glutinosa*, *Dioscoreae Rhizoma*, *Licorice*.	Mecobalamin	4 weeks	④⑤⑧
Chai (2019) (56)	China	NR	7.8 ± 2.1/7.9 ± 2.3 (years)	65.8 ± 3.6/66.0 ± 3.5	80 (40/40)	TCM Decoction+ Acupuncture Huangqi Guizhi Wuwu Decoction: *Salvia miltiorrhiza*, *Astragalus Radix*, *Radix Paeoniae Albab*, *Rimulus Cinnamomi*, *Angelica*, *Spatholobus suberectus*, *Licorice*, *Ziziphus jujuba*, Ginger. Acupoints: ST36, SP6, LI11, BL23, BL20, RN4.	Mecobalamin VitaminE1	4 weeks	⑧
Guo (2019) (57)	China	NR	NR	56.05 ± 0.44/55.54 ± 0.64	98 (49/49)	Chinese Patent Medicine Compound Danshen Dripping Pills: *Salvia miltiorrhiza*, *Panax notoginseng*, *Dryobalanops aromatica*.	VitaminE1	4 weeks	⑧⑨
Song (2019) (58)	China	NR	NR	53.22 ± 8.32/53.13 ± 8.24	70 (35/35)	TCM Decoction Chinese medicinal formulae: *Scrophularia striata*, *Angelica*, *Spatholobus suberectus*, *Atractylodes lancea*,	Mecobalamin	4 weeks	⑧
Shi (2019) (59)	China	9.51 ± 1.23/9.51 ± 1.02 (years)	3 months-11 years/2 months-11 years	61.15 ± 4.22/61.23 ± 4.52	90 (45/45)	TCM Decoction Huangqi Danshen Tongluo formula: Chinese medicinal formulae: *Salvia miltiorrhiza*, *Astragalus Radix*, *Rimulus Cinnamomi*, *Angelicactus*, *Earthworms*.	Mecobalamin α-lipoic acid	4 weeks	⑧
Xu (2019) (60)	China	15.7/16.3 (years)	NR	51.6/50.9	60 (30/30)	TCM Decoction Jiawei Huangqi Guizhi Wuwu Decoction: *Salvia miltiorrhiza*, *Astragalus Radix*, *Rimulus Cinnamomi*, *Angelica*, *Paeoniae Radix Rubra*, *Ligusticum Chuanxiong*, *Earthworms*, *Ziziphus jujuba*, Ginger.	Mecobalamin Epalrestat	4 weeks	⑧
Dai (2019) (61)	China	NR	5.3 ± 1.3/5.4 ± 1.1 (years)	60.8 ± 7.5/61.2 ± 7.3	70 (35/35)	TCM Decoction Jiawei Buyang Huanwu Decoction: *Astragalus Radix*, *Pseudocydonia sinensis*, *Clematis chinensis* Osbeck, *Angelica*, *Paeoniae Radix Rubra*, *Ligusticum Chuanxiong*, *Safflower*, *Earthworms*, *Curcumae Radix*, Bamboo Shaving.	Vitamin B1 Vitamin B12	12 weeks	⑧
Zhao (2019) (62)	China	12.21 ± 6.44/12.54 ± 6.41 (years)	NR	64.58 ± 7.95/65.12 ± 8.21	100 (50/50)	TCM Decoction Danggui Sini Decoction: *Astragalus Radix*, *Radix Paeoniae Albab*, *Rimulus Cinnamomi* *Angelica*, *Spatholobus suberectus*, *Earthworms*, *Asarum*, *Licorice*, *Tetrapanax papyrifer*, *Ziziphus jujuba*.	Mecobalamin	4 weeks	⑧
Hu (2019) (63)	China	NR	NR	68.21 ± 1.32/68.92 ± 1.74	90 (45/45)	TCM Decoction Huangqi Guizhi Wuwu Decoction: *Astragalus Radix*, *Radix Paeoniae Albab*, *Rimulus Cinnamomi*, *Ziziphus jujuba*, Ginger.	Mecobalamin	4 weeks	①②
Fan (2019) (64)	China	7.31 ± 2.17/7.21 ± 2.10 (years)	NR	60.33 ± 4.55/60.24 ± 4.44	90 (45/45)	TCM Decoction Jiawei Huangqi Guizhi Wuwu Decoction: *Salvia miltiorrhiza*, *Astragalus Radix*, *Rimulus Cinnamomi*, *Angelica*, *Paeoniae Radix Rubra*, *Ligusticum Chuanxiong*, *Earthworms*, *Ziziphus jujuba*, Ginger.	Mecobalamin	6 weeks	⑧⑩
Yang (2018) (65)	China	6.95 ± 1.25/6.87 ± 1.21 (years)	3.32 ± 0.79/3.29 ± 0.78 (years)	54.43 ± 10.38/53.39 ± 10.21	80 (40/40)	TCM Decoction+ Chinese Medicine Fumigation Danggui Sini Decoction: *Astragalus Radix*, *Radix Paeoniae Albab*, *Rimulus Cinnamomi*, *Angelica*, *Asarum*, *Licorice*, *Tetrapanax papyrifer*, *Ziziphus jujuba*, Ginger.	Mecobalamin	4 weeks	⑧
Fan (2018) (66)	China	NR	7.5 ± 1.4/7.4 ± 1.6 (years)	54.6 ± 5.8/54.3 ± 6.4	400 (260/140)	TCM Decoction+ Chinese Herbal Footbath Buyang Huanwu Decoction: *Astragalus Radix*, *Angelica*, *Spatholobus suberectus*, *Paeoniae Radix Rubra*, *Ligusticum Chuanxiong*, *Safflower*, *Radix Rehmanniae*, *Earthworms*, *Licorice*, *Ophiopogon japonicus*.	Mecobalamin Vitamin B1	4 weeks	⑧
Yu (2018) (67)	China	5.8 ± 1.6/5.9 ± 1.7 (years)	NR	60.7 ± 5.2/60.8 ± 5.3	70 (36/34)	TCM Decoction+ Acupuncture Mateng Decoction: *Astragalus Radix*, *Rimulus Cinnamomi*, *Angelica*, *Paeoniae Radix Rubra*, *Ligusticum Chuanxiong*, *Rehmannia glutinosa*, *Corydalis yanhusuo*, *Panax notoginseng*, Scorpions, *Lycium barbarum*. Acupoints: ST36, SP6, LI4, BL23, BL18, PC6, GB34.	Mecobalamin Fursultiamine	4 weeks	①②⑧
Xiao (2018) (68)	China	NR	3.4 ± 1.2/3.1 ± 1.0 (years)	59.3 ± 6.1/57.5 ± 6.2	132 (66/66)	Chinese Patent Medicine Tongxinluo capsules: *Cicadae Periostracum*, *Moutan Cortex*, *Eupolyphaga steleophaga*.	Mecobalamin	8 weeks	①②③④⑤⑧
Dai (2018) (69)	China	NR	NR	NR	80 (40/40)	TCM Decoction Huangqi Guizhi Wuwu Decoction combined with Yunvjian Decoction: *Astragalus Radix*, *Radix Paeoniae Albab*, *Achyranthes bidentata*, *Rimulus Cinnamomi*, *Angelica*, *Safflower*, *Prunus davidiana*, *Rehmannia glutinosa*, *Ziziphus jujuba*, Ginger, *Ophiopogon japonicus*, *Gypsum fibrosum*.	Epalrestat	3 weeks	④⑤⑧
Sun (2018) (70)	China	2-12/3-13 (years)	NR	64.7/63.9	80 (40/40)	TCM Decoction Buyang Huanwu Decoction combined with Guizhi Fuling Pill: *Astragalus Radix*, *Radix Paeoniae Albab*, *Rimulus Cinnamomi*, *Angelica*, *Paeoniae Radix Rubra*, *Ligusticum Chuanxiong*, *Safflower*, *Prunus davidiana*, *Earthworms*, *Poria cocos*, *Moutan Cortex*.	Mecobalamin Vitamin B1	12 weeks	⑧
Cao (2018) (71)	China	NR	NR	56.8 ± 4.7/56.5 ± 4.4	71 (36/35)	TCM Decoction Danggui Sini Decoction: *Astragalus Radix*, *Radix Paeoniae Albab*, *Rimulus Cinnamomi* *Angelica*, *Spatholobus suberectus*, *Earthworms*, *Asarum*, *Licorice*, *Tetrapanax papyrifer*, *Ziziphus jujuba*.	Mecobalamin	4 weeks	⑧
Zhang (2017) (72)	China	6.8 ± 1.5/7.2 ± 1.7	NR	47.7 ± 2.14/49.5 ± 2.05	160 (85/75)	Acupoint Injection+ Chinese Medicine Fumigation Chinese medicinal formulae: Trogopterus Feces, Trogopterus Feces, *Safflower*, *Prunus davidiana*, *Eupolyphaga steleophaga*, *Radix Aconiti* Preparata, *Radix Aconiti Kusnezoffi Preparata*. Acupoints: ST36, SP6, GB34.	Mecobalamin	2 weeks	⑧
Chen (2017) (73)	China	10.79 ± 4.77/10.25 ± 4.08	NR	43.52 ± 5.26/43.21 ± 5.62	66 (33/33)	Acupoint Injection+ Chinese Medicine Fumigation Chinese medicinal formulae: *Rhizoma et Radix Notopterygii*, *Rimulus Cinnamomi*, *Angelica*, *Clematis Radix Tt Rhizoma*, *Safflower*, *Asarum*, *Aconitum carmichaelii Debx.* Acupoints: SP10, ST36, GB34.	Mecobalamin	12 weeks	⑧⑨
Wang (2017) (74)	China	8.4 ± 1.5/9.1 ± 1.2 (years)	3.1 ± 0.7/3.4 ± 0.6 (years)	57.6 ± 4.5/58.1 ± 4.2	74 (37/37)	TCM Decoction Danggui Sini Decoction: *Astragalus Radix*, *Radix Paeoniae Albab*, *Rimulus Cinnamomi* *Angelica*, *Spatholobus suberectus*, *Earthworms*, *Asarum*, *Licorice*, *Tetrapanax papyrifer*, *Ziziphus jujuba*.	Mecobalamin	4 weeks	⑧⑩
Zhang ^(2)^ (2017) (75)	China	NR	NR	55.01 ± 4.51/55.07 ± 4.46	60 (30/30)	TCM Decoction Tongmai Decoction: *Salvia miltiorrhiza*, *Mulberry twig*, *Astragalus Radix*, *Pseudocydonia sinensis*, *Radix Paeoniae Albab*, *Spatholobus suberectus*, *Ligusticum Chuanxiong*, *Earthworms*, *Bombyx Batryticatus*.	Mecobalamin Vitamin B1	4 weeks	⑧
Gong (2017) (76)	China	NR	6.12 ± 1.37 (years)	60.12 ± 3.18	120 (60/60)	TCM Decoction Buyang Huanwu Decoction: *Astragalus Radix*, *Radix Paeoniae Albab*, *Achyranthes bidentata*, *Angelica*, *Spatholobus suberectus*, *Paeoniae Radix Rubra*, *Ligusticum Chuanxiong*, *Safflower*, *Earthworms*, *Dioscoreae Rhizoma*, *Poria cocos*, *Licorice*, *Lycopodium clavatum*.	Mecobalamin	8 weeks	⑧
Zhang ^(3)^ (2017) (77)	China	14.3 ± 1.3/15.2 ± 2.1 (years)	13.7 ± 1.7/13.6 ± 1.4 (years)	63.5 ± 2.5/64.2 ± 1.9	68 (34/34) (	TCM Decoction Yiqi Huoxue Tongbi Decoction: *Astragalus Radix*, *Rimulus Cinnamomi*, *Spatholobus suberectus*, *Paeoniae Radix Rubra*, *Safflower*, *Uncaria rhynchophylla*, *Prunus davidiana*Scorpions, Mulberry, *Eclipta Prostrata L.*	TCM Decoction	4 weeks	⑧
Cheng (2017) (78)	China	3.84 ± 1.23/3.45 ± 1.09 (years)	NR	53.88 ± 7.71/52.48 ± 8.58	84 (42/42)	TCM Decoction Yiqi Huoxue Tongbi Decoction: *Astragalus Radix*, *Rimulus Cinnamomi*, *Spatholobus suberectus*, *Paeoniae Radix Rubra* *Safflower*, *Uncaria rhynchophylla*, *Prunus davidiana*Scorpions, Mulberry, *Eclipta Prostrata L.*	Mecobalamin Vitamin E1	4 weeks	⑧⑨
Deng (2017) (79)	China	NR	2.3 ± 0.7/2.1 ± 0.8 (years)	58.37 ± 6.53/58.42 ± 6.4	90 (45/45)	TCM Decoction Qigui Tangtongning formula: *Pueraria montana*, *Astragalus Radix*, *Angelica*, *Spatholobus suberectus*, *Clematis Radix Tt Rhizoma*, *Radix Rehmanniae*, *Corydalis yanhusuo*.	Mecobalamin	12 weeks	⑧
Liu (2017) (80)	China	NR	NR	53.6 ± 9.4/53.2 ± 9.3	72 (36/36)	TCM Decoction Yiqi Huoxue Tongbi Decoction: *Astragalus Radix*, *Rimulus Cinnamomi*, *Spatholobus suberectus*, *Paeoniae Radix Rubra* *Safflower*, *Uncaria rhynchophylla*, *Prunus davidiana*Scorpions, Mulberry, *Eclipta Prostrata L.*	Mecobalamin	4 weeks	⑧
Chou (2017) (81)	China	NR	6.8 ± 2.5/6.5 ± 2.5 (years)	42.5 ± 5.8/42.7 ± 5.6	100 (50/50)	TCM Decoction Yiqi Huayu Tongbi formula: *Pueraria montana*, *Astragalus Radix*, *Clematis chinensis*, *Angelica*, *Spatholobus suberectus* Leech, *Ligusticum Chuanxiong*, *Clematis Radix Tt Rhizoma*, *Eupolyphaga steleophaga*, *Pseudostellaria heterophylla*.	Mecobalamin	4 weeks	⑧
Xu (2017) (82)	China	NR	NR	50.27 ± 6.21/48.68 ± 5.89	70 (35/35)	TCM Decoction Danggui Sini Decoction: *Astragalus Radix*, *Radix Paeoniae Albab*, *Rimulus Cinnamomi* *Angelica*, *Spatholobus suberectus*, *Earthworms*, *Asarum*, *Licorice*, *Tetrapanax papyrifer*, *Ziziphus jujuba*.	Vitamin B1 and Vitamin B2	2 weeks	④⑤⑧
Dong (2017) (83)	China	14.8 ± 8.34/15.03 ± 6.75 (years)	8.30 ± 6.3/8.33 ± 5.11 (years)	57.8 ± 8.43/58.5 ± 9.20	80 (40/40)	TCM Decoction Fuyang Tongluo Decoction: *Angelica*, *Atractylodes macrocephala*, *Aconiti Lateralis Radix Praeparata*, *Asarum* *Licorice*, *Caulis Lonicerae*.	Mecobalamin	8 weeks	⑧
Zhang (2016) (84)	China	4.3-20.0/4.4-21.0 (years)	NR	50.72 ± 12.35/52.91 ± 12.16	90 (45/45)	Acupoint Injection+ Chinese Medicine Fumigation Chinese medicinal formulae: *Rhizoma et Radix Notopterygii* *Rimulus Cinnamomi*, *Angelicae Pubescentis Radix*, *Clematis Radix Tt Rhizoma*, *Safflower*, *Prunus davidiana*, *Asarum*, *Artemisia argyi Folium*, *Aconitum carmichaelii Debx.*	Mecobalamin	12 weeks	⑧
Pan (2016) (85)	China	NR	8.86 ± 3.12/9.12 ± 2.12 (years)	46.52 ± 7.14/45.78 ± 7.82	64 (32/32)	TCM Decoction+ Acupuncture Huangqi Xiaoke formula: *Salvia miltiorrhiza*, *Astragalus Radix*, *Dendrobium*, *Scrophularia striata*, *Codonopsis Pilosula*, *Trichosanthis radix*, Litchi seed, *Leonurus japonicu Ligustrum lucidum Ait.*s Acupoints: ST36, SP6, LI11, LI4, BL17, RN6, GB34, RN4, KI3.	Mecobalamin	4 weeks	⑧
Lu (2016) (86)	China	NR	3.6 ± 1.3/3.5 ± 1.1 (years)	66.0 ± 7.0/64.0 ± 7.0	60 (31/29)	Acupuncture Acupoints: ST36, BL17, BL23, BL20, BL18, KI3, EX_B3, Ashi Point.	Lipoic acid Prostaglandin E1	4 weeks	④⑤⑥⑦⑧
Zhao ^(2)^ (2016) (87)	China	NR	7.0 ± 3.0 (months)	53 ± 9.2	60 (30/30)	Acupuncture Acupoints: KI6, BL62, SJ5, LU7, SP4, GB41, SI3, PC6.	Mecobalamin Nimodipine	8 weeks	④⑤⑧
Lin (2016) (88)	China	NR	3.12 ± 1.23/3.15 ± 1.98 (years)	59.27 ± 5.41/59.16 ± 5.32	70 (35/35)	Acupuncture Acupoints: ST36, SP6, PC6, RN6, LU7, SP4, KI4, HT5, LR5.	Mecobalamin	4 weeks	③④⑤⑥⑦⑧
Zhao (2016) (89)	China	NR	9.0 ± 0.82/10 ± 0.63 (years)	53.0 ± 0.95/52.0 ± 1.69	72 (36/36)	TCM Decoction Yiqi Huoxue Tongbi Decoction: *Astragalus Radix*, *Rimulus Cinnamomi*, *Angelica*, *Spatholobus suberectus*, *Paeoniae Radix Rubra*, *Safflower*, *Uncaria rhynchophylla*, *Prunus davidiana*, Scorpions, *Ligustrum lucidum Ait.*, Mulberry, *Eclipta prostrata L.*	Mecobalamin	2 weeks	⑧
Deng (2016) (90)	China	9.51 ± 5.20/9.47 ± 5.08 (years)	NR	59.64 ± 5.99/59.71 ± 5.95	78 (39/39)	TCM Decoction Jiawei Huangqi Guizhi Wuwu Decoction: *Salvia miltiorrhiza*, *Astragalus Radix*, *Rimulus Cinnamomi*, *Angelica*, *Paeoniae Radix Rubra*, *Ligusticum Chuanxiong*, *Earthworms*, *Ziziphus jujuba*, Ginger.	Mecobalamin	8 weeks	④⑤⑥⑦⑧
Liu (2016) (91)	China	5.0-18.0/6.0-18.0 (years)	13.0 ± 7.0/12.0 ± 6.0 (years)	53.0 ± 12.2/54.0 ± 12.5	80 (40/40)	TCM Decoction Jiawei Huangqi Guizhi Wuwu Decoction: *Astragalus Radix*, *Radix Paeoniae Albab*, *Rimulus Cinnamomi*, *Atractylodes macrocephala*, *Pseudostellaria heterophylla*, *Pseudostellaria heterophylla*, *Trichosanthis radix*, Ginger.	Mecobalamin	4 weeks	⑧
Ding (2016) (92)	China	NR	1.0-7.0/1.0-8.0 (years)	54.5 ± 8.3/53.6 ± 7.8	80 (40/40)	TCM Decoction Danggui Sini Decoction: *Astragalus Radix*, *Radix Paeoniae Albab*, *Rimulus Cinnamomi* *Angelica*, *Spatholobus suberectus*, *Earthworms*, *Asarum*, *Licorice*, *Tetrapanax papyrifer*, *Ziziphus jujuba*.	Mecobalamin	4 weeks	④⑤⑧
Ma (2016) (93)	China	12.17 ± 3.21/12.45 ± 3.04 (years)	NR	56.71 ± 5.04/57.02 ± 4.99	57 (29/28)	TCM Decoction Jiawei Buyang Huanwu Decoction: *Astragalus Radix*, *Pseudocydonia sinensis*, *Clematis chinensis* Osbeck, *Angelica*, *Paeoniae Radix Rubra*, *Ligusticum Chuanxiong*, *Safflower*, *Earthworms*, *Curcumae Radix*, Bamboo Shaving.	Mecobalamin	8 weeks	⑧
Qin (2016) (94)	China	NR	NR	51.0 ± 2.5/49.0 ± 1.2	78 (39/39)	TCM Decoction Yiqi Huoxue Tongbi Decoction: *Astragalus Radix*, *Rimulus Cinnamomi*, *Angelica*, *Spatholobus suberectus*, *Paeoniae Radix Rubra*, *Safflower*, *Uncaria rhynchophylla*, *Prunus davidiana*, Scorpions, *Ligustrum lucidum Ait.*, Mulberry, *Eclipta prostrata L.*	Mecobalamin	8 weeks	⑧
Zhou (2016) (95)	China	9.6 ± 1.4 (years)	3.2 ± 1.1 (years)	55.4 ± 2.6	70 (35/35)	TCM Decoction Yiqi Huoxue Tongbi Decoction: *Astragalus Radix*, *Rimulus Cinnamomi*, *Angelica*, *Spatholobus suberectus*, *Paeoniae Radix Rubra*, *Safflower*, *Uncaria rhynchophylla*, *Prunus davidiana*, Scorpions, *Ligustrum lucidum Ait.*, Mulberry, *Eclipta prostrata L.*	Fursultiamine	8 weeks	⑧
Ma ^(2)^ (2016) (96)	China	9.4 ± 1.9 (years)	2.4 ± 0.9 (years)	65.6 ± 4.8	100 (50/50)	TCM Decoction Danggui Sini Decoction: *Astragalus Radix*, *Radix Paeoniae Albab*, *Rimulus Cinnamomi*, *Angelica*, *Spatholobus suberectus*, *Earthworms*, *Asarum*, *Licorice*, *Tetrapanax papyrifer*.	α-lipoic acid	4 weeks	⑧
Zou (2016) (97)	China	NR	6.0-22.0/4.0-24.0 (years)	45.0-79.0/46.0-78.0	98 (49/49)	TCM Decoction Buyang Huanwu Decoction: *Astragalus Radix*, *Radix Paeoniae Albab*, *Rimulus Cinnamomi*, *Angelica*, *Spatholobus suberectus*, *Paeoniae Radix Rubra*, *Ligusticum Chuanxiong* *Prunus davidiana*, *Radix Rehmanniae*, *Earthworms*, *Lycium barbarum*, *Licorice*.	Mecobalamin Vitamin B1	4 weeks	⑧
Mo (2016) (98)	China	2.0-23.0/2.0-19.0 (years)	NR	65.28 ± 9.09/62.34 ± 8.17	65 (33/32)	TCM Decoction Ynagyin Jiedu Decoction: *Salvia miltiorrhiza*, *Pueraria montana*, *Astragalus Radix*, *Scrophularia striata*, *Flos Lonicerae Japonicae*, Litchi seed, *Licorice*, *Schisandra chinensis*, *Polygonatum odoratum*.	Mecobalamin	8 weeks	⑧
Huang (2015) (99)	China	NR	6.0-20.0/5.0-19.0 (years)	40.0-72.0/41.0-79.0	120 (60/60)	TCM Decoction Danggui Sini Decoction: *Astragalus Radix*, *Radix Paeoniae Albab*, *Rimulus Cinnamomi*, *Angelica*, *Spatholobus suberectus*, *Asarum*, *Licorice*, Dried Ginger, *Caulis Polygoni Multiflor*i.	Mecobalamin	4 weeks	⑧
Zhang (2015) (100)	China	6.83 ± 3.27/6.60 ± 3.20 (years)	1.22 ± 0.80/1.37 ± 0.96 (years)	58.55 ± 7.77/57.67 ± 7.47	59 (29/30)	TCM Decoction Yanghe Decoction combined with Buyang Huanwu Decoction: *Astragalus Radix*, *Angelica*, *Paeoniae Radix Rubra*, *Ligusticum Chuanxiong*, *Safflower*, *Prunus davidiana*, *Earthworms*, *Rehmannia glutinosa*, *Licorice*, Ginger, *Rehmannia glutinosa*, *Sinapis alba L.*, Ephedrae Herba, Antler glue.	Mecobalamin	4 weeks	⑧
Xue (2015) (101)	China	NR	NR	36.0-78.0/35.0-78.0	84 (42/42)	TCM Decoction Jiawei Liuteng Shuilu Shexian Decoction: *Astragalus Radix*, *Ptyas dhumnades*, *Rimulus Cinnamomi*, *Spatholobus suberectus* Leech, *Clematis Radix Tt Rhizoma*, *Safflower*, *Uncaria rhynchophylla*, *Prunus davidiana*, *Piper kadsura*, *Caulis Sinomenii*, *Trachelospermum jasminoides*.	Mecobalamin	3 weeks	⑧
Shi (2015) (102)	China	NR	4.45 ± 1.13/4.72 ± 2.03 (years)	55.73 ± 6.13/56.83 ± 7.21	90 (45/45)	TCM Decoction Huoxue Bushen: *Salvia miltiorrhiza*, *Achyranthes bidentata*, *Angelica*, *Spatholobus suberectus*, *Paeoniae Radix Rubra*, *Ligusticum Chuanxiong*, *Safflower*, *Prunus davidiana*, *Earthworms*, *Cynomorium songaricum Rupr.*, *Eucommia ulmoides Oliver*., *Homalomena occulta*, Charred hawthorn.	Mecobalamin	8 weeks	⑧
Li (2014) (103)	China	NR	3.2 ± 1.7/3.4 ± 1.2 (years)	63.5 ± 4.2/61.8 ± 5.0	46 (23/23)	Chinese Patent Medicine Compound Danshen Dripping Pills: *Salvia miltiorrhiza*, *Panax notoginseng*, *Dryobalanops aromatica*.	Vitamin B1	12 weeks	⑧⑨
Tang (2014) (104)	China	NR	5.0-17.0/5.0-15.0 (years)	48.0-67.0/46.0-65.0	64 (32/32)	TCM Decoction Xiao Ke 5 prescription: *Astragalus Radix*, *Cyathula officinalis* Kuan, *Spatholobus suberectus*, *Safflower*, *Prunus davidiana*, *Earthworms*, *Bombyx Batryticatus*, Litchi seed.	Mecobalamin	4 weeks	④⑤⑥⑦⑧
Ye (2014) (105)	China	10.21 ± 6.24/7.48 ± 5.35 (years)	NR	61.80 ± 9.48/58.70 ± 8.89	60 (30/30)	Electroacupuncture Acupoints: ST36, LI11, LI4, KI3, LR3, KI1.	Mecobalamin	4 weeks	④⑤⑥⑦⑧
Chen (2012) (106)	China	10.8 ± 8.9/11.8 ± 4.9 (years)	3.6 ± 1.3/2.7 ± 1.2 (years)	59.3 ± 6.8/58.6 ± 8.7	84 (42/42)	TCM Decoction Buyang Huanwu Decoction: *Salvia miltiorrhiza*, *Pueraria montana*, *Astragalus Radix*, *Radix Paeoniae Albab*, *Scrophularia striata*, *Angelica*, *Spatholobus suberectus*, *Atractylodes lancea*, *Paeoniae Radix Rubra*, *Ligusticum Chuanxiong*, *Safflower*, *Prunus davidiana*, *Earthworms*, *Sinapis alba L.*	Mecobalamin Prostaglandin E	2 weeks	⑧
Ji (2010) (107)	China	9.45 ± 2.73/8.70 ± 2.95 (years)	3.77 ± 1.16/3.44 ± 1.29 (years)	60.78 ± 4.26/62.24 ± 4.13	80 (40/40)	Acupuncture Acupoints: SP10, ST36, SP6, LI11, LI4, LR3, RN12, SP9, ST40, SP8.	Mecobalamin	4 weeks	⑧
Zhang (2010) (108)	China	NR	1.0-5.0/0.5-5.0 (years)	36.0-68.0/40.0-66.0	64 (32/32)	Acupuncture Acupoints: BL18, BL20, BL23, BL58, ST36, SP6, SP3, CV6, CV4, ST40, GB34.	Inositol	10 weeks	⑧
Ma (2010) (109)	China	6.0 ± 1.2 (years)	3.4 ± 1.3 (years)	45.4 ± 7.6	68 (34/34)	Acupuncture Acupoints: SP10, ST36, SP6, LI11, LI4, BL17, PC6, RN6, GB39.	Mecobalamin	6 weeks	⑧
Chen (2009) (110)	China	12.06 ± 7.69 (years)	2.23 ± 1.52 (years)	68.55 ± 8.91	71 (38/33)	Acupuncture Acupoints: ST36, SP6, BL17, BL23, PC6, GB34, SJ5, SP9.	Mecobalamin	8 weeks	④⑥⑦
Zhao (2007) (111)	China	9.91 ± 5.28/9.87 ± 4.95	2.71 ± 2.58/2.61 ± 2.22	62.30 ± 7.33/62.17 ± 7.93	60 (30/30)	Acupuncture Acupoints: ST36, SP6, LI11, BL23, BL20, BL18, GB34, KI3.	Mecobalamin	8 weeks	⑧
He (2005) (112)	China	NR	1.0-96.0/0.5-72 (months)	55.3 ± 2.6/53.9 ± 1.9	78 (42/36)	Electroacupuncture Acupoints: ST36, LI11, LI4, GB34, SJ5, LI15, ST41, ST44.	Mecobalamin	4 weeks	④⑦⑧
Xue (2004) (113)	China	7.5 ± 1.0 (years)	3.9 ± 0.8 (years)	63.03 ± 9.84	68 (34/34)	Electroacupuncture Acupoints: SP10, ST36, SP6, LI11, LI4, PC6, GB34, ST40, RN12.	Mecobalamin	8 weeks	⑥⑦⑧
Jin (2003) (114)	China	5.0-20.0/6.0-22.0 (years)	1.0-8.0/2.0-7.0 (years)	49.0-68.0/47.0-69.0	202 (103/99)	Chinese Patent Medicine Tangmai Tong pills: *Salvia miltiorrhiza*, Draconis Sanguis, *Arisaema cum bile*, *Astragalus Radix*, *Sinapis alba L.*	Mecobalamin	8 weeks	④⑤⑥⑦
Yang (2002) (115)	China	4.0-21.0/3.0-19.0 (years)	5.0-156.0/1.0-144.0 (months)	NR	72 (36/36)	Electroacupuncture Acupoints: ST36, KI1.	Vitamin B12	2 weeks	⑧
Pan (2002) (116)	China	NR	1.0-36.0 (months)	42.0-78.0	60 (30/30)	TCM Decoction Chinese medicinal formulae: *Pueraria montana*, *Astragalus Radix*, *Achyranthes bidentata*, *Polygonatum*, *Ligusticum Chuanxiong*, *Safflower*, *Earthworms*, *Trichosanthis radix*, *Eupolyphaga steleophaga*, *Arisaema cum bile*, Coptis chinensis, Prepared rhubarb.	Vitamin B1 Vitamin B12	8 weeks	⑧
Li (2000) (117)	China	8.14 ± 6.16	1.62 ± 1.58	63.03 ± 9.84	84 (48/36)	Electroacupuncture Acupoints: SP6, BL23, BL20, RN6, RN4, ST40, GB30, BL58.	Mecobalamin	8 weeks	⑧

N, sample size; T, treatment; C, control; NR, not report; ①FBG; ②2hpg; ③HbA1c; ④Common peroneal nerve-MNCV; ⑤Common peroneal nerve-SNCV; ⑥Median nerve-MNCV; ⑦Median nerve-NSCV; ⑧Total effective rate; ⑨Adverse event rate; ⑩TCSS.

### Risk of bias in studies

**Figure 2:**
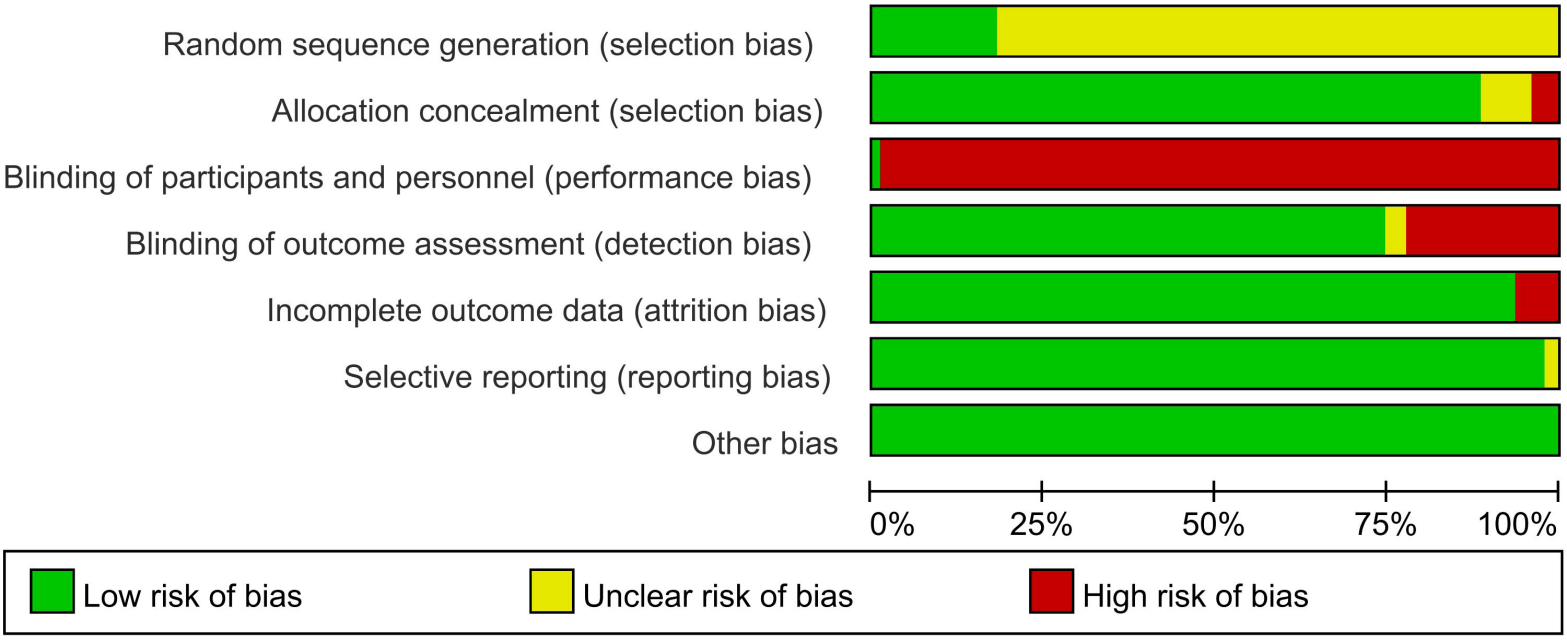
The bias risk of included studies.

Overall, the risk of bias of the RCTs included in this study ranged from low-risk to high. Regarding randomization, about 17.89% (n = 17) of the RCTs were assessed as low risk using a randomized table of numbers. In contrast, the other studies were assessed as an unclear risk for only mentioning randomization without specifying the exact method. 89.47% (n = 85) of the RCTs were assessed as low risk for adequately reporting on the concealment of the allocation scheme; in terms of blinding, 98.95% of the studies were assessed as high risk due to specificity of treatment programs; for blinding of outcome assessment, 74.73% (n = 71) were assessed as low risk; 2.11% (n = 2) selectively reported on the outcome indicators mentioned in the text (but with justification), and were therefore assessed as unclear risk; all RCTs included in the present study were not found to be at risk of cause other biases in risk (**Figure 2**).

### Pairwise meta-analysis

#### Motor conduction velocity of common peroneal nerve

In terms of improving the motor conduction velocity of the common peroneal nerve, TCM Decoction (MD = 3.36, 95%CI 2.97 to 3.75, I^2^ = 79%, *P* < 0.00001), Chinese Patent Medicine (MD = 2.96, 95%CI 1.74 to 4.19, I^2^ = 35%, *P* < 0.00001), acupuncture (MD = 2.47, 95%CI 1.41 to 3.53, I^2^ = 65%, *P* < 0.00001), electroacupuncture (MD = 5.13, 95%CI 3.82 to 6.44, I^2^ = 84%, *P* < 0.00001), TCM Decoction+ Acupuncture (MD = 5.31, 95%CI 4.30 to 6.32, I^2^ = 0%, *P* < 0.00001), TCM Decoction+ Chinese Herbal Footbath (MD = 3.27, 95%CI 2.35 to 4.19, I^2^ = 98%, *P* < 0.00001) all showed an advantage over Western medicines (**Table 2**). The forest plots are recorded in **Supplementary Figure S1**.

**Table 2:** Pairwise meta-analysis results of motor conduction velocity of the common peroneal nerve.

Common peroneal nerve - MNCV					
Comparison	Number of studies	Number of patients	Mean Difference (95%CI)	Heterogeneity test I^2^ (%)	*p* value
TCM Decoction vs. WM	14	1169	3.36 [2.97, 3.75]	79%	*P* < 0.00001
Chinese Patent Medicine vs. WM	3	387	2.96 [1.74, 4.19]	35%	*P* < 0.00001
Acupuncture vs. WM	4	258	2.47 [1.41, 3.53]	65%	*P* < 0.00001
Electroacupuncture vs. WM	2	178	5.13 [3.82, 6.44]	84%	*P* < 0.00001
TCM Decoction+ Acupuncture vs. WM	3	200	5.31 [4.30, 6.32]	0%	*P* < 0.00001
TCM Decoction+ Chinese Herbal Footbath vs. WM	2	269	3.27 [2.35, 4.19]	98%	*P* < 0.00001

#### Sensory conduction velocity of common peroneal nerve

About the sensory conduction velocity of the common peroneal nerve, TCM Decoction (MD = 3.42, 95%CI 3.08 to 3.76, I^2^ = 94%, *P* < 0.00001), Chinese Patent Medicine (MD = 2.60, 95%CI 1.61 to 3.60, I^2^ = 90%, *P* < 0.00001), acupuncture (MD = 3.39, 95%CI 2.24 to 4.54, I^2^ = 0%, *P* < 0.00001), TCM Decoction+ Acupuncture (MD = 6.23, 95%CI 5.00 to 7.46, I^2^ = 90%, *P* < 0.00001), TCM Decoction+ Chinese Herbal Footbath (MD = 10.56, 95%CI 9.61 to 11.51, I^2^ = 99%, *P* < 0.00001) had a positive effect, the detailed results are recorded in **Table 3**, and the forest plots are recorded in **Supplementary Figure S2**.

**Table 3:** Pairwise meta-analysis results of sensory conduction velocity of the common peroneal nerve.

Common peroneal nerve - SNCV					
Comparison	Number of studies	Number of patients	Mean Difference (95%CI)	Heterogeneity test I^2^ (%)	*p* value
TCM Decoction vs. WM	11	847	3.42 [3.08, 3.76]	94%	*P* < 0.00001
Chinese Patent Medicine vs. WM	3	387	2.60 [1.61, 3.60]	90%	*P* < 0.00001
Acupuncture vs. WM	3	190	3.39 [2.24, 4.54]	0%	*P* < 0.00001
TCM Decoction+ Acupuncture	3	200	6.23 [5.00, 7.46]	90%	*P* < 0.00001
TCM Decoction+ Chinese Herbal Footbath vs. WM	2	269	10.56 [9.61, 11.51]	99%	*P* < 0.00001

#### Motor conduction velocity of median nerve

In the realm of enhancing motor conduction velocity of median nerve, the efficacy of TCM Decoction (MD = 3.91, 95%CI 3.51 to 4.30, I^2^ = 95%, *P* < 0.00001), Chinese Patent Medicine (MD = 3.52, 95%CI 2.50 to 4.54, I^2^ = 97%, *P* < 0.00001), electroacupuncture (MD = 7.00, 95%CI 5.18 to 8.83, I^2^ = 95%, *P* < 0.00001), and TCM Decoction+ Acupuncture (MD = 6.42, 95%CI 5.34 to 7.50, I^2^ = 0%, *P* < 0.00001) has demonstrated superiority when contrasted with conventional Western medicine. Conversely, the therapeutic outcomes of acupuncture alone (MD = 1.54, 95%CI 0.44 to 2.65, I^2^ = 95%, *P* < 0.00001) seem to align closely in efficacy with those of Western medicine. Results are illustrated in **Table 4**, with the forest plots in **Supplementary Figure S3**.

**Table 4:** Results of pairwise meta-analysis of motor conduction velocity of the median nerve.

Median nerve - MNCV					
Comparison	Number of studies	Number of patients	Mean Difference (95%CI)	Heterogeneity test I^2^ (%)	*p* value
TCM Decoction vs. WM	11	953	2.38 [2.11, 2.64]	95%	*P* < 0.00001
Chinese Patent Medicine vs. WM	4	519	3.52 [2.50, 4.54]	97%	*P* < 0.00001
Acupuncture vs. WM	3	198	1.54 [0.44, 2.65]	0%	*P* = 0.006
Electroacupuncture vs. WM	2	168	7.00 [5.18, 8.83]	95%	*P* < 0.00001
TCM Decoction+ Acupuncture vs. WM	3	200	6.42 [5.34, 7.50]	0%	*P* < 0.00001

#### Sensory conduction velocity of median nerve

In terms of increasing sensory conduction velocity of the median nerve, TCM Decoction (MD = 2.66, 95%CI 2.31 to 3.01, I^2^ = 92%, *P* < 0.00001), Chinese Patent Medicine (MD = 4.40, 95%CI 2.29 to 5.51, I^2^ = 81%, *P* < 0.00001), electroacupuncture (MD = 3.65, 95%CI 2.11 to 2.64, I^2^ = 97%, *P* < 0.00001), TCM Decoction+ Acupuncture (MD = 9.07, 95%CI 7.93 to 10.22, I^2^ = 57%, *P* < 0.00001) showed remarkable efficacy compared to Western medicine, however, acupuncture (MD = 1.49, 95%CI 0.21 to 2.77, I^2^ = 61%, *P* = 0.02) did not show an advantage over Western medicine (**Table 5**). The forest plots are shown in **Supplementary Figure S4**.

**Table 5:** Results of pairwise meta-analysis of sensory conduction velocity of the median nerve.

Median nerve-SNCV					
Comparison	Number of studies	Number of patients	Mean Difference (95%CI)	Heterogeneity test I^2^ (%)	*p* value
TCM Decoction vs. WM	11	953	2.66 [2.31, 3.01]	92%	*P* < 0.00001
Chinese Patent Medicine vs. WM	4	519	4.40 [3.29, 5.51]	81%	*P <*0.00001
Acupuncture vs. WM	3	198	1.49 [0.21, 2.77]	61%	*P* = 0.02
Electroacupuncture vs. WM	3	246	3.65 [2.29, 5.01]	94%	*P* < 0.00001
TCM Decoction+ Acupuncture vs. WM	3	200	9.07 [7.93, 10.22]	57%	*P* < 0.00001

### FBG

Regarding fasting glucose, TCM Decoction (MD = -0.46, 95%CI -0.59 to -0.33, I^2^ = 97%, *P* < 0.00001), Chinese Patent Medicine (MD = -1.50, 95%CI -1.90 to -1.09, I^2^ = 0%, *P* < 0.00001), TCM Decoction+ Acupuncture (MD = -0.77, 95%CI -0.17 to -0.47, I^2^ = 95%, *P* < 0.00001) had better efficacy than Western medicine (**Table 6**). Forest plots are recorded in **Supplementary Figure S5**.

**Table 6:** Results of pairwise meta-analysis of FBG.

FBG					
Comparison	Number of studies	Number of patients	Mean Difference (95%CI)	Heterogeneity test I^2^ (%)	*p* value
TCM Decoction vs. WM	6	533	-0.46 [-0.59, -0.33]	97%	*P* < 0.00001
Chinese Patent Medicine vs. WM	3	263	-1.50 [-1.90, -1.09]	0%	*P* < 0.00001
TCM Decoction+ Acupuncture vs. WM	3	260	-0.77 [-1.07, -0.47]	95%	*P* < 0.00001

### 2hPG

The results of pairwise meta-analysis for 2hFG indicated that compared with Western medicine, TCM Decoction (MD = -0.47, 95%CI -0.71 to -0.24, I^2^ = 76%, *P* < 0.00001), Chinese Patent Medicine (MD = -1.76, 95%CI -2.31 to -1.20, I^2^ = 84%, *P* < 0.00001), and TCM Decoction+ Acupuncture (MD = -0.53, 95%CI -0.96 to -0.10, I^2^ = 0%, *P* = 0.02) had better clinical efficacy (**Table 7**). Forest plots are summarized in **Supplementary Figure S6**.

**Table 7:** Results of pairwise meta-analysis of 2hPG.

2hPG					
Comparison	Number of studies	Number of patients	Mean Difference (95%CI)	Heterogeneity test I^2^ (%)	*p* value
TCM Decoction vs. WM	6	533	-0.47 [-0.71, -0.24]	76%	*P* < 0.00001
Chinese Patent Medicine vs. WM	2	199	-1.76 [-2.31, -1.20]	84%	*P* < 0.00001
TCM Decoction+ Acupuncture vs. WM	3	260	-0.53 [-0.96, -0.10]	0%	*P* = 0.02

### TCSS

The results of pairwise meta-analysis of TCSS showed that the efficacy of TCM Decoction (MD = -2.18, 95%CI -2.57 to -1.78, I^2^ = 98%, *P* < 0.0001), TCM Decoction+ Acupuncture (MD = -1.49, 95%CI - -2.23 to -0.75, I^2^ = 0%, *P* < 0.0001), showed clinical superiority over that of Western medicine. In contrast, Chinese Patent Medicine (MD = -0.29, 95%CI -1.16 to 0.58, I^2^ = 61%, *P* = 0.51) did not have this advantage. Detailed information is recorded in **Table 8**, and the forest plots are shown in **Supplementary Figure S7**.

**Table 8:** Results of pairwise meta-analysis of TCSS. .

TCSS					
Comparison	Number of studies	Number of patients	Mean Difference (95%CI)	Heterogeneity test I^2^ (%)	*p* value
TCM Decoction vs. WM	5	440	-2.18 [-2.57, -1.78]	98%	*P* < 0.0001
Chinese Patent Medicine vs. WM	2	187	-0.29 [-1.16, 0.58]	0%	*P* = 0.51
TCM Decoction+ Acupuncture vs. WM	2	190	-1.49 [-2.23, -0.75]	0%	*P* < 0.0001

### Total effective rate

The meta-analysis of total clinical effectiveness showed that TCM Decoction (MD = 4.66, 95%CI 3.88 to 5.59, I^2^ = 0%, *P* < 0.00001), Chinese Patent Medicine (MD = 2.37, 95%CI 1.47 to 3.83, I^2^ = 51%, *P* = 0.0004), acupuncture (MD = 3.31, 95%CI 2.09 to 5.23, I^2^ = 0%, *P* < 0.00001), electroacupuncture (MD = 3.50, 95%CI 2.02 to 6.08, I^2^ = 24%, *P* < 0.00001), TCM Decoction+ Acupuncture (MD = 4.68, 95%CI 2.88 to 7.62, I^2^ = 0%, *P* < 0.00001), TCM Decoction+ Chinese Herbal Footbath (MD = 6.18, 95%CI 4.23 to 9.04, I^2^ = 0%), TCM Decoction+ Chinese Medicine Fumigation (MD = 5.67, 95%CI 2.23 to 14.42, I^2^ = 0%, *P* = 0.0003), Acupoint Injection+ Chinese Medicine Fumigation (MD = 4.11, 95%CI 2.36 to 7.16, I^2^ = 0%, *P* < 0.00001) were superior to Western medicine (**Table 9**). Forest plots are given in **Supplementary Figure S8**.

**Table 9:** Results of pairwise meta-analysis of total effective rate.

Total effective rate					
Comparison	Number of studies	Number of patients	Odds Ratio(95%CI)	Heterogeneity test I^2^ (%)	*p* value
TCM Decoction vs. WM	50	4044	4.66 [3.88, 5.59]	0%	*P* < 0.00001
Chinese Patent Medicine vs. WM	5	460	2.37 [1.47, 3.83]	51%	*P* = 0.0004
Acupuncture vs. WM	7	462	3.31 [2.09, 5.23]	0%	*P* < 0.00001
Electroacupuncture vs. WM	5	382	3.50 [2.02, 6.08]	24%	*P* < 0.00001
TCM Decoction+ Acupuncture vs. WM	8	620	4.68 [2.88, 7.62]	0%	*P* < 0.00001
TCM Decoction+ Chinese Herbal Footbath vs. WM	6	1327	6.18 [4.23, 9.04]	0%	*P* < 0.00001
TCM Decoction+ Chinese Medicine Fumigation vs. WM	3	222	5.67 [2.23, 14.42]	0%	*P* = 0.0003
Acupoint Injection+ Chinese Medicine Fumigation vs. WM	3	316	4.11 [2.36, 7.16]	0%	*P* < 0.00001

### Network meta-analysis

#### Network evidence plot

**Figure 3:**
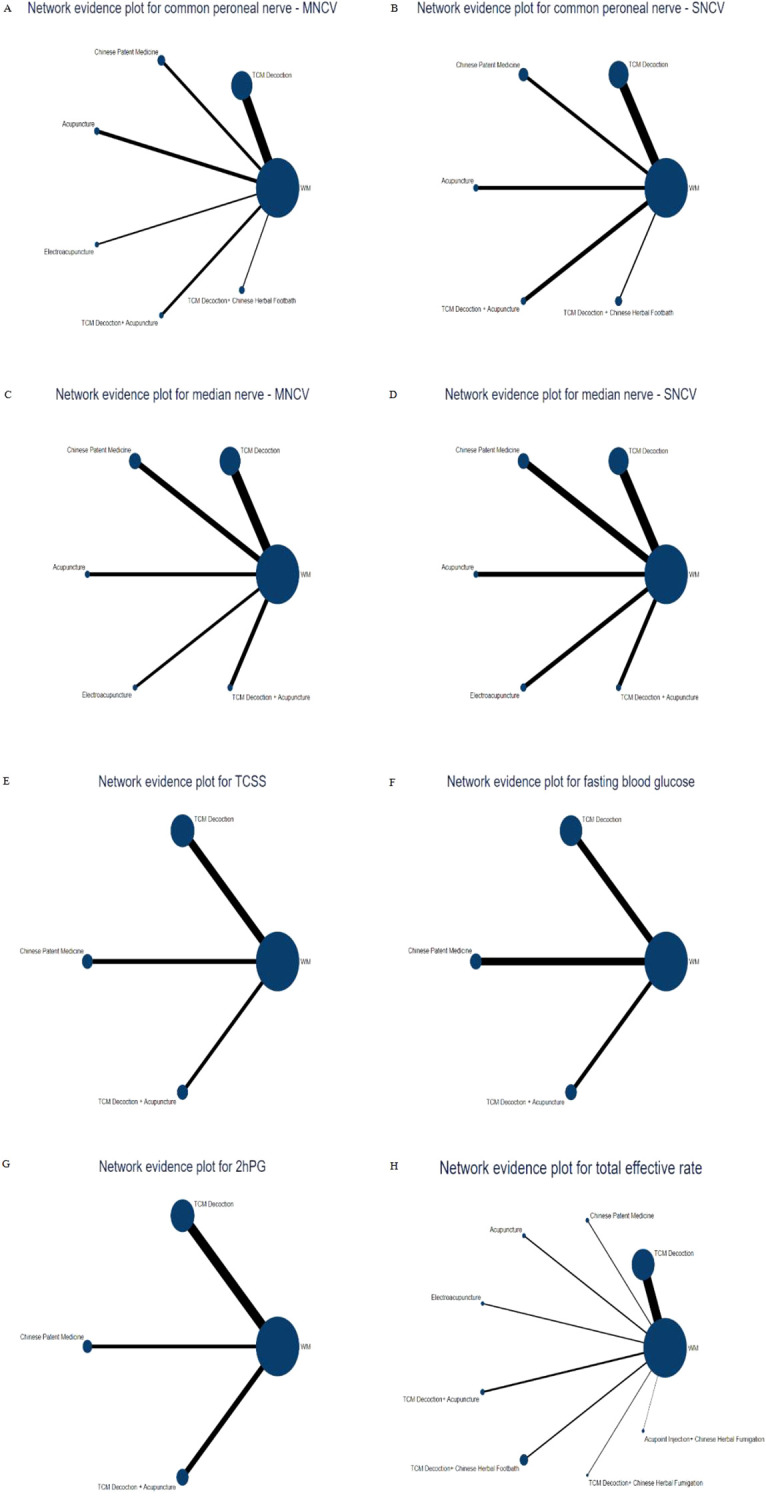
Network evidence plot. **(A)** Network evidence plot for motor conduction velocity of common peroneal nerve; **(B)** Network evidence plot for sensory conduction velocity of common peroneal nerve; **(C)** Network evidence plot for motor conduction velocity of median nerve; **(D)** Network evidence plot for sensory conduction velocity of median nerve; **(E)** Network evidence plot for TCSS; **(F)** Network evidence plot for FBG; **(G)** Network evidence plot for 2hPG; **(H)** Network evidence plot for total effective rate.

28 RCTs reported motor conduction velocity of common peroneal nerve and included a total of 7 therapies, and according to the network evidence plot (**Figure 3A**), it can be seen that TCM Decoction, Chinese Patent Medicine, acupuncture, electroacupuncture, TCM Decoction+ Acupuncture, TCM Decoction+ Chinese Herbal Footbath and Western medicine, with the highest frequency of comparisons between TCM Decoction and Western medicine; 22 RCTs reported sensory conduction velocity of common peroneal nerve (**Figure 3B**), with a total of 6 therapies, including TCM Decoction, Chinese Patent Medicine, acupuncture, TCM Decoction+ Acupuncture, TCM Decoction+ Chinese Herbal Footbath, and Western medicine; regarding the median nerve, the therapies involved included TCM Decoction, Chinese Patent Medicine, acupuncture, electroacupuncture, TCM Decoction+ Acupuncture, and Western medicine, of which 23 RCTs reported motor conduction velocity of median nerve and 24 RCTs reported sensory conduction velocity of median nerve, and the network evidence plots are shown in **Figures 3C, D**. 9 RCTs reported TCSS, involving 4 therapies, TCM Decoction, Chinese Patent Medicine, TCM Decoction+ Acupuncture, and Western medicine (**Figure 3E**); 12 RCTs reported FBG, 11 RCTs reported 2hPG, involving a total of 4 therapies, TCM Decoction, Chinese Patent Medicine, TCM Decoction+ Acupuncture, and Western medicine (**Figures 3F, G**); 87 RCTs reported total effective rate, involving 9 therapies, including TCM Decoction, Chinese Patent Medicine, acupuncture, electroacupuncture, TCM Decoction + Acupuncture, TCM Decoction + Chinese Herbal Footbath, TCM Decoction + Chinese Medicine Fumigation, Acupoint Injection + Chinese Medicine Fumigation, and Western Medicine (**Figure 3H**).

#### Motor conduction velocity of common peroneal nerve

NMA results for motor conduction velocity of the common peroneal nerve showed that TCM Decoction (MD = -3.19, 95%CI -4.36 to -2.02), Chinese Patent Medicine (MD = -2.41, 95%CI -5.16 to 0.45), Acupuncture (MD = -2.729, 95%CI -5.07 to -0.42), Electroacupuncture (MD = -5.00, 95%CI -8.23 to -1.79), TCM Decoction+ Acupuncture (MD = -5.33, 95%CI -8.05 to -2.60), TCM Decoction+ Chinese Herbal Footbath (MD = -5.28, 95%CI -8.44 to -2.21) were all superior to Western medicine. TCM decoction combined + Acupuncture outperformed TCM decoction alone (MD = -2.13, 95% CI -5.10 to 0.88) and Chinese Patent Medicine (MD = -2.94, 95% CI -6.85 to 0.98). Comparisons between other therapies showed no statistically significant differences (**Table 10**).

**Table 10:** NMA results of motor conduction velocity of the common peroneal nerve.

WM						
**-3.19 (-4.36, -2.02)**	TCM Decoction					
**-2.41 (-5.16, 0.45)**	0.79 (-2.19, 3.87)	CPM				
**-2.73 (-5.07, -0.42)**	0.47 (-2.15, 3.03)	-0.30 (-4.03, 3.28)	Acu			
**-5.00 (-8.23, -1.79)**	-1.81 (-5.22, 1.61)	-2.572 (-6.88, 1.62)	-2.26 (-6.25, 1.64)	EA		
**-5.33 (-8.04, -2.60)**	**-2.13 (-5.10, 0.88)**	**-2.94 (-6.85, 0.98)**	-2.60 (-6.22, 1.04)	-0.34 (-4.55, 3.86)	TCM Decoction+ Acu	
**-5.28 (-8.44, -2.21)**	-2.08 (-5.44, 1.14)	-2.86 (-7.09, 1.22)	-2.55 (-6.42, 1.25)	-0.29 (-4.84, 4.12)	0.01 (-4.13, 4.12)	TCM Decoction+ CHF

WM, Western medicine; CPM, Chinese Patent Medicine; Acu, acupuncture; EA, electroacupuncture; CHF, Chinese Herbal Footbath.The bold font indicates a statistical diﬀerence.

The SUCRA values and probability rank results of relevant TCM therapies to improve the motor conduction velocity of the common peroneal nerve in DPN patients were TCM Decoction + Acupuncture (SUCRA = 0.81), TCM Decoction + Chinese Herbal Footbath (SUCRA = 0.80), electroacupuncture (SUCRA = 0.75), TCM Decoction (SUCRA = 0.45), acupuncture (SUCRA = 0.36), Chinese Patent Medicine (SUCRA = 0.33), WM (SUCRA = 0.01) (**Table 11**).

**Table 11:** Rank of the interventions for motor conduction velocity of common peroneal nerve.

Rank	Treatment	SUCRA
1	TCM Decoction+ Acu	0.81
2	TCM Decoction+ CHF	0.80
3	EA	0.75
4	TCM Decoction	0.45
5	Acu	0.36
6	CPM	0.33
7	WM	0.01

WM, Western medicine; CPM, Chinese Patent Medicine; Acu, acupuncture; EA, electroacupuncture; CHF, Chinese Herbal Footbath.

#### Sensory conduction velocity of common peroneal nerve

The results of NMA for sensory conduction velocity of common peroneal nerve showed that compared with Western medicine, TCM Decoction (MD = -4.06, 95%CI -6.47 to -1.76), TCM Decoction + Acupuncture (MD = -7.50, 95%CI -12.29 to -2.86, TCM Decoction + Chinese Herbal Footbath (MD = -8.30, 95%CI -13.75 to -2.73) were found to be superior, while the remaining therapies were not statistically significant when compared to each other (**Table 12**).

**Table 12:** NMA results of sensory conduction velocity of the common peroneal nerve.

WM					
**-4.06 (-6.47, -1.76)**	TCM Decoction				
-2.20 (-6.80, 2.37)	1.859 (-3.27, 6.99)	CPM			
-3.26 (-7.89, 1.37)	0.79 (-4.38, 6.01)	-1.04 (-7.58, 5.46)	Acu		
**-7.50 (-12.29, -2.86)**	-3.45 (-8.72, 1.76)	-5.30 (-11.85, 1.24)	-4.22 (-10.79, 2.24)	TCM Decoction + Acu	
**-8.30 (-13.75, -2.73)**	-4.26 (-10.26, 1.79)	-6.12 (-13.33, 1.01)	-5.07 (-12.29, 2.16)	-0.83 (-8.08, 6.42)	TCM Decoction + CHF

WM, Western medicine; CPM, Chinese Patent Medicine; Acu, acupuncture; EA, electroacupuncture; CHF, Chinese Herbal Footbath.The bold font indicates a statistical diﬀerence.

The SUCRA values and rank of TCM therapies to improve the sensory conduction velocity of the common peroneal nerve in patients with DPN were as follows: TCM Decoction + Chinese Herbal Footbath (SUCRA = 0.87), TCM Decoction + Acupuncture (SUCRA = 0.83), TCM Decoction (SUCRA = 0.51), acupuncture (SUCRA = 0.41), Chinese Patent Medicine (SUCRA = 0.30), and WM ((SUCRA = 0.04) (**Table 13**).

**Table 13:** Rank of the interventions for sensory conduction velocity of common peroneal nerve.

Rank	Treatment	SUCRA
1	TCM Decoction + CHF	0.87
2	TCM Decoction + Acu	0.83
3	TCM Decoction	0.51
4	Acu	0.41
5	CPM	0.30
6	WM	0.04

WM, Western medicine; CPM, Chinese Patent Medicine; Acu, acupuncture; EA, electroacupuncture; CHF, Chinese Herbal Footbath.

#### Motor conduction velocity of median nerve

The NMA results for motor conduction velocity of median nerve demonstrated that TCM Decoction (MD = -3.70, 95%CI -6.12 to -1.27), Electroacupuncture (MD = -6.90, 95%CI -12.70 to -1.08), TCM Decoction+ Acupuncture (MD = -6.57, 95%CI -11.29 to -1.87) were more effective than Western medicine. The comparison between the remaining therapies was not statistically significant (**Table 14**).

**Table 14:** NMA results of motor conduction velocity of the median nerve.

WM					
**-3.70 (-6.12, -1.27)**	TCM Decoction				
-2.70 (-6.79, 1.49)	1.01 (-3.79, 5.91)	CPM			
-1.33 (-6.08, 3.36)	2.36 (-2.93, 7.68)	1.35 (-4.95, 7.64)	Acu		
**-6.90 (-12.70, -0.96)**	-3.16 (-9.46, 3.21)	-4.18 (-11.41, 2.96)	-5.55 (-13.08, 2.07)	EA	
**-6.57 (-11.43, -1.87)**	-2.86 (-8.29, 2.52)	-3.87 (-10.36, 2.40)	-5.24 (-12.04, 1.51)	0.25 (-7.30, 7.85)	TCM Decoction + Acu

WM, Western medicine; CPM, Chinese Patent Medicine; Acu, acupuncture; EA, electroacupuncture.The bold font indicates a statistical diﬀerence.

The SUCRA values and rank of TCM therapies to improve the motor conduction velocity of the median nerve were as follows: electroacupuncture (SUCRA = 0.83), TCM Decoction + Acupuncture (SUCRA = 0.83), TCM Decoction (SUCRA = 0.55), Chinese Patent Medicine (SUCRA = 0.42), acupuncture (SUCRA = 0.27), and Western medicine (SUCRA = 0.07) (**Table 15**).

**Table 15:** Rank of the interventions for motor conduction velocity of the median nerve.

Rank	Treatment	SUCRA
1	EA	0.83
2	TCM Decoction + Acu	0.82
3	TCM Decoction	0.55
4	CPM	0.42
5	Acu	0.27
6	WM	0.07

WM, Western medicine; CPM, Chinese Patent Medicine; Acu, acupuncture; EA, electroacupuncture.

#### Sensory conduction velocity of median nerve

For sensory conduction velocity of median nerve, TCM Decoction (MD = -2.62, 95%CI -4.28 to -0.93), Chinese Patent Medicine (MD = -3.00, 95%CI -5.91 to -0.66), electroacupuncture (MD = - 4.67, 95%CI -8.07 to -1.31), and TCM Decoction + Acupuncture (MD = -8.26, 95%CI -11.59 to -0. 4.8) were more effective than Western medicine. In addition, TCM Decoction + Acupuncture was more effective than TCM Decoction (MD = -5.64, 95%CI -9.43 to -1.86), Chinese Patent Medicine (MD = -5.28, 95%CI -9.87 to -0.75), and acupuncture (MD = -7.14, 95%CI -11.87 to -2.3) (**Table 16**).

**Table 16:** NMA results of sensory conduction velocity of the median nerve.

WM					
**-2.62 (-4.28, -0.93)**	TCM Decoction				
**-3.00 (-5.91, 0.06)**	-0.38 (-3.74, 3.11)	CPM			
-1.12 (-4.49, 2.19)	1.50 (-2.23, 5.22)	1.85 (-2.65, 6.33)	Acu		
**-4.67 (-8.07, -1.31)**	-2.03 (-5.83, 1.67)	1.66 (-6.31, 2.79)	-3.55 (-8.39, 1.32)	EA	
**-8.26 (-11.59, -4.85)**	**-5.64 (-9.43, -1.86)**	**-5.28 (-9.87, -0.75)**	**-7.14 (-11.87, -2.36)**	-3.59 (-8.36, 1.19)	TCM Decoction + Acu

WM, Western medicine; CPM, Chinese Patent Medicine; Acu, acupuncture; EA, electroacupuncture.The bold font indicates a statistical diﬀerence.

In terms of improving the median nerve’s sensory conduction velocity, TCM Decoction + Acupuncture (SUCRA = 0.98) became the best therapy, followed by electroacupuncture (SUCRA = 0.72), Chinese Patent Medicine (SUCRA = 0.51), TCM Decoction (SUCRA = 0.46), acupuncture (SUCRA = 0.24), and Western medicine (**Table 17**).

**Table 17:** Rank of the interventions for sensory conduction velocity of the median nerve.

Rank	Treatment	SUCRA
1	TCM Decoction + Acu	0.98
2	EA	0.72
3	CPM	0.51
4	TCM Decoction	0.46
5	Acu	0.24
6	WM	0.05

WM, Western medicine; CPM, Chinese Patent Medicine; Acu, acupuncture; EA, electroacupuncture.

### TCSS

The NMA results from the TCSS showed no statistically significant differences in the comparisons between the 5 therapies (**Table 18**). Possible rank results are shown in **Table 19**.

**Table 18:** NMA results of TCSS. WM: Western medicine.

WM			
1.86 (0.28, 3.41)	TCM Decoction		
0.31 (-2.19, 2.90)	-1.55 (-4.47, 1.53)	CPM	
1.44 (-1.05, 3.96)	-0.42 (-3.43, 2.58)	1.11 (-2.50, 4.65)	TCM Decoction + Acu

WM, Western medicine; CPM, Chinese Patent Medicine; Acu, acupuncture.

**Table 19:** Rank of the interventions for TCSS.

Rank	Treatment	SUCRA
1	TCM Decoction	0.82
2	TCM Decoction + Acu	0.67
3	CPM	0.33
4	WM	0.17

WM, Western medicine; CPM, Chinese Patent Medicine; Acu, acupuncture.

### FBG

The NMA results for fasting glucose showed that comparisons between treatments were not statistically significant (**Table 20**). The rank results are shown in **Table 21**.

**Table 20:** NMA results of FBG.

WM			
1.26 (-0.10, 2.61)	TCM Decoction		
1.44 (-0.58, 3.48)	0.23 (-2.23, 2.67)	CPM	
1.68 (-0.24, 3.63)	0.44 (-1.91, 2.83)	0.20 (-2.54, 3.01)	TCM Decoction + Acu

WM, Western medicine; CPM, Chinese Patent Medicine; Acu, acupuncture.

**Table 21:** Rank of the interventions for FBG.

Rank	Treatment	SUCRA
1	TCM Decoction + Acu	0.73
2	CPM	0.64
3	TCM Decoction	0.57
4	WM	0.04

WM, Western medicine; CPM, Chinese Patent Medicine; Acu, acupuncture.

### 2hPG

Regarding 2hPG, there are no statistically significant differences in the comparisons between the various TCM therapies (**Table 22**), and the possible rank results are shown in the **Table 23**.

**Table 22:** NMA results of 2hPG.

WM			
0.70 (-0.02, 1.44)	TCM Decoction		
1.45 (0.07, 2.68)	0.74 (-0.82, 2.16)	CPM	
0.64 (-0.39, 1.76)	-0.05 (-1.32, 1.30)	-0.80 (-2.39, 1.01)	TCM Decoction + Acu

WM, Western medicine; CPM, Chinese Patent Medicine; Acu, acupuncture.

**Table 23:** Rank of the interventions for 2hPG.

Rank	Treatment	SUCRA
1	CPM	0.89
2	TCM Decoction	0.55
3	TCM Decoction + Acu	0.50
4	WM	0.04

WM, Western medicine; CPM, Chinese Patent Medicine; Acu, acupuncture.

### Total effective rate

The NMA results of the total effective rate of TCM therapies to improve DPN showed that compared to Western medicine, TCM Decoction (OR = 0.21, 95%CI 0.17 to 0.25), Chinese Patent Medicine (OR = 0.41, 95%CI 0.24 to 0.69), acupuncture (OR = 0.29, 95%CI 0.17 to 0.47), electroacupuncture (OR = 0.28, 95%CI 0.16 to 0.49), TCM Decoction + Acupuncture (OR = 0.21, 95%CI 0.13 to 0.33), TCM Decoction + Chinese Herbal Footbath (OR = 0.16, 95%CI 0.10 to 0.23), TCM Decoction + Chinese Medicine Fumigation (OR = 0.16, 95%CI 0.06 to 0.38), Acupoint Injection + Chinese Medicine Fumigation (OR = 0.24, 95%CI 0.13 to 0.42) all achieved better clinical efficacy, in addition, TCM Decoction + Chinese Herbal Footbath was more effective than Chinese Patent Medicine (OR = 0.38, 95%CI 0.20 to 0.75) for overall symptom improvement in DPN patients. Other therapies were not statistically significant in a two-by-two comparison (**Table 24**). TCM Decoction + Chinese Herbal Football (SUCRA = 0.85) became the best therapy among TCM therapies; the rank of the TCM therapies is shown in **Table 25**.

**Table 24:** NMA results of total effective rate.

WM								
**0.21 (0.17, 0.25)**	TCM Decoction							
**0.41 (0.24, 0.69)**	2.00 (1.13, 3.48)	CPM						
**0.29 (0.17, 0.47)**	1.40 (0.8, 2.35)	0.71 (0.34, 1.34)	Acu					
**0.28 (0.16, 0.49)**	1.33 (0.75, 2.4)	0.67 (0.33, 1.39)	0.96 (0.49, 1.94)	EA				
**0.21 (0.13, 0.33)**	1.00 (0.60, 1.64)	0.51 (0.24, 1.04)	0.73 (0.38, 1.35)	0.75 (0.34, 1.6)	TCM Decoction + Acu			
**0.16 (0.1, 0.23)**	0.77 (0.48, 1.18)	**0.38 (0.20, 0.75)**	0.55 (0.28, 1.06)	0.57 (0.29, 1.11)	0.76 (0.41, 1.40)	TCM Decoction + CHF		
**0.16 (0.06, 0.38)**	0.77 (0.27, 1.86)	0.39 (0.12, 1.03)	0.54 (0.19, 1.53)	0.55 (0.18, 1.61)	0.74 (0.25, 2.26)	0.98 (0.33, 2.71)	TCM Decoction + CMF	
**0.24 (0.13, 0.42)**	1.17 (0.62, 2.07)	0.59 (0.27, 1.25)	0.84 (0.39, 1.86)	0.86 (0.39, 1.9)	1.16 (0.54, 2.38)	1.51 (0.75, 3.04)	1.58 (0.49, 4.91)	Acupoint Injection + CMF

WM, Western medicine; CPM, Chinese Patent Medicine; Acu, acupuncture; EA, electroacupuncture; CHF, Chinese Herbal Footbath; CMF, Chinese Medicine Fumigation.The bold font indicates a statistical diﬀerence.

**Table 25:** Rank of the interventions for total effective rate.

Rank	Treatment	SUCRA
1	TCM Decoction + CHF	0.85
2	TCM Decoction + CMF	0.80
3	TCM Decoction	0.65
4	TCM Decoction + Acu	0.64
5	Acupoint Injection + CMF	0.55
6	EA	0.41
7	Acu	0.37
8	CPM	0.19
9	WM	0.01

WM, Western medicine; CPM, Chinese Patent Medicine; Acu, acupuncture; EA, electroacupuncture; CHF, Chinese Herbal Footbath; CMF, Chinese Medicine Fumigation.

### Adverse events

8 studies reported adverse events, as shown in **Table 26**. Adverse events that occurred in the treatment group included stomach discomfort, such as nausea and vomiting. In addition to the above, two slightly more serious adverse events were reported in the Western medicine group, which were impaired liver and kidney function. In addition, the acupoint injection combined with the Chinese Medicine Fumigation group reported 4 cases of skin erythema and 3 of sensitization. Overall, the number of adverse events in the treatment group was lower than in the control group, and no serious adverse events occurred.

**Table 26:** Adverse events.

Author (year)	Therapy(T/C)	Adverse events (T) (d/N)	Adverse events (C) (d/N)	Details
Ma 2022 (96)	TCM Decoction/WM	1/48	3/48	T: 1 case of diarrhea. C: 1 case of nausea, 1 case of vomiting, and 1 case of abnormalities of liver and kidney function.
Yang 2021 (34)	TCM Decoction/WM	2/25	3/24	T: 1 case of stomach discomfort and 1 case of nausea and vomiting. C: 1 case of dizziness, 1 case of stomach discomfort, and 1 case of nausea and vomiting.
Hu 2021 (35)	TCM Decoction/WM	2/33	5/33	T: 1 case of nausea and 1 case of loss of appetite. C: 1 case of nausea, 2 cases of loss of appetite, 1 case of diarrhea, and 1 case of rash.
Zhang 2019 (52)	TCM Decoction/WM	2/23	10/22	T: 1 case of headache, 1 case of nausea and vomiting. C: 3 cases of headache, 2 cases of nausea and vomiting, 2 cases of chest tightness and palpitations, and 3 cases of instances of acid belching.
Cheng 2017 (78)	TCM Decoction/WM	0/42	5/42	T: none. C: NR.
Guo et al., 2019 (52)	Chinese Patent Medicine/WM	2/49	10/49	T: 1 case of nausea and 1 case of vomiting. C: 3 cases of nausea, 4 cases of vomiting, and 3 cases of stomach discomfort.
Li et al., 2014 (103)	Chinese Patent Medicine/WM	1/23	4/23	T: 1 case of stomach discomfort. C: 1 case of nausea and 3 cases of stomach discomfort.
Chen 2017 (73)	Acupoint Injection+ Chinese Medicine Fumigation/WM	0/33	7/33	T: none. C: 4 cases of skin erythema and 3 cases of sensitization.

### Subgroup analysis

Due to the significant heterogeneity in some results, we conducted subgroup analyses (**Supplementary Figure S9**). The findings from subgroup analyses indicated that various TCM decoction prescriptions did not significantly impact the conduction velocity of the common peroneal nerve and median nerve (*P* > 0.05). Additionally, for the conduction velocity of the common peroneal nerve, varying durations of acupuncture treatment did not reveal any significant differences in effect size (*P* > 0.05).

### Sensitivity analysis

**Figure 4:**
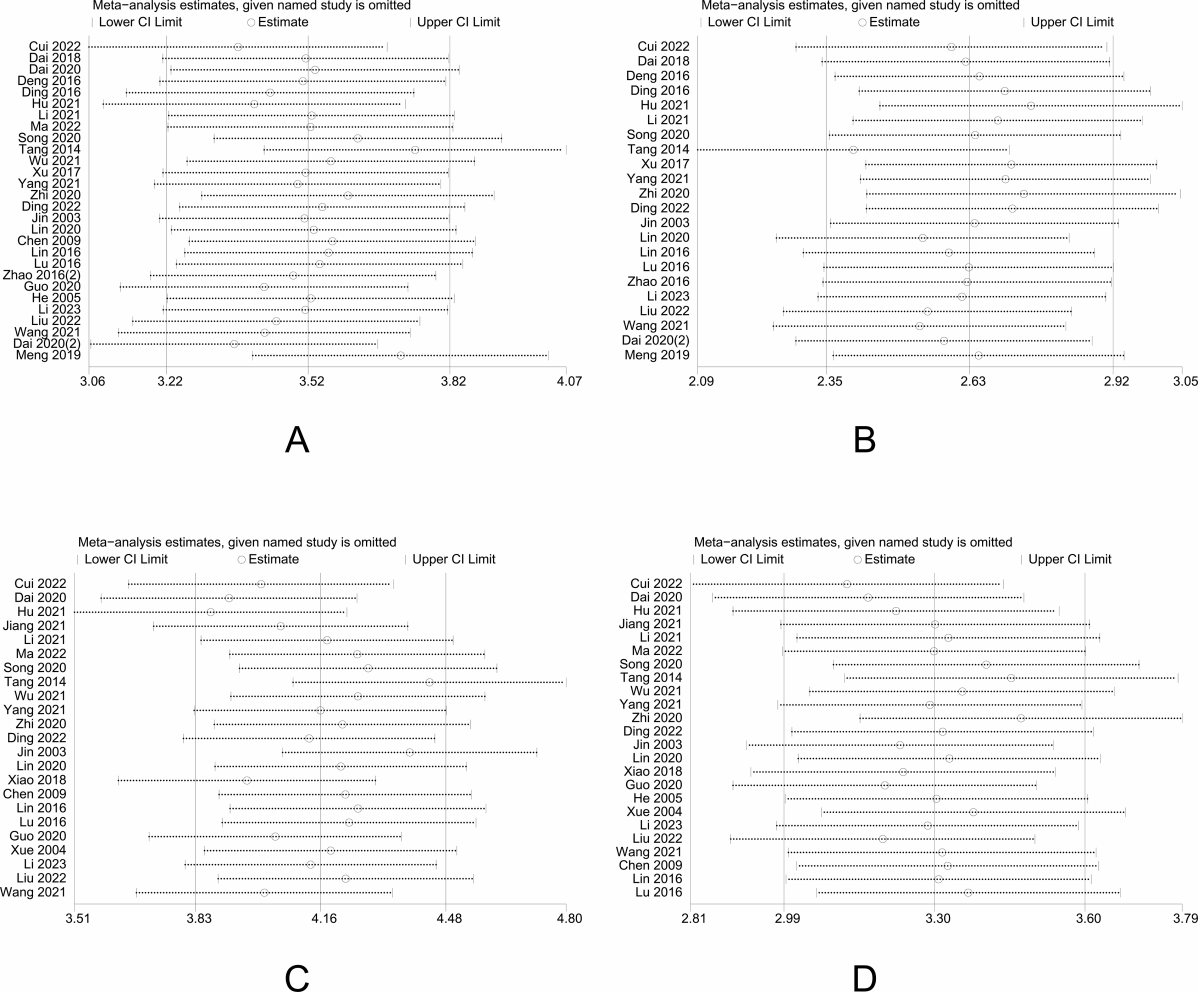
Results of sensitivity analysis. **(A)** sensitivity analysis result for motor conduction velocity of common peroneal nerve; **(B)** sensitivity analysis result for sensory conduction velocity of common peroneal nerve; **(C)** sensitivity analysis result for motor conduction velocity of median nerve; **(D)** sensitivity analysis result for sensory conduction velocity of median nerve.

We performed a sensitivity analysis of the indicators related to electromyography, including the motor conduction velocity of the common peroneal nerve, the sensory conduction velocity of the common peroneal nerve, the motor conduction velocity of the median nerve, and the sensory conduction velocity of the median nerve, and confirmed the stability of the results. The results of the sensitivity analysis are shown in **Figure 4**.

### Publication bias

**Figure 5:**
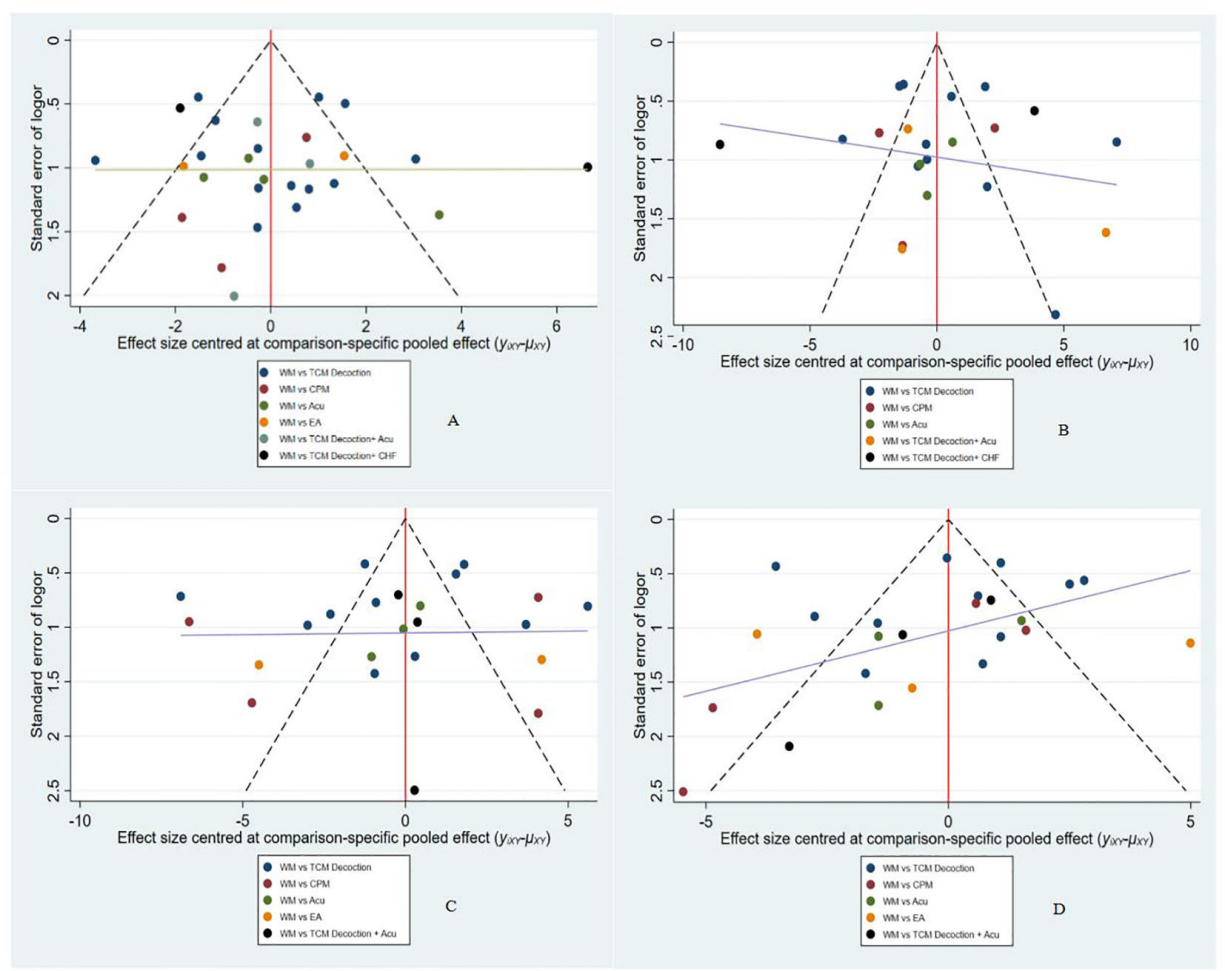
Publication bias. **(A)** funnel plot for motor conduction velocity of common peroneal nerve; **(B)** funnel plot for sensory conduction velocity of common peroneal nerve; **(C)** funnel plot for motor conduction velocity of median nerve; **(D)** funnel plot for sensory conduction velocity of median nerve.

Stata 17.0 generated a comparison-corrected funnel plot (**Figure 5**). The funnel plot indicated the possibility of a small sample effect, so an Egger test was also conducted to further verify the existence of publication bias. The results of the Egger test are shown in the **Supplementary Figure S10**. The Egger test showed that *P* > 0.05, indicating that there was no publication bias.

### Quality of evidence

The quality of the evidence was evaluated using the GRADE profiler, which showed that most of the evidence was downgraded due to the presence of risk of bias and inconsistency, with the quality of the evidence graded from very low to high (**Supplementary Figure S11**).

## Discussion

In addition to blood glucose, many metabolic factors also play an important role in the development of DPN, so the focus of the treatment of DPN needs to be changed from simple glycemic control to multi-targeted therapy (118). According to previous investigations, glycemic control can only intervene in the development of DPN in T1DM but is ineffective in treating DPN caused by T2DM. As the research on TCM in treating DPN becomes more and more extensive, it has been found that TCM has the advantages of being multi-targeted, having good efficacy, and having mild side effects. The treatment of DPN with TCM is not limited to oral medications but also employs several external therapies or a combination of them, which has achieved significant clinical efficacy (21). Previous studies have explored the effectiveness of TCM external therapies on DPN but have not comprehensively investigated TCM therapies (119). Based on the above, we conducted a comprehensive search of RCTs of all therapies related to TCM for the treatment of DPN. We attempted to find the optimal therapeutic regimen of TCM for treating DPN through the NMA approach.

In this study, we retrieved 3279 TCM RCTs for the treatment of DPN and finally included 95. The 95 RCTs involved 9 therapies, including TCM Decoction, Chinese Patent Medicine, acupuncture, electroacupuncture, TCM Decoction+ Acupuncture, TCM Decoction+ Chinese Herbal Footbath, TCM Decoction+ Chinese Medicine Fumigation, Acupoint Injection+ Chinese Medicine Fumigation, and Western medicine. We evaluated electromyography (common peroneal nerve and median nerve), FBG, 2hPG, TCSS, and total effective rate. Furthermore, our findings indicate that therapies related to TCM can enhance patients’ blood glucose profiles by reducing FBG and 2hPG. It is widely recognized that effective management of blood glucose can alleviate clinical symptoms and halt the progression of the disease in patients with DPN. We evaluated neurophysiological indicators, motor and sensory conduction velocities of the common peroneal and median nerves (reflecting the recovery of nerve fiber function); glycemic indicators, including FBG and 2hPG (reflecting the control of blood glucose); TCSS and the total effective rate (evaluating the improvement of clinical symptoms). The pairwise meta-analysis and NMA results consistently showed that the TCM-related therapies demonstrated more significant clinical advantages in improving nerve conduction velocity than Western medicine. TCM-related therapies effectively improve clinical symptoms in DPN patients. Specifically, in pairwise meta-analysis, TCM therapies significantly improved nerve conduction velocity in DPN patients; however, it is worth noting that the current study did not find any advantage of acupuncture over Western medicine in improving the conduction velocity of the median nerve. Furthermore, our findings indicate that therapies related to TCM can enhance patients’ blood glucose profiles by reducing FBG and 2hPG. It is widely recognized that effective management of blood glucose can alleviate clinical symptoms and halt the progression of the disease in patients with DPN.

We conducted an NMA comparing the nine therapies pairwise for further analysis. The SUCRA values establish the final ranking of the interventions. It is crucial to clarify that while SUCRA values indicate the relative rank order among the treatments, they do not directly convey the magnitude of the effect sizes or the clinical significance of the differences observed. Electromyography (EMG) is one of the objective diagnostic tools for DPN and aids in its early diagnosis. The results of this study showed that for improving motor conduction velocity of common peroneal nerve, the top-ranked therapies compared to WM were: TCM Decoction + Acupuncture (SUCRA = 0.81), TCM Decoction + Chinese Herbal Footbath (SUCRA = 0.80), and Electroacupuncture (SUCRA = 0.75); for sensory conduction velocity of common peroneal nerve, the therapies with the highest probability of ranking top were: TCM Decoction + Chinese Herbal Footbath (SUCRA = 0.87), TCM Decoction + Acupuncture (SUCRA = 0.83), and TCM Decoction (SUCRA = 0.51); The NMA results for motor conduction velocity of median nerve indicated that electroacupuncture (SUCRA = 0.83), TCM Decoction + Acupuncture (SUCRA = 0.82), and TCM Decoction (SUCRA = 0.55) were the top three interventions; for improving sensory conduction velocity of median nerve, the top-ranked therapies were: TCM Decoction + Acupuncture (SUCRA = 0.98), electroacupuncture (SUCRA = 0.72), and Chinese Patent Medicine (SUCRA = 0.51); TCM Decoction + Chinese Herbal Footbath (SUCRA = 0.85) ranked first in improving the overall symptoms of DPN. However, it is important to note that the comparisons between these therapies showed no statistically significant differences. These findings indicate that, compared to WM, combination therapies based on TCM Decoction significantly affect the DPN indicators evaluated. Therefore, we conclude that TCM Decoction combination therapies (specifically Decoction + Acupuncture and TCM Decoction + Chinese Herbal Footbath) demonstrate the most pronounced therapeutic efficacy for DPN, outperforming either WM or single-modality TCM therapies (such as TCM Decoction alone or Acupuncture alone). Furthermore, while Chinese Patent Medicine may show some effect compared to WM (**Tables 10**, **16**), its effect sizes were generally smaller than those of the higher-ranked interventions, suggesting its efficacy might be relatively weaker or associated with greater uncertainty. Consequently, in routine clinical practice, we do not recommend Chinese Patent Medicine as a first-line treatment for DPN. It may be considered as an alternative option for specific patient populations, such as those who cannot tolerate conventional medications or are seeking adjunctive therapy, especially when higher-ranked interventions are unavailable. Patients must be fully informed about the limitations of the evidence regarding the relative efficacy of Chinese Patent Medicine. Although the results of this study showed that TCM therapies showed effectiveness in improving TCSS, FBG, and 2hPG in comparison to Western medicine in pairwise meta-analysis, no statistical difference was found in the two-by-two comparison when performing NMA, so this study did not conclude the optimal intervention for TCM therapies to improve TCSS, FBG, and 2hPG. This study summarized the adverse events reported by 8 RCTs (**Table 26**), including dizziness, gastrointestinal discomfort, skin erythema, etc. The adverse events were mild, and the number of events was very low, so they can be considered safe. The present study yields findings worthy of further investigation. While the pairwise meta-analysis indicated that TCM therapies were effective in improving TCSS, FBG, and 2hPG when compared to Western medicines, the two-by-two direct comparisons within the framework of the current NMA did not reveal statistically significant differences between these therapies or when compared to Western medications. This limitation hinders our ability to draw definitive conclusions regarding the most effective interventions for enhancing TCSS, FBG, and 2hPG. Future research may require larger sample sizes or more refined designs for validation. Regarding safety assessment (**Table 26**), adverse events reported in the eight RCTs included in this study primarily involved mild occurrences such as dizziness, gastrointestinal discomfort, and skin erythema. The frequency of these events was low, and all were mild and self-limiting. Considering the available evidence, the various interventions explored, particularly the top-ranked TCM combination treatment, demonstrate a relatively favorable safety profile for patients with DPN.

Regarding the pathogenesis of DPN, a recent study demonstrated an association with metabolic abnormalities, oxidative stress, endoplasmic reticulum stress, mitochondrial dysfunction, microvascular dysfunction, and inflammation (120). Regarding the pathogenesis of DPN, a recent study demonstrated an association with metabolic abnormalities, oxidative stress, endoplasmic reticulum stress, mitochondrial dysfunction, microvascular dysfunction, and inflammation. In recent years, with in-depth research on the mechanisms of DPN in TCM, many innovative therapeutic options have been provided for clinical management and treatment. Oral medications used in TCM to treat DPN include herbal extracts, Chinese herbal compounds, and Chinese Patent Medicine. Included in this study were Chinese medicine compounds (referred to as TCM Decoction in this study) and Chinese Patent Medicine. The efficacy of TCM Decoction and Chinese Patent Medicine in treating DPN has been recognized in many clinical and mechanism studies. Many clinical and mechanism studies have recognized the efficacy of Chinese herbal compounds and Chinese Patent Medicine in treating DPN. A study in 2023 used metabolomics and microbiome to validate the mechanism of Huangqi Guizhi Wuwu Decoction for treating DPN. It was concluded that Huangqi Guizhi Wuwu Decoction could reduce inflammation and oxidative stress by regulating sphingolipid metabolism, biosynthesis of unsaturated fatty acids, arachidonic acid metabolism, and lactobacilli, thus improving the structure of peripheral nerves and enhancing the sciatic nerve conduction velocity in DPN mice (121). Another study verified that Jiaweibugan decoction regulates glutathione, nuclear factor kappa B p 65, and p38 mitogen-activated protein kinase expression through the anti-oxidative stress pathway, thereby repairing damaged peripheral nerves (122). In Chinese Patent Medicine, a study found that Compound Qiying Granules could improve peripheral nerve fiber myelination lesions through endoplasmic reticulum stress and apoptosis (123). As Chinese medicine research continues to deepen, herbal extracts are created. Herbal extracts are extracted, separated, and processed from herbs using modernized techniques. Studies of their pharmacological mechanisms have provided strong evidence for using TCM in treating DPN. Based on their chemical structures, herbal extracts used in treating DPN can be categorized into glycosides, phenols, alkaloids, flavonoids, etc. Specifically, it includes Notoginsenoside R1 extracted from *Panax notoginseng*, Astragaloside IV extracted from *Astragali radix*, Saikosaponin d extracted from *Radix Bupleuri*, and Puerarin extracted from *Radix puerariae*, etc. The above herbal extracts have been verified to improve DPN through anti-inflammatory, antioxidative, and endoplasmic reticulum stress regulation (124–126). Chinese Herbal Footbath is one of the external treatments of TCM, which enables the pharmacological effects of the above-mentioned herbs to be absorbed directly through the skin of the feet, acupoints, reflex zones, etc., and reach the lesions directly. Acupuncture is another standard external treatment method in TCM. In recent years, acupuncture has been widely researched to treat clinical diseases in various disciplines, and the related mechanism research has gradually improved. Studies have shown that acupuncture can treat DPN by regulating oxidative stress, improving inflammation, and regulating nerve growth factors (127–130). The above provides a reliable basis for our findings. It is well known that DPN patients first present with sensory deficits in the lower extremities, followed by sensory deficits in the upper extremities, and with the progression of the disease, some may present with motor deficits. For most DPN patients, sensory impairment is the main symptom of DPN patients, and this may be a long-term process. For sensory deficits in DPN, our results suggest that the combination therapy of TCM Decoction becomes the best treatment option to improve the sensory conduction velocity of the common peroneal and median nerve; in addition, this study also found that the most effective treatment to improve the overall symptoms of patients with DPN is TCM Decoction + Chinese Herbal Footbath. TCM Decoction has the advantage of multi-target regulation, which can not only directly improve the symptoms of DPN through its pharmacological action but also achieve the purpose of blood glucose regulation through compatibility and slow down the development of DPN (131, 132). Therefore, TCM Decoction combined with Chinese Herbal Footbath or acupuncture is a comprehensive treatment program that combines internal and external treatments to enhance the effect of directly reaching the lesion and effectively targeting the sensory disorders of DPN patients. As for motor dysfunction, although they are less common than sensory impairment, they can still be detected in electromyography. Our results showed that TCM Decoction + Acupuncture emerged as the best intervention to improve the motor conduction velocity of the common peroneal nerve (SUCRA = 0.81), and electroacupuncture emerged as the best intervention to improve the motor conduction velocity of the median nerve (SUCRA = 0.83). Numerous studies have found that patients with DPN have extensive cerebral cortex thinning or reduced grey matter volume in the central nervous system (especially the brain, not just the peripheral nervous system) and that extensive structural damage to the brain may be the underlying cause of the motor dysfunction that occur in patients (133–135). As for motor dysfunction, although they are less common than sensory impairment, they can still be detected in electromyography. Our results showed that TCM Decoction + Acupuncture emerged as the best intervention to improve the motor conduction velocity of the common peroneal nerve (SUCRA = 0.81), and electroacupuncture emerged as the best intervention to improve the motor conduction velocity of the median nerve (SUCRA = 0.83). Numerous studies have found that patients with DPN have extensive cerebral cortex thinning or reduced grey matter volume in the central nervous system (especially the brain, not just the peripheral nervous system) and that extensive structural damage to the brain may be the underlying cause of the motor dysfunction that occur in patients. Previous studies have also found that acupuncture (including electroacupuncture) improves motor function by modulating spinal motor neurons, including the cerebellum (possibly by promoting neuronal repair and regeneration) (136, 137). The above evidence explains the findings of EMG-related indicators well.

Our study strictly followed the Cochrane Collaboration and PRISMA flowcharts. An extensive search was conducted for current TCM RCTs for DPN by developing detailed search strategies. Reliable results were reported in performing both pairwise meta-analysis and NMA; most were statistically significant (*P* < 0.05) in meta-analysis. When performing NMA, we evaluated the model by the Brooks-Gelman-Rubin method, where the model fit was determined by the Potential Scale Reduction Factor (PSRF), and the PSRF values in this study were all close to 1, demonstrating that the model convergence was acceptable. In addition, the results were stable when sensitivity analyses of the primary outcome indicators were performed. Since some studies were outside the 95% CI in the funnel plot when assessing publication bias, proving that there appeared to be minor sample effects, in order to assess whether these small sample effects had an impact on the results of the study, an Egger test was also done, the result of Egger test showed that they did not affect the reliability of the results.

Despite some valuable findings, this study has some limitations. First, the risk of bias and the quality of evidence of the included studies impacted the results. Some of the studies were defined as high-risk due to randomization and blinding; therefore, the quality of evidence was downgraded, affecting the credibility of the results. Indeed, implementing blinding in TCM RCTs faces inherent challenges. These difficulties stem from the fundamental conflict between TCM’s holistic nature, individualized diagnosis, treatment approach, and the standardized requirements of RCTs. Particularly for non-pharmacological therapies like acupuncture, double-blinding is unattainable, which inevitably limits the strength of our evidence. When summarizing the characteristics of the included studies (**Table 1**), we observed that some RCTs failed to provide detailed descriptions of the duration and stage of DPN, with some even lacking data on age and disease duration. In studies utilizing TCM decoctions or Chinese patent medicine, the compositions of the herbal formulations were not standardized. Similarly, acupuncture studies lacked consistency in the acupoints selected. These factors contributed to substantial heterogeneity in some outcomes (I² > 50%). Consequently, we performed subgroup analyses. These analyses revealed that neither different TCM herbal formulations nor varying acupuncture durations for nerve conduction velocity showed significant differences in effect sizes. Regrettably, due to limitations in the original studies, we could not conduct subgroup analyses on other potential sources of heterogeneity, such as age, disease duration, and follow-up periods. As evident in **Table 1**, several studies lacked data on age, disease duration, and follow-up periods. The results are regrettable despite our efforts to contact authors for missing data. Therefore, our findings cannot provide reliable references for patients with DPN of different ages or disease durations. Furthermore, the long-term efficacy of TCM-related therapies for DPN remains uncertain due to the scarcity of follow-up data, highlighting an important area for future research. The NMA results indicate that the 95% confidence intervals for some of the two-by-two comparisons of interventions are quite broad, particularly evident when comparing TCM Decoction + Acupuncture with WM. Such wide intervals may be attributed to the limited sample sizes in the original studies or the inherent heterogeneity of the combination therapies, including variations in herbal prescriptions, dosages, needling techniques, and duration. This underscores the necessity for future studies involving larger sample sizes and more rigorously standardized intervention protocols to provide more conclusive evidence. Despite these uncertainties, sensitivity analysis indicated that our results are reliable. However, the limited number of original studies prevented a quantitative analysis of HbA1c. Additionally, the pairwise comparisons of TCSS, FBG, and 2hPG did not reach statistical significance. Consequently, this study cannot conclude whether TCM therapies improve the TCSS or glycemic control in DPN patients. Future well-designed studies are warranted to validate these findings. It is important to note that the safety data for this study were derived from only eight RCTs, all of which reported minor adverse events related to TCM therapies. Although TCM therapies are generally considered safe, this finding presents some limitations that could restrict the applicability and reproducibility of the results across a broader range of populations, including different subgroups and individuals with comorbidities. Therefore, high-quality studies are necessary to assess the safety of TCM therapies for treating DPN further and to provide more reliable clinical guidance for decision-makers in healthcare.

## Conclusions

The comprehensive TCM therapy can be an effective and relatively safe treatment for DPN. The conservative clinical stratification recommendation sequence is as follows:

1. Motor conduction velocity of common peroneal nerve: TCM Decoction + Acupuncture, TCM Decoction + Chinese Herbal Footbath, and electroacupuncture.
2. Sensory conduction velocity of common peroneal nerve: TCM Decoction + Chinese Herbal Footbath, TCM Decoction + Acupuncture, and TCM Decoction.
3. Motor conduction velocity of median nerve: electroacupuncture, TCM Decoction + Acupuncture, TCM Decoction.
4. Sensory conduction velocity of median nerve: TCM Decoction + Acupuncture, electroacupuncture, and Chinese Patent Medicine.

However, due to limitations in the quality of the included studies, larger sample sizes and high-quality research are still needed.

## Data Availability

The original contributions presented in the study are included in the article/**Supplementary Material**. Further inquiries can be directed to the corresponding authors.
