# Supplementary material for: Traditional Chinese medicine for diabetic peripheral neuropathy: a network meta-analysis
Source: Front Endocrinol (Lausanne). 2025 Aug 27;16:1596924. doi: 10.3389/fendo.2025.1596924 (PMC12420273; doi:10.3389/fendo.2025.1596924)
Supplement: Supplementary file 1 [file DataSheet1.pdf]

Supplementary Figure S1 Forest plots of motor conduction velocity of the common peroneal nerve.

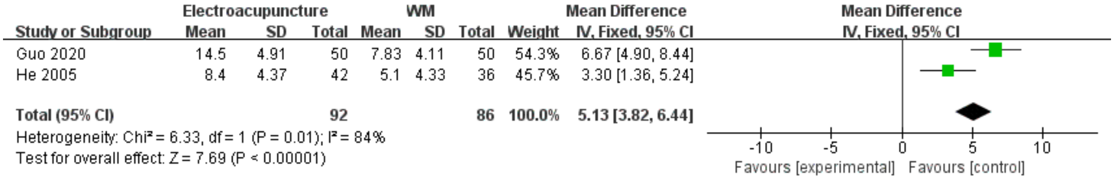

Supplementary Figure S1.1 Forest plot of motor conduction velocity of the common peroneal nerve in electroacupuncture versus WM.

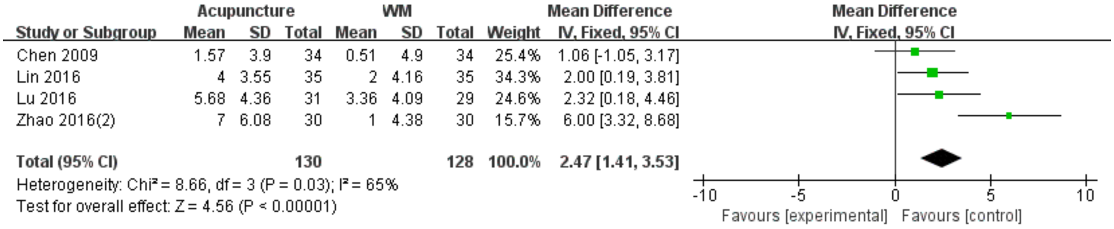

Supplementary Figure S1.2 Forest plot of motor conduction velocity of the common peroneal nerve in acupuncture versus WM.

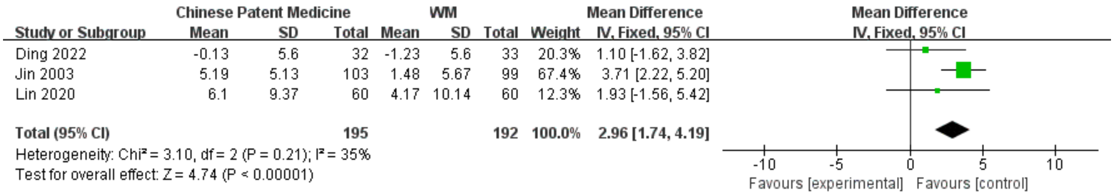

Supplementary Figure S1.3 Forest plot of motor conduction velocity of the common peroneal nerve in Chinese Patent Medicine versus WM.

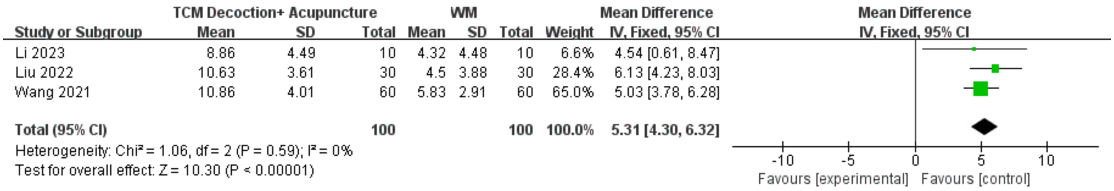

Supplementary Figure S1.4 Forest plot of motor conduction velocity of the common peroneal nerve in TCM Decoction+ Acupuncture versus WM.

Study or Subgroup	TCM Decoction+ Chinese Herbal Footbath Mean	SD	Total	WM Mean	SD	Total	Weight	Mean Difference IV, Fixed, 95% CI
Dai 2020(2)	32.13	8.57	85	22.21	3.24	85	22.2%	9.92 [7.97, 11.87]
Meng 2019	5.79	2.53	50	4.42	2.75	49	77.8%	1.37 [0.33, 2.41]
**Total (95% CI)**			**135**			**134**	**100.0%**	**3.27 [2.35, 4.19]**

Heterogeneity: Chi² = 57.56, df = 1 (P < 0.00001); I² = 98%
Test for overall effect: Z = 6.98 (P < 0.00001)

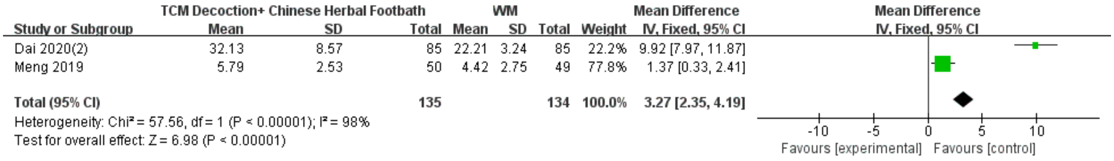

Supplementary Figure S1.5 Forest plot of motor conduction velocity of the common peroneal nerve in TCM Decoction+ Chinese Herbal Footbath versus WM.

Study or Subgroup	TCM decoction Mean	SD	Total	WM Mean	SD	Total	Weight	Mean Difference IV, Fixed, 95% CI
Zhi 2020	4.9	3.33	40	2.7	2.16	40	9.9%	2.20 [0.97, 3.43]
Yang 2021	6.04	3.92	25	1.35	3.94	24	3.1%	4.69 [2.49, 6.89]
Xu 2017	7.34	3.9	35	3.44	6.7	35	2.3%	3.90 [1.33, 6.47]
Wu 2021	5.1	5.39	60	3.2	4.49	60	4.8%	1.90 [0.12, 3.68]
Tang 2014	5.48	1.89	32	3.64	1.67	32	19.6%	1.84 [0.97, 2.71]
Song 2020	2.8	3.84	30	3.12	3.44	30	4.4%	-0.32 [-2.16, 1.52]
Ma 2022	5.46	3.54	48	2.38	9.53	48	1.8%	3.08 [0.20, 5.96]
Li 2021	6.4	5.79	50	3.3	5.79	50	2.9%	3.10 [0.83, 5.37]
Hu 2021	10.08	1.87	33	5.71	1.75	33	19.6%	4.37 [3.50, 5.24]
Ding 2016	12.9	4.32	40	6.5	4	40	4.5%	6.40 [4.58, 8.22]
Deng 2016	6.69	5.17	39	2.53	5.13	39	2.9%	4.16 [1.87, 6.45]
Dai 2020	6.89	4.39	53	3.8	4.35	53	5.4%	3.09 [1.43, 4.75]
Dai 2018	7.21	5.08	40	3.42	5.11	40	3.0%	3.79 [1.56, 6.02]
Cui 2022	10.85	2.53	61	5.93	2.88	59	15.9%	4.92 [3.95, 5.89]
**Total (95% CI)**			**586**			**583**	**100.0%**	**3.36 [2.97, 3.75]**

Heterogeneity: Chi² = 61.01, df = 13 (P < 0.00001); I² = 79%
Test for overall effect: Z = 17.02 (P < 0.00001)

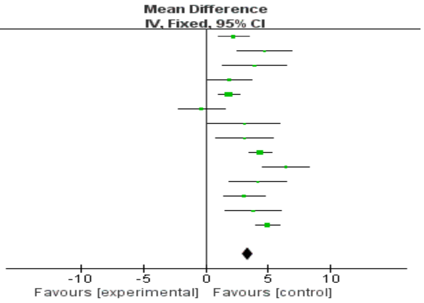

Supplementary Figure S1.6 Forest plot of motor conduction velocity of the common peroneal nerve in TCM Decoction versus WM.
