# Supplementary material for: Traditional Chinese medicine for diabetic peripheral neuropathy: a network meta-analysis
Source: Front Endocrinol (Lausanne). 2025 Aug 27;16:1596924. doi: 10.3389/fendo.2025.1596924 (PMC12420273; doi:10.3389/fendo.2025.1596924)
Supplement: Supplementary file 2 [file DataSheet2.pdf]

Supplementary Figure S2 Forest plots of sensory conduction velocity of the common peroneal nerve.

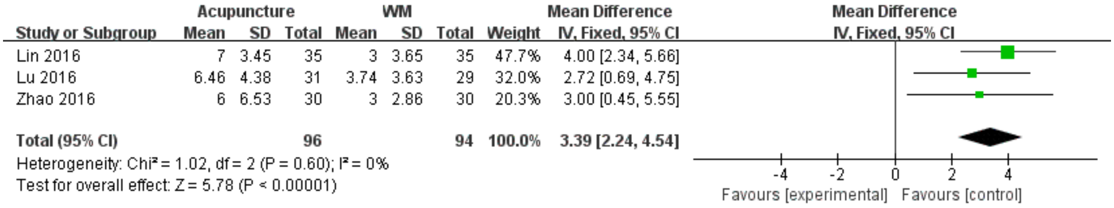

Figure S2.1 Forest plot of sensory conduction velocity of the common peroneal nerve in acupuncture versus WM.

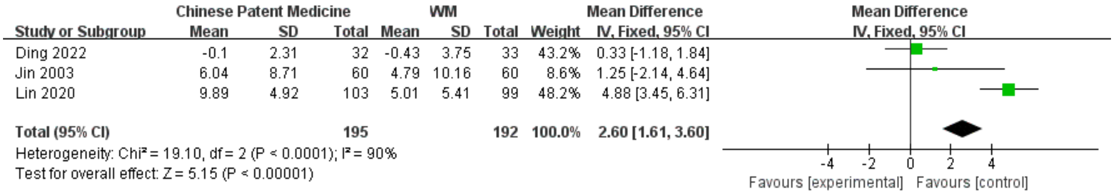

Figure S2.2 Forest plot of sensory conduction velocity of the common peroneal nerve in Chinese Patent Medicine versus WM.

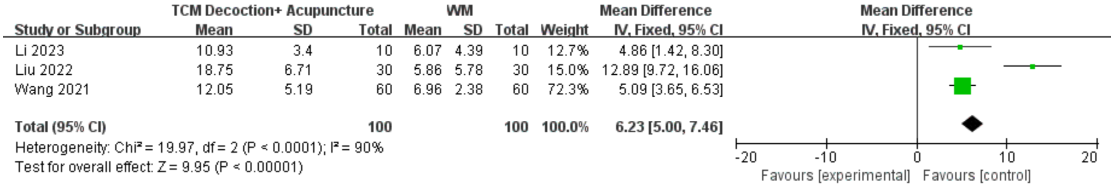

Figure S2.3 Forest plot of sensory conduction velocity of the common peroneal nerve in TCM Decoction+ Acupuncture versus WM.

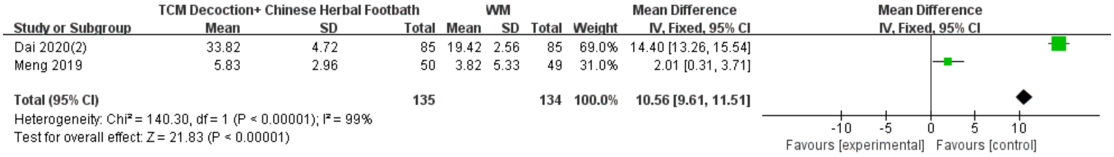

Figure S2.4 Forest plot of sensory conduction velocity of the common peroneal nerve in TCM Decoction+ Chinese Herbal Footbath versus WM.

Study or Subgroup	TCM decoction Mean	TCM decoction SD	TCM decoction Total	WM Mean	WM SD	WM Total	Weight	Mean Difference IV, Fixed, 95% CI
Cui 2022	11.36	1.97	61	6.04	2.15	59	20.9%	5.32 [4.58, 6.06]
Dai 2018	8.62	13.95	40	0.53	4.46	40	0.6%	8.09 [3.55, 12.63]
Deng 2016	8.49	5.41	39	3.09	5.43	39	2.0%	5.40 [2.99, 7.81]
Ding 2016	15.3	3.69	40	4.8	3.89	40	4.1%	10.50 [8.84, 12.16]
Hu 2021	8.46	1.92	33	4.47	1.82	33	14.0%	3.99 [3.09, 4.89]
Li 2021	4.67	5.19	50	2	5.34	50	2.7%	2.67 [0.61, 4.73]
Song 2020	3.2	3.37	30	3.5	3.01	30	4.4%	-0.30 [-1.92, 1.32]
Tang 2014	3.55	1.85	32	1.61	1.01	32	21.3%	1.94 [1.21, 2.67]
Xu 2017	6.66	4.68	35	3.67	2.09	35	3.9%	2.99 [1.29, 4.69]
Yang 2021	5.34	3.16	25	2.31	3.77	24	3.0%	3.03 [1.08, 4.98]
Zhi 2020	6.5	1.75	40	4.4	1.43	40	23.2%	2.10 [1.40, 2.80]
**Total (95% CI)**			**425**			**422**	**100.0%**	**3.42 [3.08, 3.76]**

Heterogeneity: Chi² = 154.04, df = 10 (P < 0.00001); I² = 94%
Test for overall effect: Z = 19.87 (P < 0.00001)

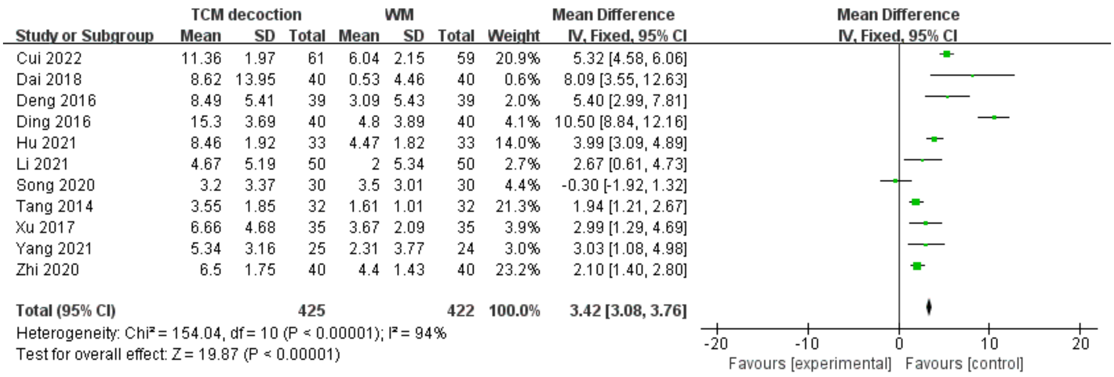

Figure S2.5 Forest plot of sensory conduction velocity of the common peroneal nerve in TCM Decoction versus WM.
