# Supplementary material for: Traditional Chinese medicine for diabetic peripheral neuropathy: a network meta-analysis
Source: Front Endocrinol (Lausanne). 2025 Aug 27;16:1596924. doi: 10.3389/fendo.2025.1596924 (PMC12420273; doi:10.3389/fendo.2025.1596924)
Supplement: Supplementary file 3 [file DataSheet3.pdf]

Supplementary Figure S3 Forest plots of motor conduction velocity of median nerve.

Study or Subgroup	Electroacupuncture Mean	SD	Total	WM Mean	SD	Total	Weight	Mean Difference IV, Fixed, 95% CI
Guo 2020	20.53	6.89	50	9.34	6.04	50	51.8%	11.19 [8.65, 13.73]
Xue 2004	8.4	5.97	34	5.9	5.08	34	48.2%	2.50 [-0.13, 5.13]
**Total (95% CI)**			**84**			**84**	**100.0%**	**7.00 [5.18, 8.83]**

Heterogeneity: Chi² = 21.66, df = 1 (P < 0.00001); I² = 95%
Test for overall effect: Z = 7.51 (P < 0.00001)

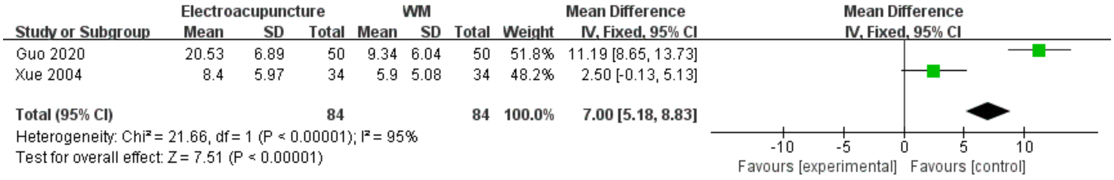

Figure S3.1 Forest plot of motor conduction velocity of median nerve of electroacupuncture versus WM.

Study or Subgroup	Acupuncture Mean	SD	Total	WM Mean	SD	Total	Weight	Mean Difference IV, Fixed, 95% CI
Chen 2009	0.51	5.03	34	0.01	5.43	34	19.7%	0.50 [-1.99, 2.99]
Lin 2016	4	3.25	35	2	3.46	35	49.4%	2.00 [0.43, 3.57]
Lu 2016	4.54	4.06	31	3.06	3.81	29	30.8%	1.48 [-0.51, 3.47]
**Total (95% CI)**			**100**			**98**	**100.0%**	**1.54 [0.44, 2.65]**

Heterogeneity: Chi² = 1.00, df = 2 (P = 0.61); I² = 0%
Test for overall effect: Z = 2.74 (P = 0.006)

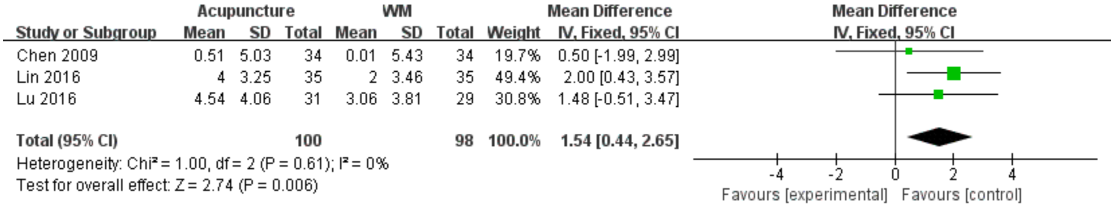

Figure S3.2 Forest plot of motor conduction velocity of median nerve of oacupuncture versus WM.

Study or Subgroup	Chinese Patent Medicine Mean	SD	Total	WM Mean	SD	Total	Weight	Mean Difference IV, Fixed, 95% CI
Ding 2022	-0.85	4.6	32	-8.45	9.16	33	8.5%	7.60 [4.09, 11.11]
Jin 2003	6.91	6.7	103	10.04	6.78	99	30.2%	-3.13 [-4.99, -1.27]
Lin 2020	7.27	9.55	60	8.47	8.99	60	9.5%	-1.20 [-4.52, 2.12]
Xiao 2018	11.2	4.21	66	3.6	4.12	66	51.8%	7.60 [6.18, 9.02]
**Total (95% CI)**			**261**			**258**	**100.0%**	**3.52 [2.50, 4.54]**

Heterogeneity: Chi² = 93.76, df = 3 (P < 0.00001); I² = 97%
Test for overall effect: Z = 6.75 (P < 0.00001)

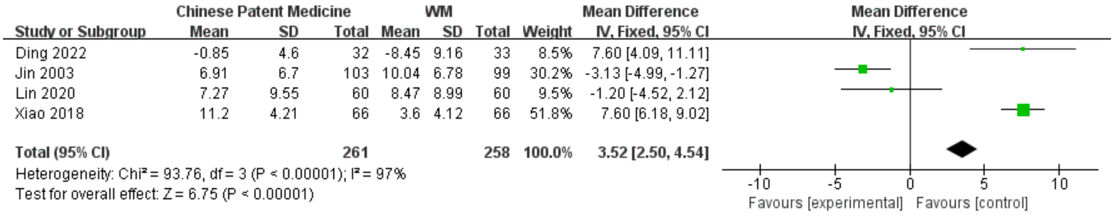

Figure S3.3 Forest plot of motor conduction velocity of median nerve of Chinese Patent Medicine versus WM.

Study or Subgroup	TCM Decoction+ Acupuncture Mean	SD	Total	WM Mean	SD	Total	Weight	Mean Difference IV, Fixed, 95% CI
Li 2023	12.82	5.59	10	6.12	5.57	10	4.9%	6.70 [1.81, 11.59]
Liu 2022	11.75	3.23	30	4.96	4.09	30	33.5%	6.79 [4.93, 8.65]
Wang 2021	11.75	4.05	60	5.55	3.62	60	61.6%	6.20 [4.83, 7.57]
**Total (95% CI)**			**100**			**100**	**100.0%**	**6.42 [5.34, 7.50]**

Heterogeneity: Chi² = 0.26, df = 2 (P = 0.88); I² = 0%
Test for overall effect: Z = 11.66 (P < 0.00001)

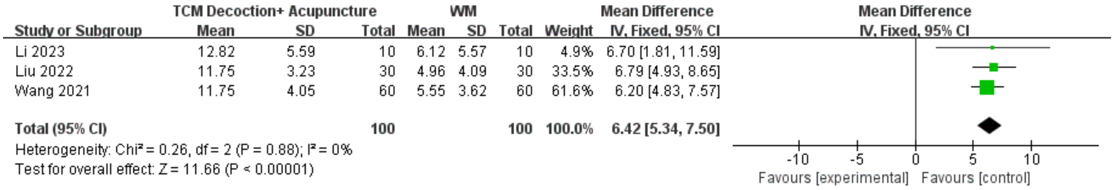

Figure S3.4 Forest plot of motor conduction velocity of median nerve of TCM Decoction+ Acupuncture versus WM.

Study or Subgroup	TCM decoction Mean	SD	Total	WM Mean	SD	Total	Weight	Mean Difference IV, Fixed, 95% CI
Cui 2022	9.84	3.24	61	4.38	2.29	59	15.4%	5.46 [4.46, 6.46]
Dai 2020	14.96	2.33	53	5.45	5.39	53	6.2%	9.51 [7.93, 11.09]
Hu 2021	9.15	1.74	33	3.44	1.69	33	22.6%	5.71 [4.88, 6.54]
Jiang 2021	9.73	5.21	46	2.12	4.06	46	4.2%	7.61 [5.70, 9.52]
Li 2021	5.64	7.09	50	2.68	7.15	50	2.0%	2.96 [0.17, 5.75]
Ma 2022	3.43	4.17	48	1.83	4.44	48	5.2%	1.60 [-0.12, 3.32]
Song 2020	0.4	2.69	30	3.4	2.85	30	7.9%	-3.00 [-4.40, -1.60]
Tang 2014	5.35	1.96	32	2.69	1.32	32	23.1%	2.66 [1.84, 3.48]
Wu 2021	5.4	5.39	60	4.5	5.35	60	4.2%	0.90 [-1.02, 2.82]
Yang 2021	7.64	3.17	25	3.44	5.37	24	2.5%	4.20 [1.72, 6.68]
Zhi 2020	8.6	3.43	40	5.6	3.47	40	6.8%	3.00 [1.49, 4.51]
**Total (95% CI)**			**478**			**475**	**100.0%**	**3.91 [3.51, 4.30]**

Heterogeneity: Chi² = 210.45, df = 10 (P < 0.00001); I² = 95%
Test for overall effect: Z = 19.48 (P < 0.00001)

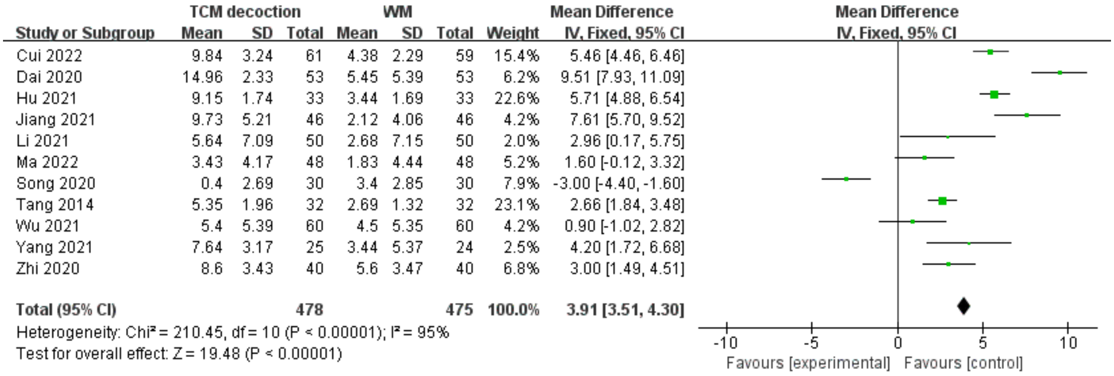

Figure S3.5 Forest plot of motor conduction velocity of median nerve of TCM Decoction versus WM.
