# Supplementary material for: Traditional Chinese medicine for diabetic peripheral neuropathy: a network meta-analysis
Source: Front Endocrinol (Lausanne). 2025 Aug 27;16:1596924. doi: 10.3389/fendo.2025.1596924 (PMC12420273; doi:10.3389/fendo.2025.1596924)
Supplement: Supplementary file 4 [file DataSheet4.pdf]

Supplementary Figure S4 Forest plots of sensory conduction velocity of median nerve.

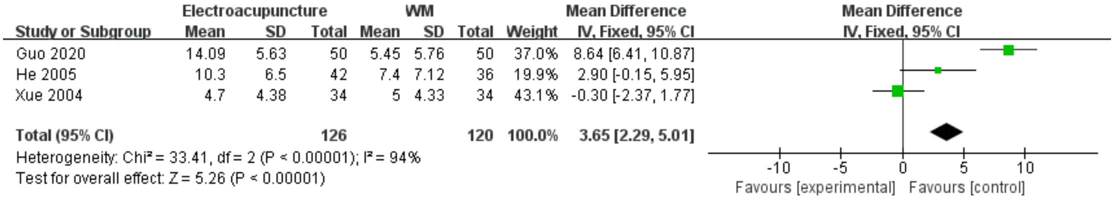

Supplementary Figure S4.1 Forest plot of sensory conduction velocity of median nerve of electroacupuncture versus WM.

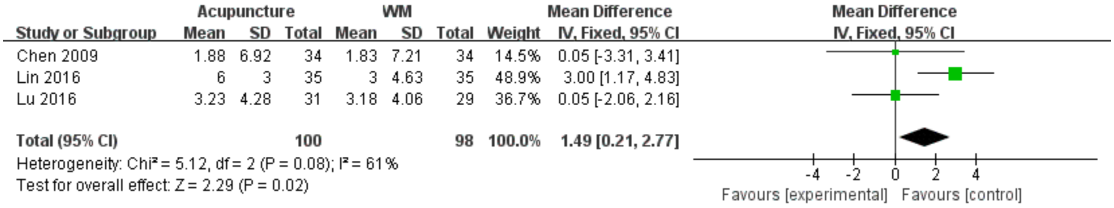

Supplementary Figure S4.2 Forest plot of sensory conduction velocity of median nerve of acupuncture versus WM.

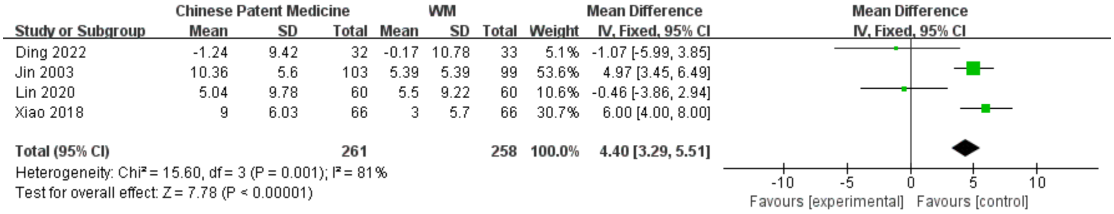

Supplementary Figure S4.3 Forest plot of sensory conduction velocity of median nerve of Chinese Patent Medicine versus WM.

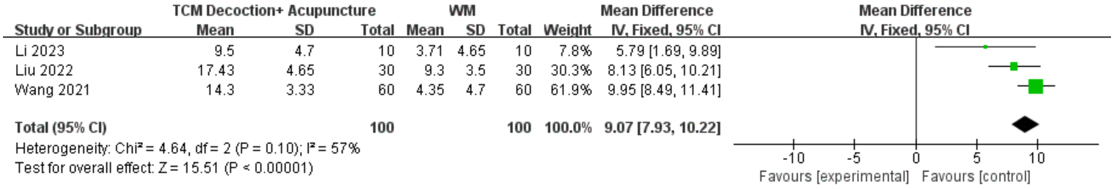

Supplementary Figure S4.4 Forest plot of sensory conduction velocity of median nerve of TCM Decoction+ Acupuncture versus WM.

	TCM decoction			WM				Mean Difference
**Study or Subgroup**	**Mean**	**SD**	**Total**	**Mean**	**SD**	**Total**	**Weight**	**IV, Fixed, 95% CI**
Cui 2022	9.07	3.44	61	3.61	2.68	59	10.0%	5.46 [4.36, 6.56]
Dai 2020	15.98	3.07	53	10.82	3.07	53	8.9%	5.16 [3.99, 6.33]
Hu 2021	9.05	1.67	33	5.31	1.58	33	19.7%	3.74 [2.96, 4.52]
Jiang 2021	7.83	3.24	46	4.56	3.52	46	6.3%	3.27 [1.89, 4.65]
Li 2021	3.88	6.99	50	2.93	7.2	50	1.6%	0.95 [-1.83, 3.73]
Ma 2022	4.39	6.01	48	1.02	6.99	48	1.8%	3.37 [0.76, 5.98]
Song 2020	2.6	3.73	30	2.7	3.17	30	3.9%	-0.10 [-1.85, 1.65]
Tang 2014	4.59	1.49	32	1.96	1.35	32	24.9%	2.63 [1.93, 3.33]
Wu 2021	4.2	5.82	60	3	4.57	60	3.4%	1.20 [-0.67, 3.07]
Yang 2021	5.06	3.24	25	1.32	4.24	24	2.7%	3.74 [1.62, 5.86]
Zhi 2020	3.5	2.33	40	4.4	1.43	40	16.8%	-0.90 [-1.75, -0.05]
**Total (95% CI)**			**478**			**475**	**100.0%**	**2.66 [2.31, 3.01]**

Heterogeneity: Chi² = 132.88, df = 10 (P < 0.00001); I² = 92%

Test for overall effect: Z = 14.99 (P < 0.00001)

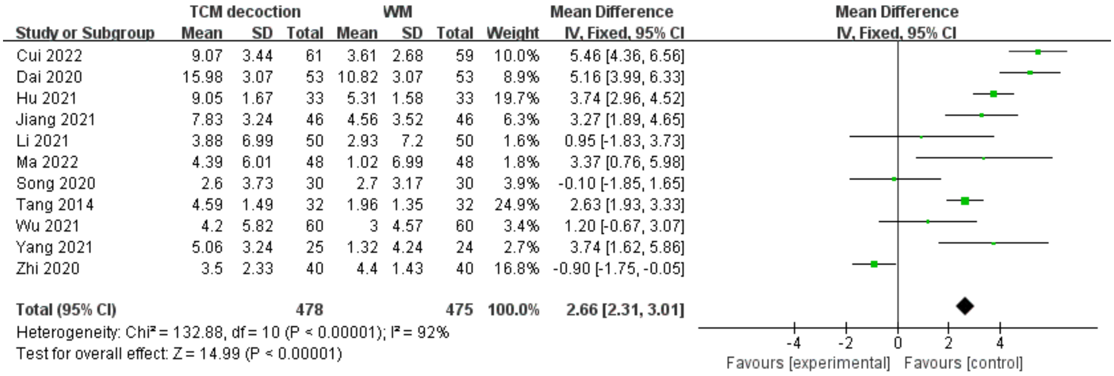

Supplementary Figure S4.5 Forest plot of sensory conduction velocity of median nerve of TCM Decoction versus WM.
