# Supplementary material for: Traditional Chinese medicine for diabetic peripheral neuropathy: a network meta-analysis
Source: Front Endocrinol (Lausanne). 2025 Aug 27;16:1596924. doi: 10.3389/fendo.2025.1596924 (PMC12420273; doi:10.3389/fendo.2025.1596924)
Supplement: Supplementary file 5 [file DataSheet5.pdf]

Supplementary Figure S5 Forest plots of FBG.

	Chinese Patent Medicine			WM				Mean Difference
**Study or Subgroup**	**Mean**	**SD**	**Total**	**Mean**	**SD**	**Total**	**Weight**	**IV, Fixed, 95% CI**
Ding 2022	0.06	3.39	32	1.19	3.71	32	5.5%	-1.13 [-2.87, 0.61]
Jin 2021	-1.33	3.86	33	0.45	3.25	34	5.7%	-1.78 [-3.49, -0.07]
Xiao 2018	-2.6	0.88	66	-1.1	1.57	66	88.8%	-1.50 [-1.93, -1.07]
**Total (95% CI)**			**131**			**132**	**100.0%**	**-1.50 [-1.90, -1.09]**

Heterogeneity: Chi² = 0.28, df = 2 (P = 0.87); I² = 0%
Test for overall effect: Z = 7.17 (P < 0.00001)

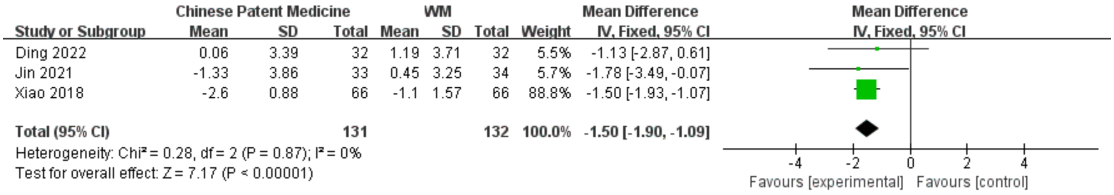

Supplementary Figure S5.1 Forest plot of FBG of Chinese Patent Medicine versus WM.

	TCM Decoction+ Acupuncture			WM				Mean Difference
**Study or Subgroup**	**Mean**	**SD**	**Total**	**Mean**	**SD**	**Total**	**Weight**	**IV, Fixed, 95% CI**
Wang 2021	-5.6	2.36	60	-2.24	2.68	60	10.9%	-3.36 [-4.26, -2.46]
Wu 2024	-0.68	0.72	35	-0.31	0.68	35	82.9%	-0.37 [-0.70, -0.04]
Yu 2018	-2.8	2.45	36	-1.3	2.68	34	6.1%	-1.50 [-2.70, -0.30]
**Total (95% CI)**			**131**			**129**	**100.0%**	**-0.77 [-1.07, -0.47]**

Heterogeneity: Chi² = 38.68, df = 2 (P < 0.00001); I² = 95%
Test for overall effect: Z = 5.03 (P < 0.00001)

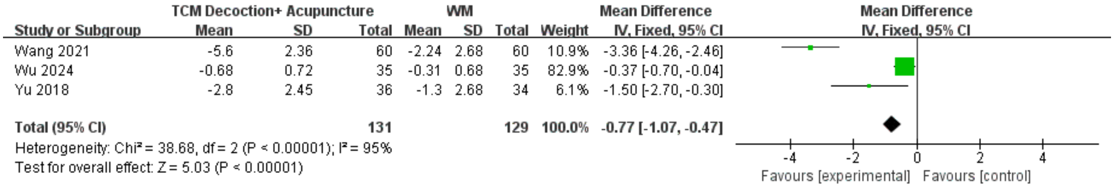

Supplementary Figure S5.2 Forest plot of FBG of TCM Decoction+ Acupuncture versus WM.

	TCM decoction			WM				Mean Difference
**Study or Subgroup**	**Mean**	**SD**	**Total**	**Mean**	**SD**	**Total**	**Weight**	**IV, Fixed, 95% CI**
Cui 2022	-1.99	1.41	61	-1.08	1.24	59	8.0%	-0.91 [-1.38, -0.44]
Hu 2019	-6.2	1.12	45	-2.18	1.69	45	5.1%	-4.02 [-4.61, -3.43]
Jiang 2021	-2.09	1.95	46	-1.3	1.98	46	2.8%	-0.79 [-1.59, 0.01]
Ma 2022	-1.32	1.33	48	-1.2	1.46	48	5.7%	-0.12 [-0.68, 0.44]
Song 2020	-2.06	3.08	30	-0.37	3.04	30	0.7%	-1.69 [-3.24, -0.14]
Zhi 2020	-0.19	0.36	38	-0.01	0.31	37	77.7%	-0.18 [-0.33, -0.03]
**Total (95% CI)**			**268**			**265**	**100.0%**	**-0.46 [-0.59, -0.33]**

Heterogeneity: Chi² = 159.74, df = 5 (P < 0.00001); I² = 97%
Test for overall effect: Z = 6.72 (P < 0.00001)

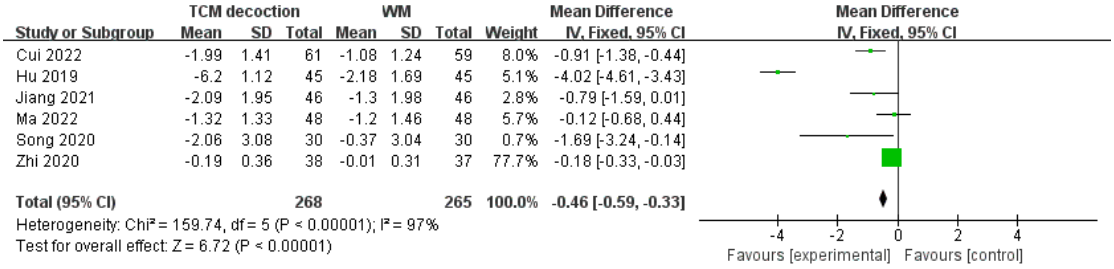

Supplementary Figure S5.3 Forest plot of FBG of TCM Decoction versus WM.
