# Supplementary material for: Traditional Chinese medicine for diabetic peripheral neuropathy: a network meta-analysis
Source: Front Endocrinol (Lausanne). 2025 Aug 27;16:1596924. doi: 10.3389/fendo.2025.1596924 (PMC12420273; doi:10.3389/fendo.2025.1596924)
Supplement: Supplementary file 6 [file DataSheet6.pdf]

Supplementary Figure S6 Forest plots of 2hPG.

Study or Subgroup	Chinese Patent Medicine Mean	SD	Total	WM Mean	SD	Total	Weight	Mean Difference IV, Fixed, 95% CI
Jin 2021	-0.42	2.16	33	-0.08	3.03	34	19.5%	-0.34 [-1.60, 0.92]
Xiao 2018	-4.2	1.76	66	-2.1	1.86	66	80.5%	-2.10 [-2.72, -1.48]
**Total (95% CI)**			**99**			**100**	**100.0%**	**-1.76 [-2.31, -1.20]**

Heterogeneity: Chi² = 6.06, df = 1 (P = 0.01); I² = 84%
Test for overall effect: Z = 6.21 (P < 0.00001)

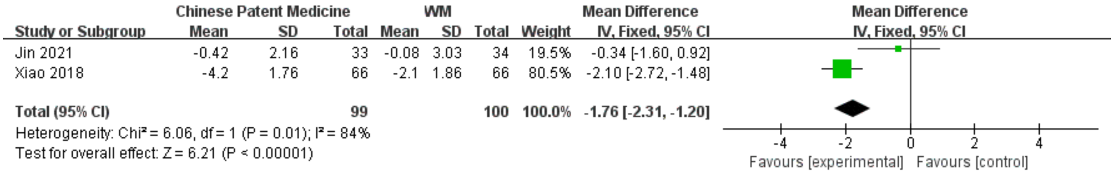

Supplementary Figure S6.1 Forest plot of 2hPG of Chinese Patent Medicine versus WM.

Study or Subgroup	TCM Decoction+ Acupuncture Mean	SD	Total	WM Mean	SD	Total	Weight	Mean Difference IV, Fixed, 95% CI
Wang 2021	-5.77	3.07	60	-5.16	4.32	60	10.3%	-0.61 [-1.95, 0.73]
Wu 2024	-0.67	1	35	-0.22	1.05	35	80.5%	-0.45 [-0.93, 0.03]
Yu 2018	-3	2.94	36	-1.9	3.13	34	9.2%	-1.10 [-2.52, 0.32]
**Total (95% CI)**			**131**			**129**	**100.0%**	**-0.53 [-0.96, -0.10]**

Heterogeneity: Chi² = 0.73, df = 2 (P = 0.69); I² = 0%
Test for overall effect: Z = 2.39 (P = 0.02)

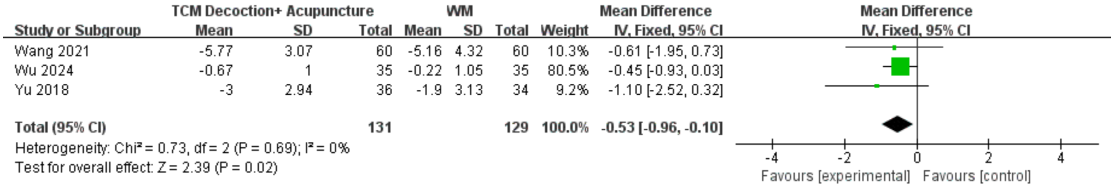

Supplementary Figure S6.2 Forest plot of 2hPG of TCM Decoction+ Acupuncture versus WM.

Study or Subgroup	TCM decoction Mean	SD	Total	WM Mean	SD	Total	Weight	Mean Difference IV, Fixed, 95% CI
Cui 2022	-2.28	3.51	61	-1.25	2.32	59	4.8%	-1.03 [-2.09, 0.03]
Hu 2019	-5.53	1.64	45	-3.82	1.47	45	13.1%	-1.71 [-2.35, -1.07]
Jiang 2021	-3.31	2.5	46	-2.48	2.51	46	5.2%	-0.83 [-1.85, 0.19]
Ma 2022	-1.5	2.07	48	-1.04	2.03	48	8.1%	-0.46 [-1.28, 0.36]
Song 2020	-0.64	3.9	30	-1.06	3.3	30	1.6%	0.42 [-1.41, 2.25]
Zhi 2020	-0.23	0.69	38	-0.04	0.56	37	67.2%	-0.19 [-0.47, 0.09]
**Total (95% CI)**			**268**			**265**	**100.0%**	**-0.47 [-0.71, -0.24]**

Heterogeneity: Chi² = 20.45, df = 5 (P = 0.001); I² = 76%
Test for overall effect: Z = 3.99 (P < 0.0001)

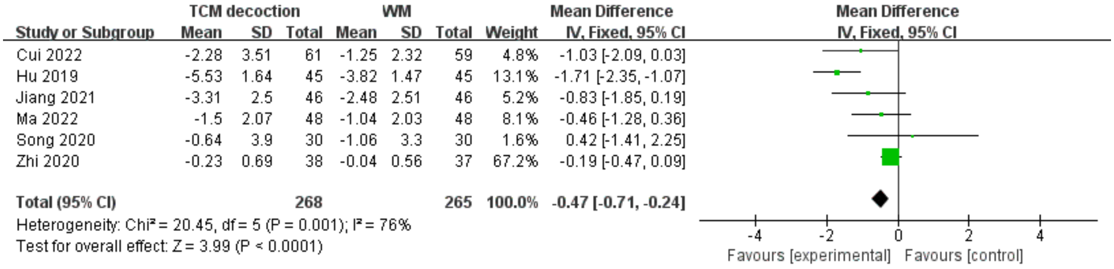

Supplementary Figure S6.3 Forest plot of 2hPG of TCM Decoction versus WM.
