# Supplementary material for: Traditional Chinese medicine for diabetic peripheral neuropathy: a network meta-analysis
Source: Front Endocrinol (Lausanne). 2025 Aug 27;16:1596924. doi: 10.3389/fendo.2025.1596924 (PMC12420273; doi:10.3389/fendo.2025.1596924)
Supplement: Supplementary file 7 [file DataSheet7.pdf]

Supplementary Figure S7 Forest plot of TCSS.

	Chinese Patent Medicine			WM				Mean Difference
Study or Subgroup	Mean	SD	Total	Mean	SD	Total	Weight	IV, Fixed, 95% CI
Jin 2021	-2.03	2.53	33	-1.26	2.86	34	45.2%	-0.77 [-2.06, 0.52]
Lin 2020	-3	3.51	60	-3.1	3.03	60	54.8%	0.10 [-1.07, 1.27]
**Total (95% CI)**			**93**			**94**	**100.0%**	**-0.29 [-1.16, 0.58]**

Heterogeneity: Chi² = 0.95, df = 1 (P = 0.33); I² = 0%
Test for overall effect: Z = 0.66 (P = 0.51)

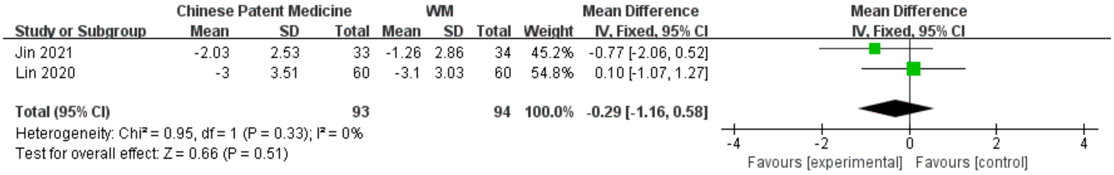

Supplementary Figure S7.1 Forest plot of TCSS of Chinese Patent Medicine versus WM.

	TCM Decoction+ Acupuncture			WM				Mean Difference
Study or Subgroup	Mean	SD	Total	Mean	SD	Total	Weight	IV, Fixed, 95% CI
Wang 2021	-5.5	2.51	60	-3.89	2.54	60	67.4%	-1.61 [-2.51, -0.71]
Wu 2024	-3.05	2.78	35	-1.81	2.76	35	32.6%	-1.24 [-2.54, 0.06]
**Total (95% CI)**			**95**			**95**	**100.0%**	**-1.49 [-2.23, -0.75]**

Heterogeneity: Chi² = 0.21, df = 1 (P = 0.65); I² = 0%
Test for overall effect: Z = 3.94 (P < 0.0001)

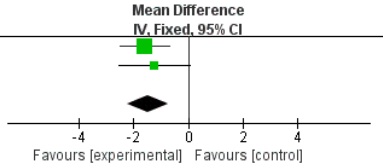

Supplementary Figure S7.2 Forest plot of TCSS of TCM Decoction+ Acupuncture versus WM.

	TCM decoction			WM				Mean Difference
Study or Subgroup	Mean	SD	Total	Mean	SD	Total	Weight	IV, Fixed, 95% CI
Dong 2017	-5.5	2.31	40	-4.45	2.06	40	17.1%	-1.05 [-2.01, -0.09]
Fan 2019	-4.2	1.98	45	-1.96	1.91	45	24.3%	-2.24 [-3.04, -1.44]
Jian 2020	-8.5	1.67	50	-4.28	2.01	50	29.9%	-4.22 [-4.94, -3.50]
Ma 2022	-2.66	2.55	48	-2.5	2.17	48	17.5%	-0.16 [-1.11, 0.79]
Wang 2017	-5.56	2.49	37	-4.11	2.69	37	11.2%	-1.45 [-2.63, -0.27]
**Total (95% CI)**			**220**			**220**	**100.0%**	**-2.18 [-2.57, -1.78]**

Heterogeneity: Chi² = 54.76, df = 4 (P < 0.00001); I² = 93%
Test for overall effect: Z = 10.77 (P < 0.00001)

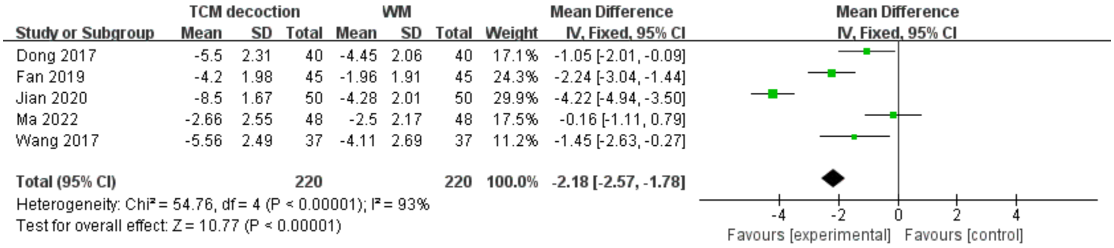

Supplementary Figure S7.3 Forest plot of TCSS of TCM Decoction versus WM.
