# Supplementary material for: Traditional Chinese medicine for diabetic peripheral neuropathy: a network meta-analysis
Source: Front Endocrinol (Lausanne). 2025 Aug 27;16:1596924. doi: 10.3389/fendo.2025.1596924 (PMC12420273; doi:10.3389/fendo.2025.1596924)
Supplement: Supplementary file 8 [file DataSheet8.pdf]

Supplementary Figure S8 Forest plots of total effective rate

	TCM decoction		WM			Odds Ratio
Study or Subgroup	Events	Total	Events	Total	Weight	M-H, Fixed, 95% CI
Bai 2021	30	31	22	30	0.6%	10.91 [1.27, 93.69]
Cao 2018	31	36	24	35	2.8%	2.84 [0.87, 9.28]
Chen 2012	40	42	30	42	1.2%	8.00 [1.66, 38.45]
Cheng 2017	40	42	32	42	1.3%	6.25 [1.28, 30.58]
Cheng 2020	34	40	26	40	3.3%	3.05 [1.03, 9.02]
Chou 2017	47	50	35	50	1.8%	6.71 [1.80, 25.00]
Cui 2022	51	61	35	59	4.9%	3.50 [1.49, 8.21]
Dai 2018	34	40	25	40	3.1%	3.40 [1.16, 10.00]
Dai 2019	33	35	26	35	1.2%	5.71 [1.13, 28.75]
Deng 2016	36	39	30	39	1.9%	3.60 [0.89, 14.51]
Deng 2017	41	45	33	45	2.5%	3.73 [1.10, 12.64]
Ding 2016	34	40	27	40	3.4%	2.73 [0.92, 8.13]
Dong 2017	37	40	34	40	2.1%	2.18 [0.50, 9.39]
Fan 2019	42	45	35	45	2.0%	4.00 [1.02, 15.68]
Gong 2017	58	60	50	60	1.4%	5.80 [1.21, 27.73]
Hu 2019	31	33	24	33	1.2%	5.81 [1.15, 29.44]
Hu 2021	55	60	36	60	2.5%	7.33 [2.56, 20.98]
Huang 2015	49	50	37	50	0.6%	17.22 [2.15, 137.57]
Jian 2020	40	46	32	46	3.5%	2.92 [1.01, 8.45]
Jiang 2021	49	50	44	50	0.7%	6.68 [0.77, 57.70]
Li 2021	30	40	14	40	2.9%	5.57 [2.12, 14.65]
Liu 2016	34	36	28	36	1.3%	4.86 [0.95, 24.75]
Liu 2017	45	50	37	50	3.1%	3.16 [1.03, 9.69]
Ma 2016	27	29	18	28	1.1%	7.50 [1.47, 38.32]
Ma 2016(2)	27	33	19	32	2.9%	3.08 [0.99, 9.55]
Mo 2016	28	30	9	30	0.5%	32.67 [6.38, 167.28]
Pan 2002	41	45	32	45	2.4%	4.16 [1.24, 14.00]
Pang 2020	36	39	27	39	1.7%	5.33 [1.37, 20.78]
Qin 2016	38	45	33	45	4.3%	1.97 [0.70, 5.60]
Shi 2015	39	45	29	45	3.2%	3.59 [1.25, 10.29]
Shi 2019	39	40	32	40	0.7%	9.75 [1.16, 82.11]
Shi 2020	21	30	12	30	3.0%	3.50 [1.20, 10.20]
Song 2020	33	40	25	40	3.7%	2.83 [1.00, 7.98]
Sun 2018	30	32	21	32	1.1%	7.86 [1.58, 39.17]
Tang 2014	35	37	29	37	1.3%	4.83 [0.95, 24.54]
Wang 2017	56	60	45	60	2.5%	4.67 [1.45, 15.05]
Wu 2021	33	35	25	35	1.2%	6.60 [1.33, 32.84]
Xu 2017	28	30	23	30	1.3%	4.26 [0.81, 22.53]
Xu 2019	34	35	19	34	0.5%	26.84 [3.28, 219.34]
Xue 2015	22	25	15	24	1.5%	4.40 [1.02, 18.99]
Yang 2021	26	29	20	30	1.7%	4.33 [1.05, 17.85]
Zhang 2015	32	34	24	34	1.2%	6.67 [1.34, 33.28]
Zhang 2017	29	30	24	30	0.7%	7.25 [0.82, 64.46]
Zhang 2017(2)	34	35	28	35	0.7%	8.50 [0.99, 73.28]
Zhang 2019	34	36	28	36	1.3%	4.86 [0.95, 24.75]
Zhao 2016	45	50	32	50	2.7%	5.06 [1.70, 15.05]
Zhao 2019	42	47	30	46	2.7%	4.48 [1.48, 13.57]
Zhao 2022	36	40	29	40	2.4%	3.41 [0.98, 11.85]
Zhi 2020	31	35	24	35	2.3%	3.55 [1.01, 12.55]
Zou 2016	45	49	31	49	2.1%	6.53 [2.02, 21.17]
**Total (95% CI)**		**2026**		**2018**	**100.0%**	**4.66 [3.88, 5.59]**
Total events	1842		1399			

Heterogeneity: $Chi^2 = 25.20$, df = 49 (P = 1.00); $I^2 = 0\%$

Test for overall effect: Z = 16.52 (P < 0.00001)

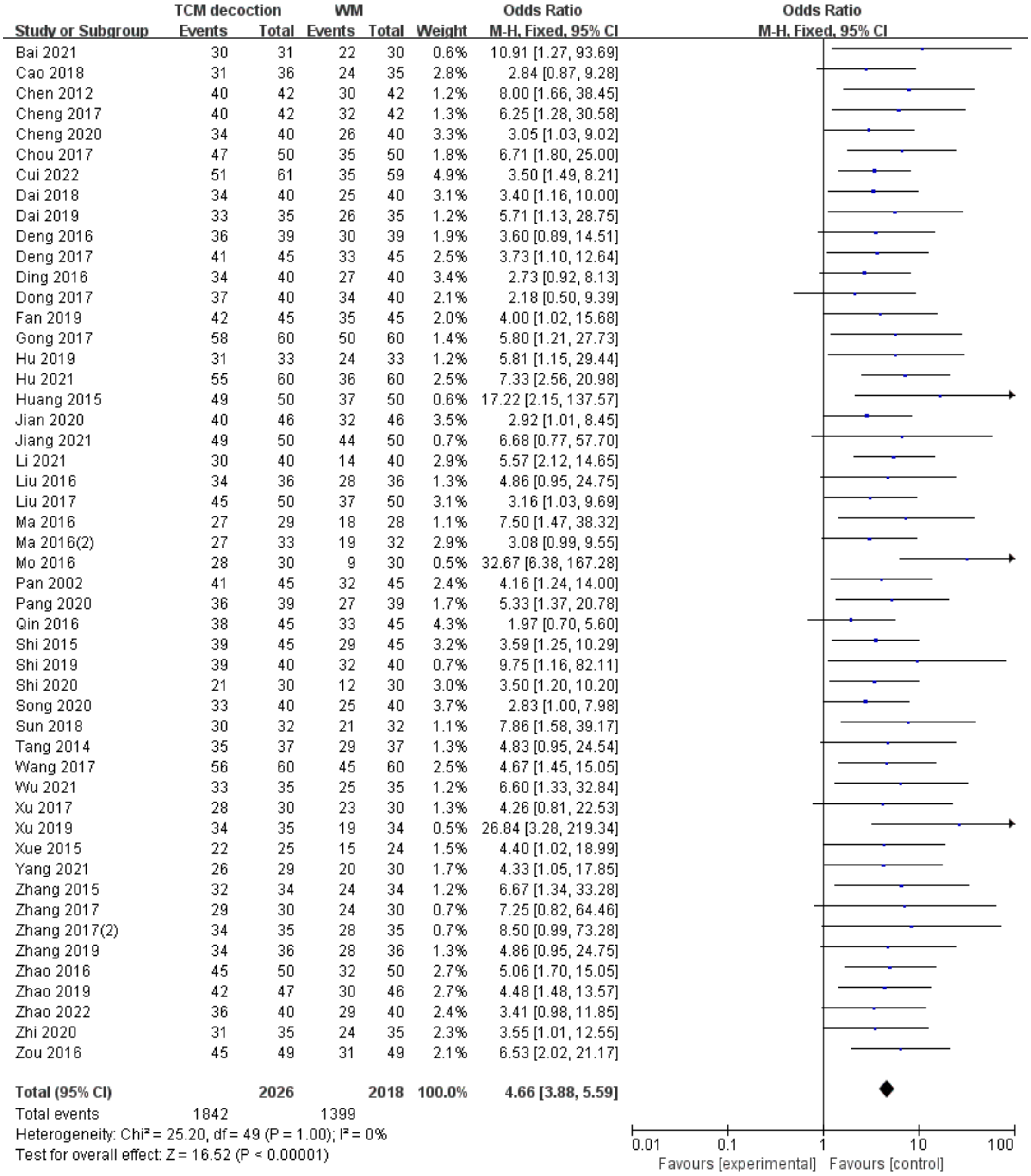

Supplementary Figure S8.1 Forest plot of total effective rate of TCM Decoction versus WM.

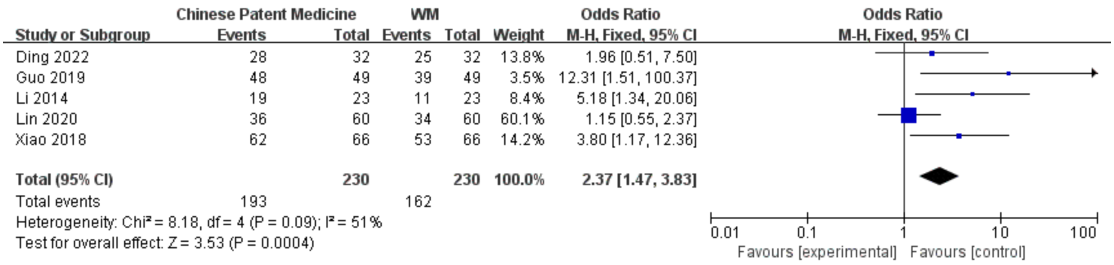

Supplementary Figure S8.2 Forest plot of total effective rate of Chinese Patent Medicine versus WM.

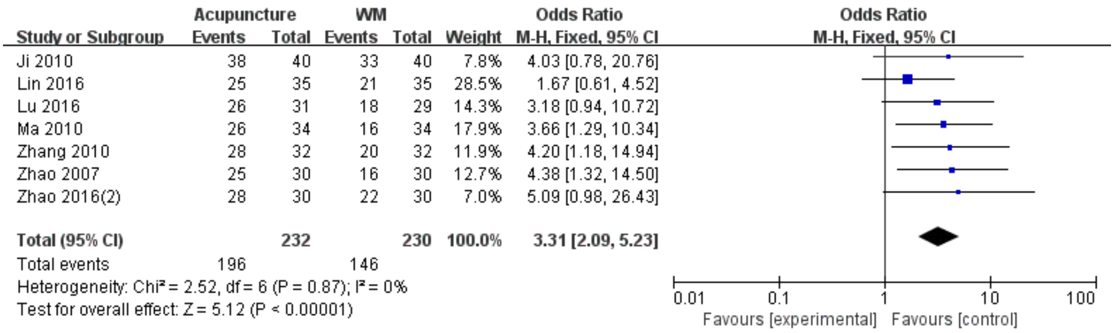

Supplementary Figure S8.3 Forest plot of total effective rate of acupuncture versus WM.

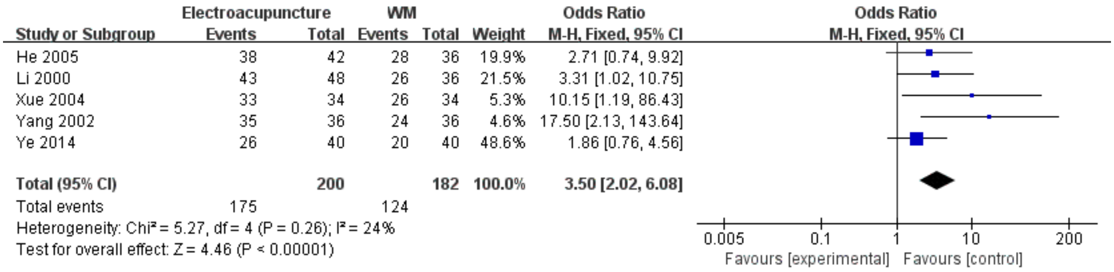

Supplementary Figure S8.4 Forest plot of total effective rate of electroacupuncture versus WM.

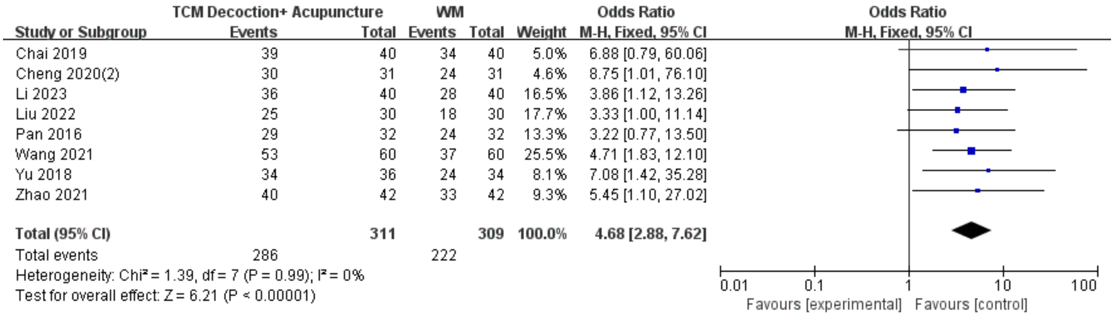

Supplementary Figure S8.5 Forest plot of total effective rate of TCM Decoction+ Acupuncture versus WM.

Study or Subgroup	TCM Decoction+ Chinese Herbal Footbath Events	Total	WM Events	Total	Weight	Odds Ratio M-H, Fixed, 95% CI
Chen 2020	50	52	40	52	5.9%	7.50 [1.59, 35.46]
Dai 2020(2)	84	85	73	85	3.3%	13.81 [1.75, 108.77]
Deng 2019	45	45	40	45	1.7%	12.36 [0.66, 230.48]
Fan 2018	375	400	280	400	67.1%	6.43 [4.07, 10.16]
Meng 2019	46	50	37	49	11.5%	3.73 [1.11, 12.53]
Zhang 2019(2)	28	32	22	32	10.5%	3.18 [0.88, 11.52]
**Total (95% CI)**		**664**		**663**	**100.0%**	**6.18 [4.23, 9.04]**
Total events	628		492			

Heterogeneity: Chi² = 2.58, df = 5 (P = 0.76); I² = 0%
Test for overall effect: Z = 9.39 (P < 0.00001)

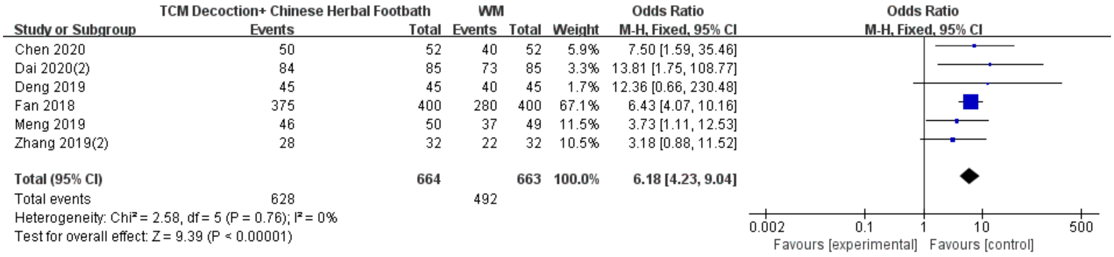

Supplementary Figure S8.6 Forest plot of total effective rate of TCM Decoction+ Chinese Herbal Footbath versus WM.

Study or Subgroup	TCM Decoction+ Chinese Herbal Fumigation Events	Total	WM Events	Total	Weight	Odds Ratio M-H, Fixed, 95% CI
Han 2021	38	41	31	41	50.4%	4.09 [1.03, 16.15]
Liu 2020	34	35	28	35	17.8%	8.50 [0.99, 73.28]
Yang 2018	33	35	25	35	31.8%	6.60 [1.33, 32.84]
**Total (95% CI)**		**111**		**111**	**100.0%**	**5.67 [2.23, 14.42]**
Total events	105		84			

Heterogeneity: Chi² = 0.39, df = 2 (P = 0.82); I² = 0%
Test for overall effect: Z = 3.64 (P = 0.0003)

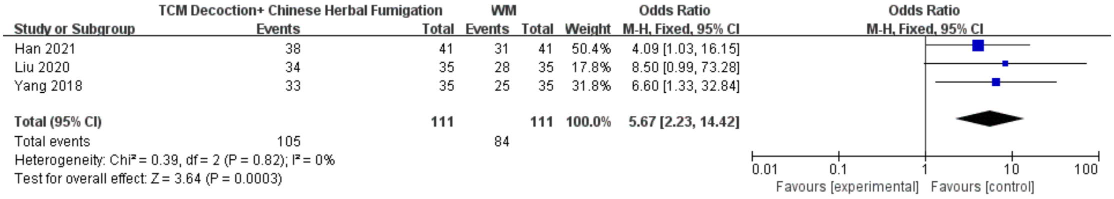

Supplementary Figure S8.7 Forest plot of total effective rate of TCM Decoction+ Chinese Medicine Fumigation versus WM.

Study or Subgroup	Acupoint Injection+ Chinese Herbal Fumigation Events	Total	WM Events	Total	Weight	Odds Ratio M-H, Fixed, 95% CI
Chen 2017	29	33	18	33	16.9%	6.04 [1.73, 21.09]
Zhang 2016	38	45	25	45	30.0%	4.34 [1.60, 11.78]
Zhang 2017(3)	74	85	50	75	53.1%	3.36 [1.52, 7.45]
**Total (95% CI)**		**163**		**153**	**100.0%**	**4.11 [2.36, 7.16]**
Total events	141		93			

Heterogeneity: Chi² = 0.62, df = 2 (P = 0.73); I² = 0%
Test for overall effect: Z = 4.99 (P < 0.00001)

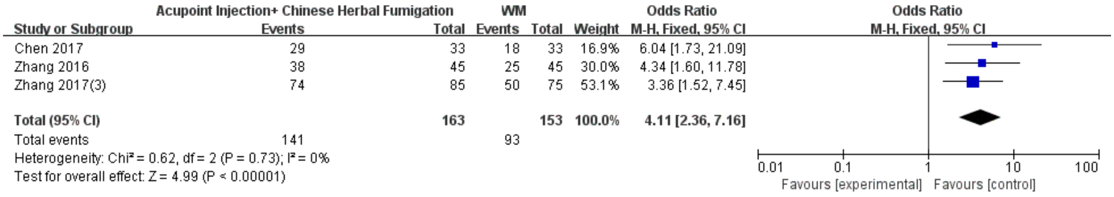

Supplementary Figure S8.8 Forest plot of total effective rate of Acupoint Injection+ Chinese Medicine Fumigation versus WM.
