# Supplementary material for: Traditional Chinese medicine for diabetic peripheral neuropathy: a network meta-analysis
Source: Front Endocrinol (Lausanne). 2025 Aug 27;16:1596924. doi: 10.3389/fendo.2025.1596924 (PMC12420273; doi:10.3389/fendo.2025.1596924)
Supplement: Supplementary file 9 [file DataSheet9.pdf]

Supplementary Figure S9

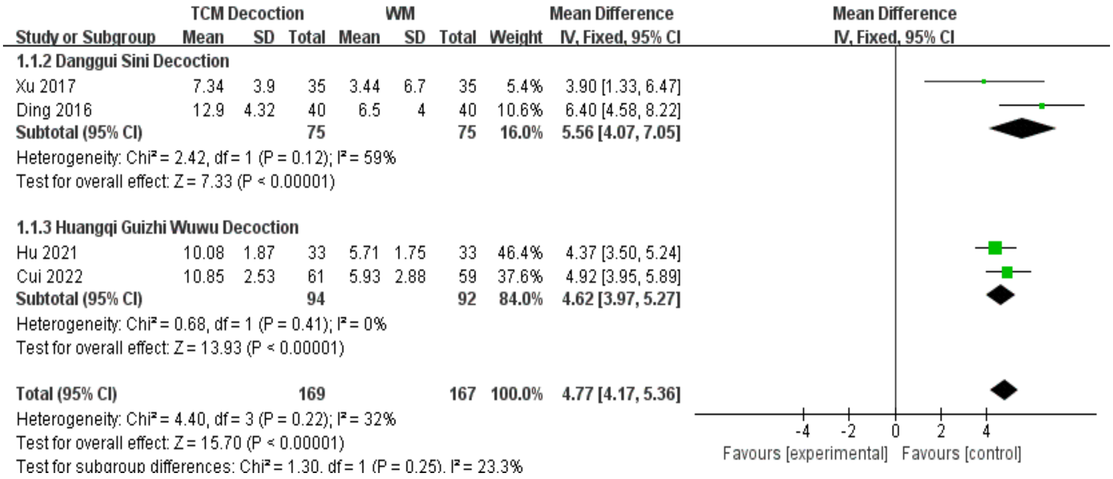

Supplementary Figure S9.1 Forest plot of the subgroup analyses for motor conduction velocity of common peroneal nerve in TCM Decoction versus WM with different prescriptions.

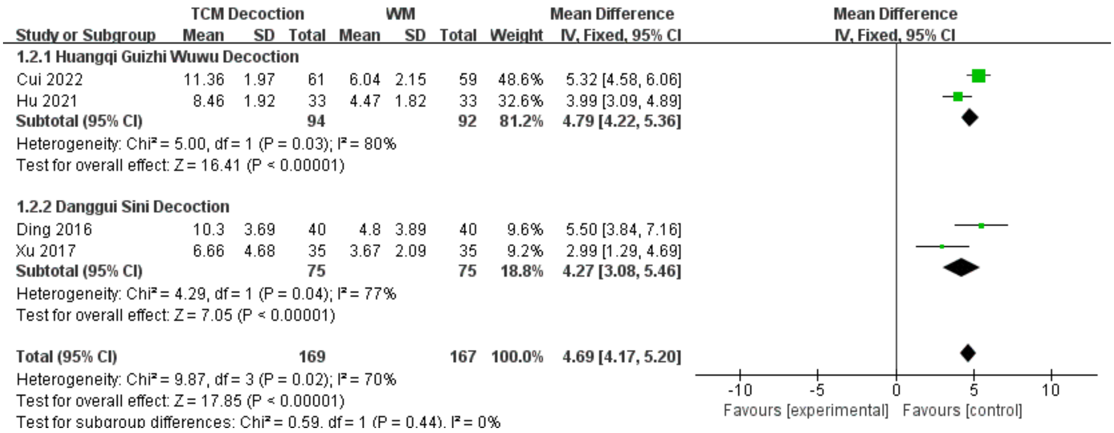

Supplementary Figure S9.2 Forest plot of the subgroup analyses for sensory conduction velocity of common peroneal nerve in TCM Decoction versus WM with different prescriptions.

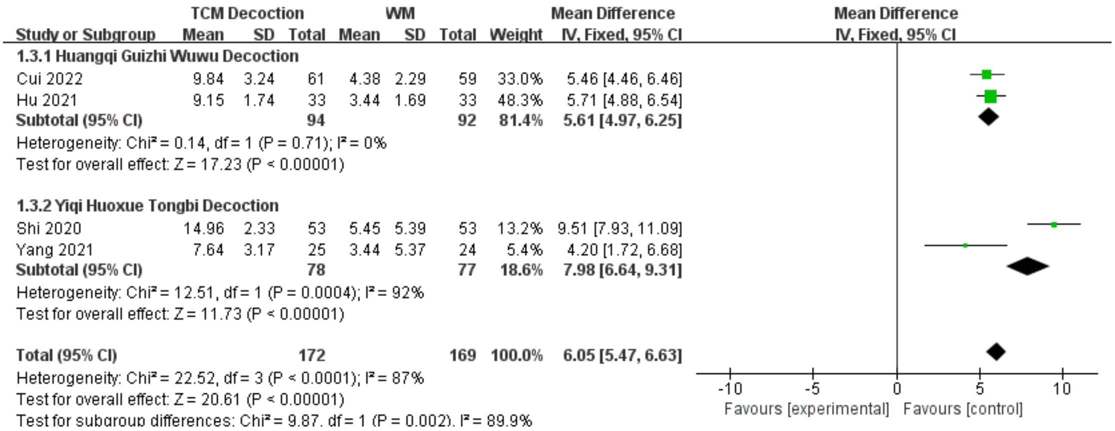

Supplementary Figure S9.3 Forest plot of the subgroup analyses for motor conduction velocity of median nerve in TCM Decoction versus WM with different prescriptions.

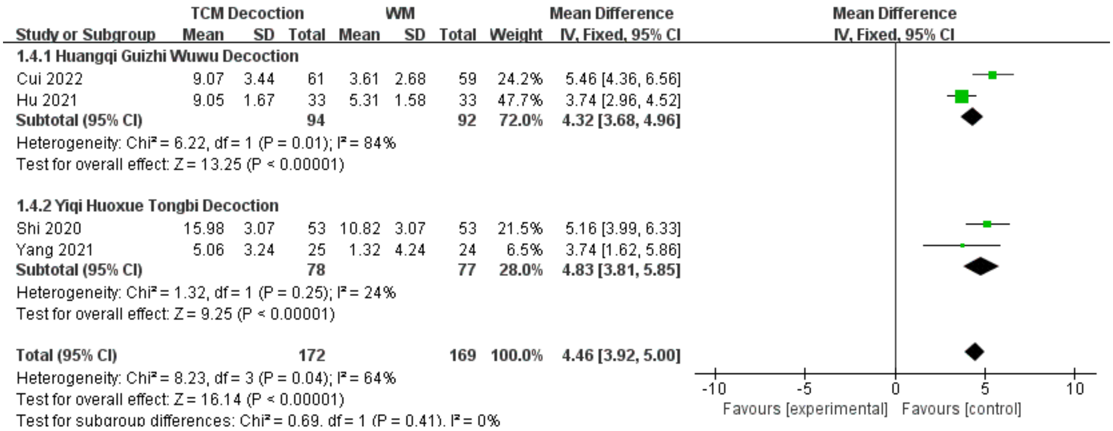

Supplementary Figure S9.4 Forest plot of the subgroup analyses for sensory conduction velocity of median nerve in TCM Decoction versus WM with different prescriptions.

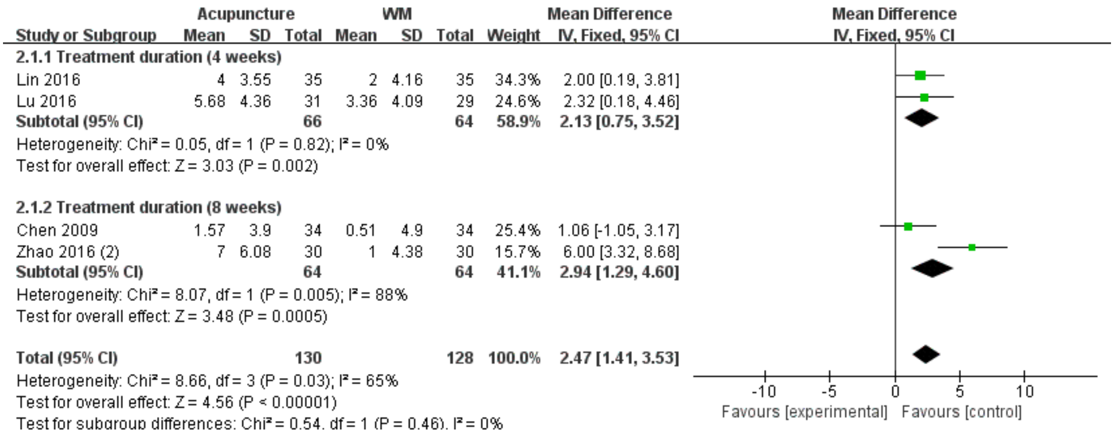

Supplementary Figure S9.5 Forest plot of the subgroup analyses for motor conduction velocity of common peroneal nerve in acupuncture versus WM with different treatment duration.

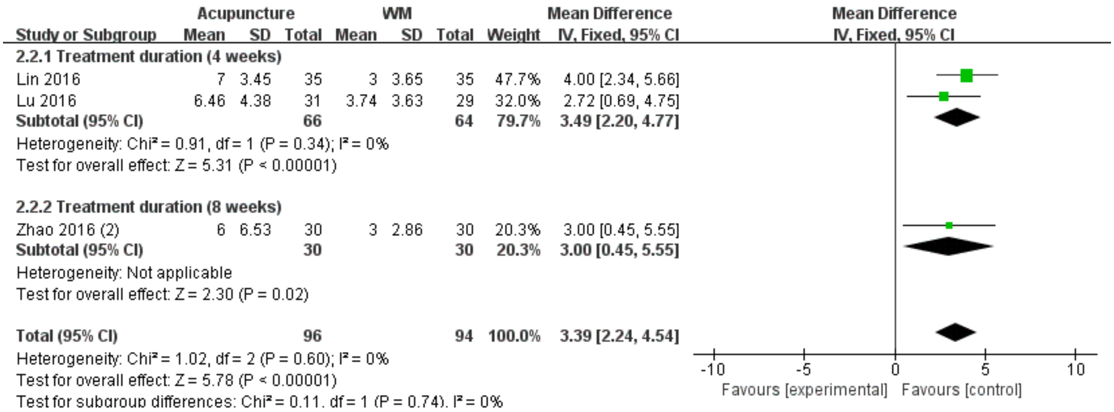

Supplementary Figure S9.6 Forest plot of the subgroup analyses for sensory conduction velocity of common peroneal nerve in acupuncture versus WM with different treatment duration.
