# Supplementary material for: Traditional Chinese medicine for diabetic peripheral neuropathy: a network meta-analysis
Source: Front Endocrinol (Lausanne). 2025 Aug 27;16:1596924. doi: 10.3389/fendo.2025.1596924 (PMC12420273; doi:10.3389/fendo.2025.1596924)
Supplement: Supplementary file 10 [file DataSheet10.pdf]

Supplementary Figure S10

Egger's test

Std_Eff	Coefficient	Std. err.	t	P>|t|	[95% conf.	interval]
slope	3.039754	1.006351	3.02	0.006	.9711694	5.108339
bias	.6399448	1.235188	0.52	0.609	-1.89902	3.17891

Supplementary Figure S10.1 Egger test for motor conduction velocity of common peroneal nerve.

Egger's test

Std_Eff	Coefficient	Std. err.	t	P>|t|	[95% conf.	interval]
slope	3.335673	1.635005	2.04	0.055	-.0748876	6.746233
bias	1.340576	2.399485	0.56	0.583	-3.664661	6.345813

Supplementary Figure S10.2 Egger test for sensory conduction velocity of common peroneal nerve.

Egger's test

Std_Eff	Coefficient	Std. err.	t	P>|t|	[95% conf.	interval]
slope	4.530057	1.727016	2.62	0.016	.9385298	8.121584
bias	-.7397444	2.163971	-0.34	0.736	-5.239969	3.760481

Supplementary Figure S10.3 Egger test for motor conduction velocity of median nerve.

Egger's test

Std_Eff	Coefficient	Std. err.	t	P>|t|	[95% conf.	interval]
slope	2.845917	1.233409	2.31	0.031	.2879828	5.403851
bias	.5559864	1.636376	0.34	0.737	-2.83765	3.949623

Supplementary Figure S10.4 Egger test for sensory conduction velocity of median nerve.
