# Supplementary material for: Traditional Chinese medicine for diabetic peripheral neuropathy: a network meta-analysis
Source: Front Endocrinol (Lausanne). 2025 Aug 27;16:1596924. doi: 10.3389/fendo.2025.1596924 (PMC12420273; doi:10.3389/fendo.2025.1596924)
Supplement: Supplementary file 11 [file Supplementaryfile1.pdf]

PRISMA 2020 Checklist

Section and Topic	Item #	Checklist item	Location where item is reported
**TITLE**			
Title	1	Identify the report as a systematic review.	Traditional Chinese Medicine for diabetic peripheral neuropathy: A network meta-analysis
**ABSTRACT**			
Abstract	2	See the PRISMA 2020 for Abstracts checklist.	Abstract:<br>Background:<br>Diabetic peripheral neuropathy (DPN) is a common complication of diabetes mellitus, characterized by high morbidity and significant disability. Traditional Chinese medicine (TCM) has shown potential in relieving symptoms and improving neurological function through multi-targeted mechanisms; however, the efficacy and safety of different TCM therapies have yet to be systematically evaluated.<br>Objective: This study aims to provide evidence-based medicine for treating DPN with TCM therapy by network meta-analysis (NMA).<br>Methods: This study comprehensively searched nine databases constructed up to November 2024. The quality and evidence of the included RCTs were assessed using the risk of bias assessment tool and GRADE pro, and pairwise meta-analysis and NMA were performed using Revman, Stata, and R Studio. The results showed that 95 RCTs involving 8194 patients were included, containing 9 TCM therapies.<br>Results: TCM Decoration + Acupuncture ranked highest in improving the motor conduction velocity of the common peroneal nerve (SUCRA=0.81), followed by TCM Decoction + Chinese Herbal Footbath (SUCRA = 0.80), electroacupuncture (SUCRA = 0.75). Regarding the sensory conduction velocity of the common peroneal nerve, TCM Decoration + Chinese Herbal Foot (SUCRA=0.87) ranked first, followed by TCM Decoction + Acupuncture (SUCRA = 0.83), and TCM Decoction (SUCRA = 0.51). Electroacupuncture (SUCRA=0.83) ranks first in improving median nerve motor conduction velocity, followed by TCM Decoction + Acupuncture (SUCRA = 0.82), TCM Decoction (SUCRA = 0.55). TCM Decoration + Acupuncture (SUCRA=0.98) ranks first in improving the sensory conduction velocity of the median nerve, followed by electroacupuncture (SUCRA=0.72), and Chinese Patent Medicine (SUCRA=0.51). TCM Decoration + Chinese Herbal Footbath (SUCRA=0.85) ranked first in improving overall clinical symptoms of DPN.<br>Conclusion: The effectiveness and safety of traditional Chinese medicine therapy in treating DPN have been preliminarily verified. In clinical practice, conservative clinical stratification selection can be made based on the results of this study and the actual situation. In addition, due to the limited quality of the included studies, larger sample sizes and high-quality research are still needed.
**INTRODUCTION**			
Rationale	3	Describe the rationale for the review in the context of existing knowledge.	Diabetic peripheral neuropathy (DPN) is one of the common complications of type 1 and type 2 diabetes mellitus. DPN is characterized by peripheral nerve involvement in the lower limbs, with symmetrical numbness, discomfort, and pain in the lower limbs, which often starts from the feet, spreads upward to the calves, and later spreads to the upper limbs. Clinical signs include profound sensory deficits, such as decreased or absent Achilles tendon reflex, knee tendon reflex, and positional and vibration senses, which often lead to gait and balance dysfunction. In recent years, interventions in TCM for treating DPN have been classified into three main categories: single-drug extracts, herbal decoctions, and Chinese patent medicines. External therapies include acupuncture, Chinese herbal footbath, acupoint injections, and combinations of these methods. Although these therapies show promise in treating DPN, it remains unclear whether there are significant differences among the various therapeutic approaches and which intervention may be the most effective.
Objectives	4	Provide an explicit statement of the objective(s) or question(s) the review addresses.	To evaluate the efficacy and safety of TCM therapies for DPN and identify the optimal treatment regimen, we will conduct direct and indirect comparisons of TCM protocols utilized for DPN thus far. We aim to provide evidence-based medical information to support the use of TCM therapies in treating DPN.

Section and Topic	Item #	Checklist item	Location where item is reported
**METHODS**			
Eligibility criteria	5	Specify the inclusion and exclusion criteria for the review and how studies were grouped for the syntheses.	Eligibility criteria<br>We only analyzed randomized controlled trials of TCM therapies for treating DPN. RCTs were required to have the following inclusion criteria: (1) the diagnosis of patients with DPN should be precise (no restriction on age, gender, and duration of the disease); (2) the interventions in the treatment group were TCM therapies, including internal treatments of TCM (TCM Decoction, Chinese Patent Medicine, single herbs, etc.), external treatments of TCM (Chinese Herbal Footbath, acupuncture, electroacupuncture, etc.), or their combination; (3) The control group's interventions were Western medicines only.<br>We will exclude the following studies: (1) duplicate studies, (2) reviews, animal experiments, protocols, conference papers, dissertations, and case reports; (3) studies that were not formally published; and (4) studies with incomplete data.
Information sources	6	Specify all databases, registers, websites, organisations, reference lists and other sources searched or consulted to identify studies. Specify the date when each source was last searched or consulted.	Information sources<br>We searched the following databases for RCTs of TCM therapies for the treatment of DPN (searches were performed until November 2024): PubMed, Cochrane Library, Embase, Web of Science, Medline, China National Knowledge Infrastructure (CNKI), Wanfang Data Knowledge Service Platform (Wanfang), VIP Database (VIP), and China Biology Medicine disc, (CBM disc). A detailed search strategy was completed on November 20, 2024, and some of the search strategies are documented in Supplementary Table S2.
Search strategy	7	Present the full search strategies for all databases, registers and websites, including any filters and limits used.	Supplementary Table S1.
Selection process	8	Specify the methods used to decide whether a study met the inclusion criteria of the review, including how many reviewers screened each record and each report retrieved, whether they worked independently, and if applicable, details of automation tools used in the process.	Selection of studies and data collection<br>Three reviewers completed this process. Two reviewers (Yubo Gong and Xiaogang Hao) first excluded the duplicate studies used and then skimmed the titles and abstracts of the remaining studies. After excluding some studies that did not fit the topic, the full text of the remaining studies was carefully read to ensure data availability. Disagreements during the process will be addressed through discussion to reach a consensus. If these disagreements cannot be resolved, the third reviewer (Xuefeng Li) will make a decision after conducting an independent review.
Data collection process	9	Specify the methods used to collect data from reports, including how many reviewers collected data from each report, whether they worked independently, any processes for obtaining or confirming data from study investigators, and if applicable, details of automation tools used in the process.	For the studies that met the inclusion criteria, we will extract the first author, year of publication, disease duration, age, sample size, intervention, and outcome indicators.
Data items	10a	List and define all outcomes for which data were sought. Specify whether all results that were compatible with each outcome domain in each study were sought (e.g. for all measures, time points, analyses), and if not, the methods used to decide which results to collect.	We will use electromyography (EMG) results as the primary outcome indicator, and secondary outcome indicators may also include the Toronto Clinical Scoring System (TCSS), blood glucose, glycosylated hemoglobin (HbA1c), and total effective rate.

PRISMA 2020 Checklist

Section and Topic	Item #	Checklist item	Location where item is reported
	10b	List and define all other variables for which data were sought (e.g. participant and intervention characteristics, funding sources). Describe any assumptions made about any missing or unclear information.	For the studies that met the inclusion criteria, we will extract the first author, year of publication, disease duration, age, sample size, intervention, and outcome indicators.
Study risk of bias assessment	11	Specify the methods used to assess risk of bias in the included studies, including details of the tool(s) used, how many reviewers assessed each study and whether they worked independently, and if applicable, details of automation tools used in the process.	Risk of bias assessment<br>We used the risk of bias assessment tool recommended by the Cochrane Handbook to evaluate the risk of bias in the final included studies. The tool evaluated seven aspects of random generation: Random sequence generation, allocation concealment, blinding of patients and personnel, blinding of outcome assessment, incomplete outcome data, selective reporting, and other biases in the studies. The assessment of risk of bias will be reported as low risk, high risk, and unclear (Supplementary Table S3). Two reviewers (Ting Pan and Xue Zhou) will complete the assessment and discuss disagreements. If it is not resolved, a third reviewer (Siyi Wang) will assess and finalize it.
Effect measures	12	Specify for each outcome the effect measure(s) (e.g. risk ratio, mean difference) used in the synthesis or presentation of results.	First, pairwise meta-analysis was performed using Review Manager 5.4 software. The OR (odds ratio) and MD (mean difference) values were used to analyze dichotomous and continuous variables. Cochran's I-square (I^2) was used to determine the heterogeneity.
Synthesis methods	13a	Describe the processes used to decide which studies were eligible for each synthesis (e.g. tabulating the study intervention characteristics and comparing against the planned groups for each synthesis (item #5)).	If $I^2 < 50$ %, it indicates no significant statistical heterogeneity among the studies, and a fixed-effect model should be adopted. Otherwise, a random-effects model will be applied.
	13b	Describe any methods required to prepare the data for presentation or synthesis, such as handling of missing summary statistics, or data conversions.	The fact that an intervention has a higher SUCRA value means that it has a higher likelihood of being relatively superior among the interventions compared, but whether that superiority reaches a clinically meaningful threshold needs to be judged in conjunction with the effect size estimates (e.g., risk ratios, mean differences, etc.) and their 95% confidence intervals.
	13c	Describe any methods used to tabulate or visually display results of individual studies and syntheses.	A table of two-by-two comparisons will be generated for the final results, and the surface under the cumulative ranking curve (SUCRA) values will be used to rank the interventions. SUCRA values reflect the relative rank order between interventions and do not directly indicate the magnitude of effect sizes or the clinical importance of differences.
	13d	Describe any methods used to synthesize results and provide a rationale for the choice(s). If meta-analysis was performed, describe the model(s), method(s) to identify the presence and extent of statistical heterogeneity, and software package(s) used.	For network meta-analysis (NMA), we used Stata 15.0 and RStudio software. Stata 15.0 was used to generate network evidence plots and funnel plots and conduct a sensitivity analysis. The "mvmeta" package in Stata was utilized to create network evidence plots, visually illustrating the relationships between different interventions. In these plots, each point represents an intervention, and the size of the node indicates the sample size. A line connecting two nodes signifies a direct comparison between the two interventions. In contrast, the thickness of the line reflects the sample size for those interventions that have a direct comparison. When there is a closed loop, the node-splitting method is required to assess whether the results are inconsistent. Funnel plots were used for publication bias, while Egger's test was used for validation to prove the absence of publication bias when P > 0.05. The data were analyzed using the "gemtc" and "JAGs-4.3.1" packages in RStudio, and the consistency model was fitted based on the Markov chain Monte Carlo method (MCMC) framework to fit the consistency model. The consistency model was used for testing when P > 0.05, and vice versa, using the inconsistency model. The consistency model was used for testing when P > 0.05, and vice versa, using the inconsistency model. Binary variables were analyzed using OR values as effect sizes, continuous variables were analyzed using MD, and the

Section and Topic	Item #	Checklist item	Location where item is reported
			95% CI (Confidence interval) value of the effect sizes was calculated, with a 95% CI that did not contain one considered statistically significant.
	13e	Describe any methods used to explore possible causes of heterogeneity among study results (e.g. subgroup analysis, meta-regression).	The consistency model was used for testing when P > 0.05, and vice versa, using the inconsistency model. The consistency model was used for testing when P > 0.05, and vice versa, using the inconsistency model. Binary variables were analyzed using OR values as effect sizes, continuous variables were analyzed using MD, and the 95% CI (Confidence interval) value of the effect sizes was calculated, with a 95% CI that did not contain one considered statistically significant.
	13f	Describe any sensitivity analyses conducted to assess robustness of the synthesized results.	For network meta-analysis (NMA), we used Stata 15.0 and RStudio software. Stata 15.0 was used to generate network evidence plots and funnel plots and conduct a sensitivity analysis.
Reporting bias assessment	14	Describe any methods used to assess risk of bias due to missing results in a synthesis (arising from reporting biases).	Supplementary Table S3.
Certainty assessment	15	Describe any methods used to assess certainty (or confidence) in the body of evidence for an outcome. (GRADE)	Certainty of the Evidence Assessment<br>We utilized the GRADE system, as recommended by the BMJ, to evaluate the quality of evidence. The quality of evidence may be compromised in five ways: study limitations, inconsistency, indirectness, imprecision, and risk of bias. We used GRADE Pro version 3.6.1 to create a table outlining the levels of evidence.
**RESULTS**			
Study selection	16a	Describe the results of the search and selection process, from the number of records identified in the search to the number of studies included in the review, ideally using a flow diagram.	A total of 3279 studies were retrieved through the predefined search strategies, of which 1945 were duplicates. After the duplicates were eliminated, the remaining 1334 studies were browsed for titles and abstracts, and 770 were excluded in this step. Finally, the remaining 564 studies were scrutinized in full text, and 469 studies that did not meet the inclusion criteria were excluded. After the screening process, 95 RCTs were finally included; the complete screening process is shown in Figure 1.
	16b	Cite studies that might appear to meet the inclusion criteria, but which were excluded, and explain why they were excluded.	Figure 1.
Study characteristics	17	Cite each included study and present its characteristics.	The 95 RCTs incorporated within this study were all characterized by a two-arm trial design, encompassing 8,194 participants. The interventions encompassed TCM Decoction, Chinese Patent Medicine, acupuncture, electroacupuncture, TCM Decoction combined with acupuncture, TCM Decoction combined with Chinese Herbal Footbath, TCM Decoction combined with Chinese Medicine Fumigation, acupoint injections combined with Chinese Medicine Fumigation, and Western medicines, either oral or injectable. Of these RCTs, 87 (7833 participants) evaluated the total effective rate; 8 (550 participants) reported the adverse events; 87 (2461 participants) measured the motor conduction velocity of the common peroneal nerve, 23 (1893 participants) determined the sensory conduction velocity of the common peroneal nerve, 23 (2038 participants) evaluated the motor conduction velocity of the median nerve, 24 (2116 participants) assessed the sensory conduction velocity of the median nerve, 9 (817 participants) gauged the TCSS, 12 (1056 participants) scrutinized fasting blood glucose (FBG) levels, and 11 (992 participants) evaluated two-hour post-load plasma glucose (2hPG). Detailed information on the included studies is recorded in Table 1.
Risk of bias in studies	18	Present assessments of risk of bias for each included study.	Overall, the risk of bias of the RCTs included in this study ranged from low- risk to high. Regarding randomization, about 17.89% (n = 17) of the RCTs were assessed as low risk using a randomized table of numbers. In contrast, the other studies were assessed as an unclear risk for only mentioning randomization without specifying the exact method. 89.47% (n = 85) of the RCTs were assessed as low risk for adequately reporting on the concealment of

PRISMA 2020 Checklist

Section and Topic	Item #	Checklist item	Location where item is reported
			the allocation scheme; in terms of blinding, 98.95% of the studies were assessed as high risk due to specificity of treatment programs; for blinding of outcome assessment, 74.73% (n = 71) were assessed as low risk; 2.11% (n = 2) selectively reported on the outcome indicators mentioned in the text (but with justification), and were therefore assessed as unclear risk; all RCTs included in the present study were not found to be at risk of cause other biases in risk.
Results of individual studies	19	For all outcomes, present, for each study: (a) summary statistics for each group (where appropriate) and (b) an effect estimate and its precision (e.g. confidence/credible interval), ideally using structured tables or plots.	Table 1.
Results of syntheses	20a	For each synthesis, briefly summarise the characteristics and risk of bias among contributing studies.	Figure 2.
	20b	Present results of all statistical syntheses conducted. If meta-analysis was done, present for each the summary estimate and its precision (e.g. confidence/credible interval) and measures of statistical heterogeneity. If comparing groups, describe the direction of the effect.	Tables 2-25.
	20c	Present results of all investigations of possible causes of heterogeneity among study results.	Supplementary Figure S9.
	20d	Present results of all sensitivity analyses conducted to assess the robustness of the synthesized results.	Figure 4.
Reporting biases	21	Present assessments of risk of bias due to missing results (arising from reporting biases) for each synthesis assessed.	The funnel plot indicated the possibility of a small sample effect, so an Egger test was also conducted to verify the existence of publication bias further. The results of the Egger test are shown in the Supplementary Figure S10. The Egger test showed that $P > 0.05$, indicating that there was no publication bias. (Figure 5)
Certainty of evidence	22	Present assessments of certainty (or confidence) in the body of evidence for each outcome assessed.	The quality of the evidence was evaluated using the GRADE profiler, which showed that most of the evidence was downgraded due to the presence of risk of bias and inconsistency, with the quality of the evidence graded from very low to high (Supplementary Figure S11).
**DISCUSSION**			
Discussion	23a	Provide a general interpretation of the results in the context of other evidence.	In this study, we retrieved 3279 TCM RCTs for the treatment of DPN and finally included 95. The 95 RCTs involved 9 therapies, including TCM Decoction, Chinese Patent Medicine, acupuncture, electroacupuncture, TCM Decoction+ Acupuncture, TCM Decoction+ Chinese Herbal Footbath, TCM Decoction+ Chinese Medicine Fumigation, Acupoint Injection+ Chinese Medicine Fumigation, and Western medicine. We evaluated electromyography (common peroneal nerve and median nerve), FBG, 2hPG, TCSS, and total effective rate. Furthermore, our findings indicate that therapies related to TCM can enhance patients' blood glucose profiles by reducing FBG and 2hPG. It is widely recognized that effective management of blood glucose can alleviate clinical symptoms and halt the progression of the disease in patients with DPN. We evaluated neurophysiological indicators, motor and sensory conduction velocities of the common peroneal and median nerves (reflecting the recovery of nerve fiber function); glycemic indicators, including FBG and 2hPG (reflecting the control of blood

Section and Topic	Item #	Checklist item	Location where item is reported
			glucose); TCSS and the total effective rate (evaluating the improvement of clinical symptoms). The pairwise meta-analysis and NMA results consistently showed that the TCM-related therapies demonstrated more significant clinical advantages in improving nerve conduction velocity than Western medicine. TCM-related therapies effectively improve clinical symptoms in DPN patients. Specifically, in pairwise meta-analysis, TCM therapies significantly improved nerve conduction velocity in DPN patients; however, it is worth noting that the current study did not find any advantage of acupuncture over Western medicine in improving the conduction velocity of the median nerve. Furthermore, our findings indicate that therapies related to TCM can enhance patients' blood glucose profiles by reducing FBG and 2hPG. It is widely recognized that effective management of blood glucose can alleviate clinical symptoms and halt the progression of the disease in patients with DPN.
	23b	Discuss any limitations of the evidence included in the review.	Despite some valuable findings, this study has some limitations. First, the risk of bias and the quality of evidence of the included studies impacted the results. Some of the studies were defined as high-risk due to randomization and blinding; therefore, the quality of evidence was downgraded, affecting the credibility of the results. Indeed, implementing blinding in TCM RCTs faces inherent challenges. These difficulties stem from the fundamental conflict between TCM's holistic nature, individualized diagnosis, treatment approach, and the standardized requirements of RCTs. Particularly for non-pharmacological therapies like acupuncture, double-blinding is unattainable, which inevitably limits the strength of our evidence. When summarizing the characteristics of the included studies (Table 1), we observed that some RCTs failed to provide detailed descriptions of the duration and stage of DPN, with some even lacking data on age and disease duration. In studies utilizing TCM decoctions or Chinese patent medicine, the compositions of the herbal formulations were not standardized. Similarly, acupuncture studies lacked consistency in the acupoints selected. These factors contributed to substantial heterogeneity in some outcomes ($I^2 > 50\%$). Consequently, we performed subgroup analyses. These analyses revealed that neither different TCM herbal formulations nor varying acupuncture durations for nerve conduction velocity showed significant differences in effect sizes.
	23c	Discuss any limitations of the review processes used.	Regrettably, due to limitations in the original studies, we could not conduct subgroup analyses on other potential sources of heterogeneity, such as age, disease duration, and follow-up periods. As evident in Table 1, several studies lacked data on age, disease duration, and follow-up periods. The results are regrettable despite our efforts to contact authors for missing data. Therefore, our findings cannot provide reliable references for patients with DPN of different ages or disease durations. Furthermore, the long-term efficacy of TCM-related therapies for DPN remains uncertain due to the scarcity of follow-up data, highlighting an important area for future research. The NMA results indicate that the 95% confidence intervals for some of the two-by-two comparisons of interventions are quite wide, particularly evident when comparing TCM Decoction + Acupuncture with WM. Such wide intervals may be attributed to the limited sample sizes in the original studies or the inherent heterogeneity of the combination therapies, including variations in herbal prescriptions, dosages, needling techniques, and duration. This underscores the necessity for future studies involving larger sample sizes and more rigorously standardized intervention protocols to provide more conclusive evidence. Despite these uncertainties, sensitivity analysis indicated that our results are reliable. However, the limited number of original studies prevented a quantitative analysis of HbA1c. Additionally, the pairwise comparisons of TCSS, FBG, and 2hPG did not reach statistical significance. Consequently, this study cannot conclude whether TCM therapies improve the TCSS or glycemic control in DPN patients. Future well-designed studies are warranted to validate these findings. It is important to note that the safety data for this study were derived from only eight RCTs, all of which reported minor adverse events related to TCM therapies. Although TCM therapies are generally considered safe, this finding presents some limitations that could restrict the applicability and reproducibility of the results across a broader range of populations, including different subgroups and individuals with comorbidities. Therefore, high-quality studies are necessary to assess the safety of TCM therapies for treating DPN further and to provide more reliable clinical guidance for decision-makers in healthcare.
	23d	Discuss implications of the results for	Furthermore, our findings indicate that therapies related to TCM can enhance patients' blood glucose profiles by

Section and Topic	Item #	Checklist item	Location where item is reported
		practice, policy, and future research.	reducing FBG and 2hPG. It is widely recognized that effective management of blood glucose can alleviate clinical symptoms and halt the progression of the disease in patients with DPN. We evaluated neurophysiological indicators, motor and sensory conduction velocities of the common peroneal and median nerves (reflecting the recovery of nerve fiber function); glycemic indicators, including FBG and 2hPG (reflecting the control of blood glucose); TCSS and the total effective rate (evaluating the improvement of clinical symptoms). The pairwise meta-analysis and NMA results consistently showed that the TCM-related therapies demonstrated more significant clinical advantages in improving nerve conduction velocity than Western medicine. TCM-related therapies effectively improve clinical symptoms in DPN patients. Specifically, in pairwise meta-analysis, TCM therapies significantly improved nerve conduction velocity in DPN patients; however, it is worth noting that the current study did not find any advantage of acupuncture over Western medicine in improving the conduction velocity of the median nerve. Furthermore, our findings indicate that therapies related to TCM can enhance patients' blood glucose profiles by reducing FBG and 2hPG. It is widely recognized that effective management of blood glucose can alleviate clinical symptoms and halt the progression of the disease in patients with DPN.
**OTHER INFORMATION**			
Registration and protocol	24a	Provide registration information for the review, including register name and registration number, or state that the review was not registered.	This systematic review and meta-analysis will adhere to the latest 2020 guidelines for systematic reviews and PRISMA checklist (Supplementary Table S1). Prior to commencing this study, only a preliminary search was conducted to evaluate the feasibility and scope of potential studies.
	24b	Indicate where the review protocol can be accessed, or state that a protocol was not prepared.	The protocol for this systematic review was registered on PROSPERO (registration number CRD42024589159) on September 10, 2024.
	24c	Describe and explain any amendments to information provided at registration or in the protocol.	This initial search did not involve the formal screening process, data extraction, or analysis procedures.
Support	25	Describe sources of financial or non-financial support for the review, and the role of the funders or sponsors in the review.	This study was supported by the Science and Technology Program of the Education Department of Jilin Province (No. JJKH20250638K) and the Administration of Traditional Chinese Medicine of Jilin Province (No. 2024033). This study is gratefully acknowledged to all the authors of the RCTs who provided original data.
Competing interests	26	Declare any competing interests of review authors.	No competing interests in this study.
Availability of data, code and other materials	27	Report which of the following are publicly available and where they can be found: template data collection forms; data extracted from included studies; data used for all analyses; analytic code; any other materials used in the review.	This study is gratefully acknowledged to all the authors of the RCTs who provided original data.

From: Page MJ, McKenzie JE, Bossuyt PM, Boutron I, Hoffmann TC, Mulrow CD, et al. The PRISMA 2020 statement: an updated guideline for reporting systematic reviews. BMJ 2021;372:n71. doi: 10.1136/bmj.n71. This work is licensed under CC BY 4.0. To view a copy of this license, visit https://creativecommons.org/licenses/by/4.0/
