# Supplementary material for: Traditional Chinese medicine for diabetic peripheral neuropathy: a network meta-analysis
Source: Front Endocrinol (Lausanne). 2025 Aug 27;16:1596924. doi: 10.3389/fendo.2025.1596924 (PMC12420273; doi:10.3389/fendo.2025.1596924)
Supplement: Supplementary file 12 [file Table1.docx]

File S1. Search strategies.

| Database | Search term |
| --- | --- |
| **PubMed** | #1 “diabetic peripheral neuropathy” [Mesh] |
|  | #2 “diabetic peripheral neuropathy” [Title/Abstract] OR “diabetic neuropathy” [Title/Abstract] |
|  | #3 #1 OR #2 |
|  | #4 “Traditional Chinese Medicine” [Mesh] |
|  | #5 “Chinese Traditional Medicine” [Title/Abstract] OR “Traditional Tongue Diagnosis” [Title/Abstract] OR “Traditional Tongue Diagnoses” [Title/Abstract] OR “Traditional Tongue Assessment” [Title/Abstract] OR “Traditional Tongue Assessments” [Title/Abstract] OR “Tongue Assessment, Traditional” [Title/Abstract] OR “herbal medicine” [Title/Abstract] OR “Chinese medicine decoction” [Title/Abstract] OR “decoction” [Title/Abstract] OR “herbal medicine” [Title / Abstract] OR “Traditional Chinese medicine decoction” [Title/Abstract] OR “Chinese patent medicine”[Title/Abstract] OR “Chinese herbal medicine” [Title/Abstract] |
|  | #6 #4 OR #5 |
|  | #7“acupuncture and moxibustion” [Mesh] |
|  | #8 “acupuncture” [Title/Abstract] OR “electroacupuncture” [Title/Abstract] OR “electro-acupuncture” [Title/Abstract] OR “acupuncture analgesia” [Title/Abstract] OR “acupuncture treatment” [Title/Abstract] OR “acupuncture treatments” [Title/Abstract] OR “treatment, acupuncture” [Title/Abstract] OR “therapy, acupuncture” [Title/Abstract] OR “trigger point” [Title/Abstract] OR “acupuncture Point” [Title/Abstract] OR “point, acupuncture” [Title/Abstract] OR “points, acupuncture” [Title/Abstract] OR “moxibustion” [Title/Abstract] OR moxabustion [Title/Abstract] |
|  | #9 “massage”[Mesh] |
|  | #10 massage [Title/Abstract] OR massage therapy [Title/Abstract] OR therapy massage [Title/Abstract] OR tuina [Title/Abstract] |
|  | #11 #9 OR #10 |
|  | #12 “Randomized Controlled Trial” [Publication type] OR “random allocation” [Publication type] OR “allocation” [Publication type] |
|  | #13 #3 AND #6 AND #11 AND #12 |
| **Cochrane Library** | #1 MeSH descriptor: [diabetic peripheral neuropathy] explode all trees |
|  | #2 MeSH descriptor: [Chinese Traditional Medicine] explode all trees |
|  | #3 #1 OR #2 |
|  | #4 (Medicine, Chinese Traditional):ti,ab,kw OR (Traditional Tongue Diagnosis):ti,ab,kw OR (Traditional Tongue Diagnoses):ti,ab,kw OR (Traditional Tongue Assessment):ti,ab,kw OR (Traditional Tongue Assessments):ti,ab,kw OR (Tongue Assessment, Traditional):ti,ab,kw OR (herbal medicine):ti,ab,kw OR (Traditional Chinese medicine decoction):ti,ab,kw OR (decoction):ti,ab,kw OR (Chinese patent medicine):ti,ab,kw OR (acupuncture):ti,ab,kw OR (electroacupuncture):ti,ab,kw OR (electro-acupuncture):ti,ab,kw OR (acupuncture treatment):ti,ab,kw OR (trigger point):ti,ab,kw OR (moxibustion):ti,ab,kw OR (tuina):ti,ab,kw OR (moxibustion):ti,ab,kw |
|  | #5 MeSH descriptor: [randomized controlled trial] explode all trees |
|  | #6 MeSH descriptor: [randomized controlled trials] explode all trees |
|  | #7 MeSH descriptor: [randomized controlled trial] explode all trees |
|  | #8 MeSH descriptor: [random allocation] explode all trees |
|  | #9 MeSH descriptor: [allocation] explode all trees |
|  | #10 #5 OR #6 OR #7 OR #8 OR #9 |
|  | #11 #3 AND #4 AND #10 |
| **Web of science** | #1 TS=(diabetic peripheral neuropathy) OR TS=(diabetic neuropathy) |
|  | #2 TS=(Traditional Chinese Medicine) OR TS=(Chinese Traditional Medicine) OR TS=(Traditional Tongue Diagnosis) OR TS=(Traditional Tongue Diagnoses) OR TS=(Traditional Tongue Assessment) OR (Traditional Tongue Assessments) OR TS=(Tongue Assessment, Traditional) OR TS=(herbal medicine) OR TS=(Traditional Chinese medicine decoction) OR TS=(Chinese patent medicine) OR TS=(Traditional Chinese medicine decoction) OR TS=(Chinese herbal medicine) OR TS=(acupuncture) OR TS=(electroacupuncture) OR TS=(electro-acupuncture) OR TS=(acupuncture analgesia) OR TS=(acupuncture treatment) OR TS=(acupuncture treatments) OR TS=(treatment, acupuncture) OR TS=(therapy, acupuncture) OR TS=(trigger point) OR TS=(acupuncture Point) OR TS=(point, acupuncture) OR TS=(points, acupuncture) OR TS=(moxibustion) OR TS=(moxabustion) OR TS=(massage) OR TS=(massage therapy) OR TS=(tuina) |
|  | #3 TS=(Randomized Controlled Trial) OR TS=(Randomized Controlled Trials) OR TS=(random allocation) OR TS=(allocation) |
|  | #4 #1 AND #2 AND #3 |
| **Embase** | #1 'diabetic peripheral neuropathy'/exp |
|  | #2 'diabetic peripheral neuropathy':ti,ab OR 'diabetic neuropathy ':ti,ab |
|  | #3 #1 OR #2 |
|  | #4'Traditional Chinese Medicine'/exp OR 'Chinese Traditional Medicine':ab,ti OR 'Traditional Tongue Diagnosis':ti,ab OR 'Traditional Tongue Diagnoses':ti,ab OR 'Traditional Tongue Assessment':ti,ab OR 'Traditional Tongue Assessments':ab,ti OR 'Tongue Assessment, Traditional':ti,ab OR 'Traditional Tongue Diagnoses':ti,ab OR 'Traditional Tongue Assessment':ti,ab OR 'herbal medicine':ti,ab OR 'Traditional Chinese medicine decoction':ab,ti OR 'Chinese herbal medicine':ti,ab OR 'acupuncture':ti,ab OR 'electroacupuncture':ti,ab OR 'electro-acupuncture':ab,ti OR 'acupuncture treatments':ti,ab OR 'acupuncture treatment':ti,ab OR 'trigger point':ti,ab OR 'moxibustion ':ti,ab OR 'tuina':ti,ab |
|  | #5'randomized controlled trial'/exp OR 'randomized controlled trials'/exp OR 'random allocation'/exp OR 'allocation'/exp |
|  | #6 #3 and #4 and #5 |
| **Medline** | #1 diabetic peripheral neuropathy [Mesh] |
|  | #2 diabetic peripheral neuropathy [tiab] OR diabetic neuropathy [tiab] |
|  | #3 #1 OR #2 |
|  | #4 Traditional Chinese Medicine* [tiab] OR Chinese Traditional Medicine [tiab] OR Traditional Tongue Diagnosis [tiab] OR Traditional Tongue Diagnoses [tiab] OR Chinese Traditional Medicine [tiab] OR Traditional Tongue Diagnosis [tiab] OR Traditional Tongue Diagnoses [tiab] OR Chinese Traditional Medicine [tiab] OR Traditional Tongue Assessment [tiab] OR herbal medicine [tiab] OR Traditional Chinese medicine decoction [tiab] OR Chinese herbal medicine [tiab] OR acupuncture [tiab] OR electroacupuncture [tiab] OR electro-acupuncture [tiab] OR acupuncture treatment [tiab] OR trigger point [tiab] OR moxibustion [tiab] OR massage [tiab] OR tuina [tiab] |
|  | #5 randomized controlled trial[publication type] OR controlled clinical trial[publication type] OR randomized[tiab] OR placebo[tiab] OR randomly[tiab] OR trial[tiab] OR groups[tiab] |
|  | #6 #3 AND #4 AND #5 |
| **CNKI** | #1 ( 主题 = 糖尿病周围神经病 或者 题名 = 糖尿病周围神经病变 或者 v_subject= 中英文扩展 (糖尿病周围神经病) 或者 title= 中英文扩展 (糖尿病周围神经病)) ( 模糊匹配 ) |
|  | #2 ( 主题 = 消渴痹症 或者 题名 = 消渴痹症 或者 v_subject= 中英文扩展 (消渴痹症) 或者 title= 中英文扩展 (消渴痹症)) ( 模糊匹配 ) |
|  | #3 ( 主题 = 中医药 或者 题名 = 中医药 或者 v_subject= 中英文扩展 ( 中医药 ) 或者 title=中英文扩展 ( 中医药 )) ( 模糊匹配 ) |
|  | #4 ( 主题 = 中医 或者 题名 = 中医 或者 v_subject= 中英文扩展 ( 中医) 或者 title=中英文扩展 ( 中医 )) ( 模糊匹配 ) |
|  | #5 ( 主题 = 中药 或者 题名 =中药 或者 v_subject= 中英文扩展 (中药) 或者 title=中英文扩展 (中药)) ( 模糊匹配 ) |
|  | #6 ( 主题 = 汤剂 或者 题名 =汤剂或者 v_subject= 中英文扩展 (汤剂) 或者 title=中英文扩展 (汤剂)) ( 模糊匹配 ) |
|  | #7 ( 主题 = 汤 或者 题名 =汤 或者 v_subject= 中英文扩展 (汤) 或者 title=中英文扩展 (汤)) ( 模糊匹配 ) |
|  | #8 ( 主题 = 方 或者 题名 =方 或者 v_subject= 中英文扩展 (方) 或者 title=中英文扩展 (方)) ( 模糊匹配 ) |
|  | #9 ( 主题 = 散 或者 题名 =散 或者 v_subject= 中英文扩展 (散) 或者 title=中英文扩展 (散)) ( 模糊匹配 ) |
|  | #10 ( 主题 = 中成药 或者 题名 =中成药 或者 v_subject= 中英文扩展 (中成药) 或者 title=中英文扩展 (中成药)) ( 模糊匹配 ) |
|  | #11 #2 OR #3 OR #4 OR #5 OR #6 OR #7 OR #8 OR #9 OR #10 |
|  | #12 ( 摘要 = 随机 或者 abstract = 中英文扩展 ( 随机 )) ( 模糊匹配 ) |
|  | #13 ( 摘要 =RCT 或者 abstract = 中英文扩展 (RCT)) ( 模糊匹配 ) |
|  | #14 #11 OR #12 |
|  | #15 #1 AND #11 AND #14 |
