# Supplementary material for: Traditional Chinese medicine for diabetic peripheral neuropathy: a network meta-analysis
Source: Front Endocrinol (Lausanne). 2025 Aug 27;16:1596924. doi: 10.3389/fendo.2025.1596924 (PMC12420273; doi:10.3389/fendo.2025.1596924)
Supplement: Supplementary file 13 [file Table2.docx]

| Domain | Criteria | | |
| --- | --- | --- | --- |
|  | Low risk | High risk | Unclear risk |
| Random sequence generation | Truly random, unpredictable methods were used (e.g., random number tables, computerized random number generators). | Non-randomized or predictable methods were used (e.g., by date of birth, medical record number, alternate assignment, odd-even date). | The trial was described as “randomized,” but the exact methodology was not specified and could not be determined. |
| Allocation concealment | Strict precognition prevention measures were used (e.g., numbered sealed opaque envelopes). | Assignment sequences were visible to the recruiter (e.g., open list, unsealed envelopes, obvious patterns by date of birth, etc.). | Methods of allocation concealment were not described or were inadequately described. |
| Blinding of patients and personnel | Effective blinding of subjects and blinding of blinders were not implemented, but outcome indicators were unlikely to be affected (e.g., objective hard endpoints such as mortality). | Blinding was not implemented or was disrupted, and outcome indicators were likely to be affected (especially subjective endpoints). | Blinding was not sufficiently described to allow judgment. |
| Blinding of outcome assessment | Effective blinding of outcome assessors was implemented, or blinding was not implemented, but outcome assessment was unlikely to be affected (e.g., objective outcomes). | Blinding of the outcome assessor was not implemented or was disrupted, and the assessment was likely to be influenced by the knowledge of the allocation (especially for subjective outcomes). | Blinding of outcome assessors was not described. |
| Incomplete outcome data | There was little missing data, and it was balanced across groups; the reasons for missing data were unlikely to be related to the true outcome; appropriate methodology was used to deal with missing data. | There was a lot of missing data or not balanced across groups; the reasons for missing data were likely to be related to the true outcome (e.g., many side effect withdrawals in the trial group), and inappropriate methodology was used. | Insufficient reporting of missed visits/withdrawals or methods of handling missing data. |
| Selective reporting | Pre-published program or registry information was available, and all prespecified primary and secondary outcomes were reported in the report; no evidence of selective reporting. | No prespecified primary outcomes were reported; an unprespecified outcome was reported as the primary conclusion; omission of important outcomes; reporting in a manner that resulted in bias. | Study protocol information was unavailable; there was insufficient information to make a judgment. |
| Other biases | The study was not at significant risk of bias outside the above domains. | Other significant biases exist (e.g., uncorrected baseline imbalance, study-specific contextual issues such as undue funder influence). | Other biases may be present, but the information is insufficient. |

Supplementary Table S2. Criteria for assessing the risk of bias.
